# Cyclic Peptides in Modern Drug Discovery: Trends and Therapeutic Directions

**DOI:** 10.1021/acs.jmedchem.6c01522

**Published:** 2026-07-21

**Authors:** Trupti Thite, Kavita A Iyer, Preeti Jain, Janet M Sasso, Rumiana Tenchov, Sudarshan Murmu, Mahaveer Surendranath, Ankush Maind, Umesh T Balande, Qiongqiong Angela Zhou

**Affiliations:** † ACS International Pvt. Ltd., Pune 411044, India; ‡ 2645 CAS, A Division of the American Chemical Society, Columbus, Ohio 43210, United States

## Abstract

Peptide therapeutics are an important drug modality due to their high specificity, favorable safety, and expanding design potential. Among them, cyclic peptides occupy a space between small molecules and biologics, offering improved rigidity, stability, and target engagement. This report analyzes trends in cyclic peptide research using data from the CAS Content Collection over the past two decades. Results show a steady rise in academic publications and patents, reflecting growing interest across discovery and development. Notably, oral administration is gaining attention, indicating progress toward addressing long-standing bioavailability challenges. Beyond delivery, we examine how peptide and cyclization types, along with specific chemical modifications, relate to administration routes, therapeutic indications, and molecular targets. We also assess physicochemical properties to understand how molecular features influence developability. Together, these insights provide a comprehensive view of the evolving cyclic peptide landscape and emerging principles guiding their future development.

## Graphical abstract

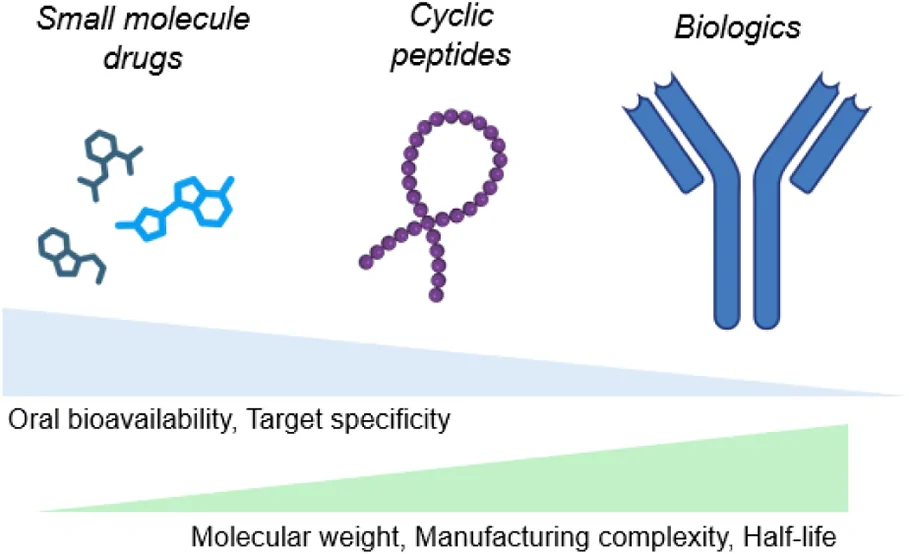

## 1 Introduction

Peptide therapeutics have emerged as a promising modality in human medicine over the past several decades, owing to their high specificity, favorable safety profiles, and expanding chemical design space.^1,2^ As reflected by the increase in the number of publications (Supplementary Figure S1), their impact has become increasingly evident exemplified by the success of semaglutide, which has achieved unprecedented global sales and has reshaped the landscape of metabolic disease therapeutics.^3^ Among all U.S. FDA-approved peptide therapeutics, about 25% belong to the category of cyclic peptides.^4^ Cyclic peptides are a class of polypeptides in which the amino acid chain is covalently closed to form a ring structure, through various cyclization strategies. This topology distinguishes them from linear peptides and generally imparts enhanced conformational rigidity, proteolytic stability, and better binding affinity. Typically, cyclic peptides occupy an intermediate molecular weight (MW) range of ∼500–3000 Da, situating them between small molecules and large biologics in size and complexity.^5^ Their cyclic architecture imparts several advantages, including enhanced resistance to enzymatic degradation, improved target binding, and, crucially, the potential for oral bioavailability, a long-standing hurdle in peptide-based therapeutics.^6^ These structurally constrained compounds occupy a unique chemical space, combining the high specificity and affinity of large biologics with the favorable pharmacokinetic properties of smaller molecules.^7^

The global cyclic peptide market is experiencing steady growth, driven by their unique structural advantages compared to linear peptides. According to recent reports,^8,9,10^ the market was valued at approximately USD 3.16 billion in 2024 and was projected to reach USD 3.35 billion in 2025, with a compound annual growth rate (CAGR) of 6.5–6.7%, ultimately surpassing USD 5.3 billion by 2032. Biopharmaceutical applications dominate, particularly in oncology, autoimmune disorders, and infectious diseases, supported by advancements in solid-phase synthesis, display screening platforms (like mRNA display and phage display), and computational design. Key players include Bicycle Therapeutics, Merck, Bachem, and Apellis Pharmaceuticals, while emerging uses in diagnostics, environmental protection, and biosensing are expanding the market footprint. This growth reflects cyclic peptides’ role as a cornerstone modality in next-generation biologics development.

In this paper, we explore data from the CAS Content Collection,^11^ the largest human-curated collection of published scientific information, in an effort to provide a comprehensive overview of cyclic peptides with a focus on their therapeutic potential. In parallel with cyclic peptides, other constrained modalities such as macrocyclic small molecules, miniproteins, and peptidomimetics have also been developed to address challenging biological targets through complementary structural strategies. In the current report, our analysis focuses on cyclic peptides, and our findings indicate that the oral route of delivery is gaining increasing attention, as reflected by a steady rise in publications over the past several years. Beyond delivery, we further examine how specific peptide properties like peptide types, cyclization types, and certain peptide modifications co-occur with therapeutic areas, potential molecular targets, and administration routes. Beyond structural analyses, this manuscript also examines the physicochemical properties of cyclic peptides, followed by an overview of the current clinical landscape and emerging developmental trends. Together, it is our hope that these insights offer a comprehensive view that can be helpful to medicinal chemists in the rational design and prioritization of cyclic peptides by linking structural, physicochemical, and therapeutic trends observed across current development efforts.

## 2 Trends in Cyclic Peptide Research—Insights from CAS Data

Our analysis of cyclic peptide-related publications (2006–2025) shows steady growth with journal articles comprising 66% (15,429) and patents 34% (8,042) of the total output (Figure 2A). Activity accelerated from 2015, peaking in 2021–2024. Notably, patents surpassed journal articles in 2023–2024, indicating increased commercialization focus continuing into 2025. Geographically, China dominates research output, followed by the United States, Japan, Germany, India, and the United Kingdom (Figure 2B). China’s patent-heavy profile reflects strategic emphasis on commercial applications.

CAS section analysis, resulting from robust indexing efforts by CAS analysts (Figure 2C), shows pharmaceuticals dominate with a >2-fold increase from 2022 to 2024. Significant growth in immunochemistry, biochemical genetics, pharmacology, and biochemical methods indicates expanding applications beyond therapeutics into biochemical tools and industrial biotechnology.

Supplementary Figures S2A and S2B show the leading commercial and noncommercial patent assignees, with notable innovations in cyclic peptide-based drug delivery systems,^12^ macrocyclic scaffolds for improved oral bioavailability,^13^ and peptide conjugates for targeted cancer therapy,^10,14,15^ reflecting the translational potential of cyclic peptide research.

Publication output and citation analysis (Figures S3 and S4) reveal leading organizations engaged in cyclic peptides-related research. While University of Queensland and University of California lead in research output (number of journal publications), University of California leads in terms of research impact (number of citations). When viewed from the lens of both output and impact, Scripps Research Institute, Technische Universität München, and Ohio State University achieve highest citation rates despite lower output (Figure S4A) with institutions in the US seemingly dominating this metric. Similar analysis of scientific journals (Figure S5) indicates that Nature Chemical Biology, PNAS, and Journal of the American Chemical Society achieve highest per-article citation rates (Figure S4B). These insights can help researchers identify journals that combine visibility with influence, guiding strategic decisions for disseminating impactful work.

## 3 Cyclic Peptides as Therapeutics—Analysis Based on CAS Data

To investigate emerging trends in cyclic peptide therapeutics, we conducted a detailed analysis of substance data (2020–2025) from the CAS REGISTRY. Our analysis was restricted to the 2020–2025 period to capture recent and translationally relevant trends in cyclic peptide research. As is evident in Figure 2A, patent activity increased markedly from 2020 onward and started outpacing journal articles starting in 2023, indicating an acceleration in innovation. Importantly, patents filed during this period are increasingly concentrated in pharmaceutically relevant areas as seen from the CAS section data (Figure 1C), reflecting a shift toward therapeutic development. Substances from our data set indexed with CAS roles therapeutic (THU), pharmacological (PAC), or pharmacokinetic (PKT) were considered for analysis. Their corresponding SMILES representations were processed using RDKit^16^ for structural evaluation. By applying this workflow (detailed in the Methods section in Supporting Information), we identified 46,574 cyclic peptides. Classification was based on the following criteria: (1) The molecule contains at least one ring (has_any_ring = True), (2) the molecule possesses ≥2 amide bonds overall (len­(amide_bidx) ≥ 2), and (3) at least one ring incorporates ≥2 backbone-like amide bonds, with both termini adjacent to α-carbons. A peptide was considered cyclic peptide only if all these three conditions were satisfied.

We then analyzed the major therapeutic areas mentioned in our data set and looked at the distribution of these cyclic peptides across them. The analysis shows a clear dominance in the field of oncology (Figure 1D). Infectious and inflammatory diseases represent the next major clusters. Autoimmune and cardiovascular diseases also feature prominently, though with substantially fewer publications compared to cancer. Metabolic and neurodegenerative disorders represent smaller but emerging areas of exploration. Supplementary Figure S6A shows their distribution across journal articles versus patent families. Overall, these trends highlight that while cyclic peptides are being investigated across a broad therapeutic landscape, cancer and infectious diseases remain the primary drivers of research activity (based on volume of documents).

**Figure 1:**
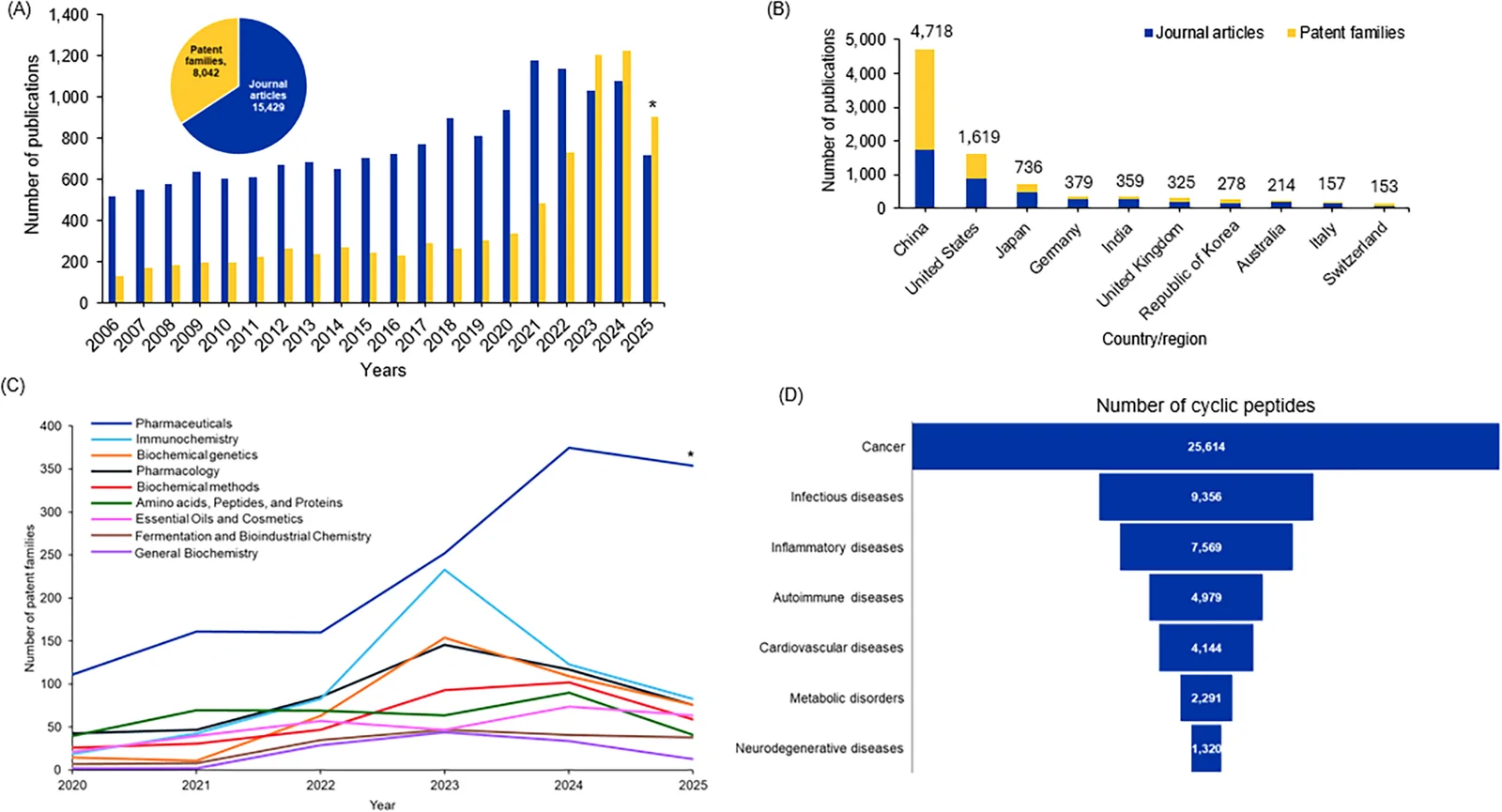
(A) Year-wise and (B) country/region-wise distribution of journal and patent publications related to cyclic peptides. (C) Year-wise trend of patents related to cyclic peptides based on their respective CAS sections. (D) Major therapeutic areas in cyclic peptide research. Each bar shows number of cyclic peptides associated with a particular therapeutic area (data from 2020 to 2025). Source: CAS Content Collection, *Data until August 2025.

**Figure 2:**
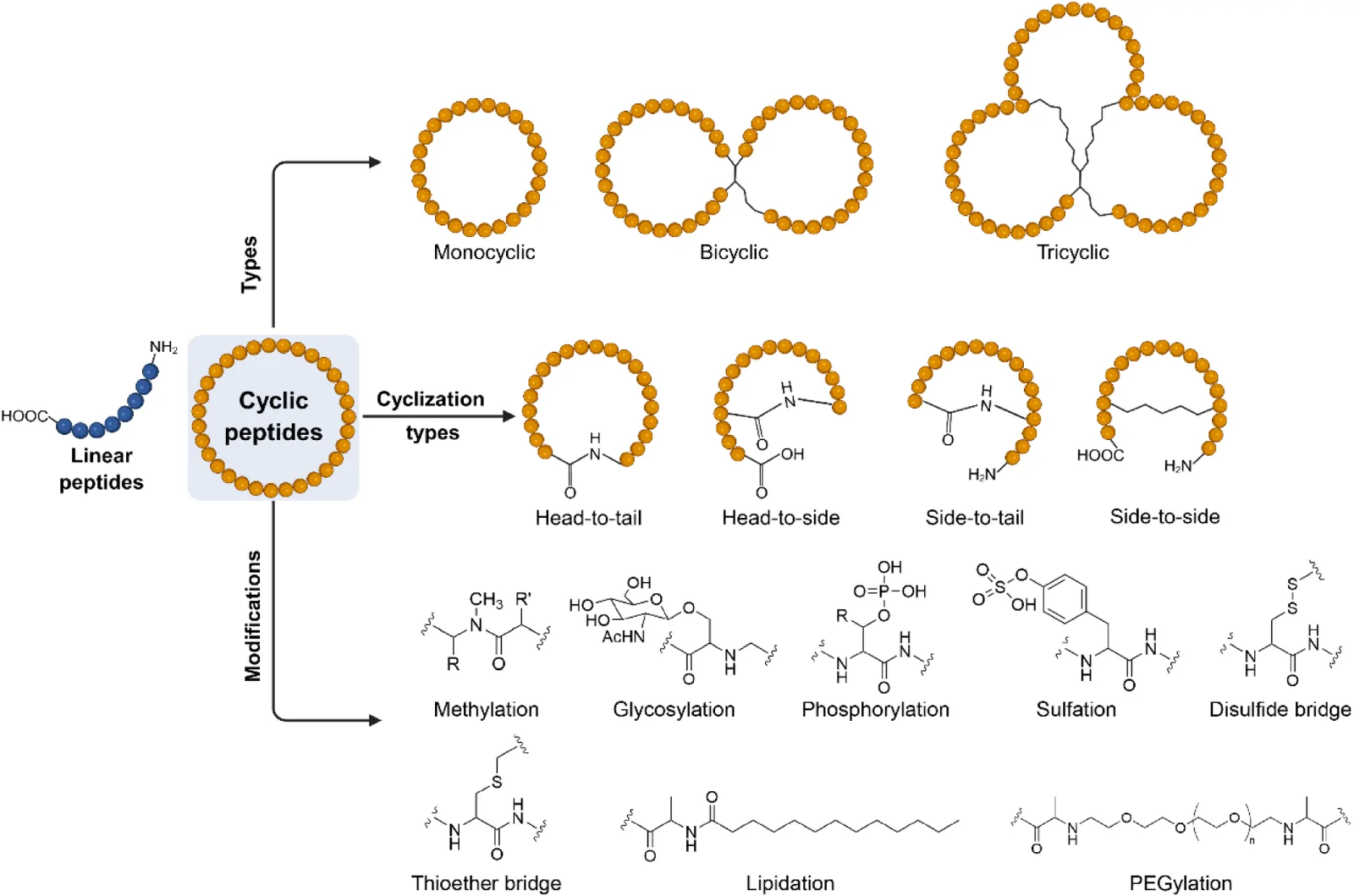
Schematic representation of the types of cyclic peptides, their cyclization types, and a few common modifications. Figure created partially using www.BioRender.com.

Having identified nearly 46,000 cyclic peptides, our next step was to analyze these identified peptides using RDKit^16^ to identify the types of cyclic peptides (based on the number of amino acids they contain), the type of cyclization present in a given peptide, and which if any modifications are present on these peptides.

### 3.1 Types of Cyclic Peptides

Cyclic peptides represent a structurally diverse class of biomolecules that can be categorized according to their source, the nature of the bonds forming the ring, and the number of amino acid residues involved. From a source perspective, they may be either naturally occurring, produced by microorganisms, plants, or marine organisms,^17^ or synthetically generated through chemical synthesis for tailored pharmacological applications.^18^ Based on the type of bond, cyclic peptides can be divided into homodetic, isopeptide, and depsipeptide classes. Homodetic cyclic peptides, such as cyclosporine A,^19^ are characterized by rings composed exclusively of standard peptide bonds between the α-carboxyl group of one residue and the α-amino group of another. Isopeptidic cyclic peptides, exemplified by microcystin^20^ and bacitracin,^21^ contain at least one non-α amide linkage, often involving side chains, which impart structural diversity and unique biological activity. Depsipeptides, including aureobasidin A,^22^ kahalalide F,^23^ and didemnin B,^24^ feature at least one ester (lactone) linkage in place of an amide bond, frequently formed between the C-terminal carboxyl group and the hydroxyl side chain of serine or threonine residues, and are notable for their potent pharmacological properties.

Classification by ring size further highlights the functional diversity of cyclic peptides. Cyclic dipeptides, or diketopiperazines, are the simplest members, typically rigid and resistant to proteolysis.^25^ Tripeptides, slightly larger and more conformationally flexible, have been reported to exhibit antioxidant and anti-inflammatory properties.^26^ Tetrapeptides, with reduced ring strain, display enhanced stability and are widely studied for receptor modulation and pharmacological activity.^27^ Pentapeptides strike a balance between conformational diversity and stability, making them attractive scaffolds for drug design with improved bioavailability.^28^ In general, tetra- and pentapeptides are considered the threshold for reliable stabilization, as smaller rings such as di- and tripeptides may suffer from ring strain, while larger macrocyclic structures gain conformational stability and functional versatility.^29^ Macrocyclic peptides, as well as bicyclic and polycyclic architectures, exhibit exceptional stability and highly specific binding properties, exemplified by molecules such as vancomycin,^30^ daptomycin,^31^ and defensins.^32^ The term “macrocyclic peptide” has been vaguely defined in the literature, and there is no known stringent cutoff on the number of amino acids in a macrocyclic peptide. Here, based on previously published reports^33,34,35^ and opinions of experts in the field,^36^ we have considered any cyclic peptide with six or more amino acids as a macrocyclic peptide. Bicyclic or polycyclic peptides have complex structures with two (bi-) or more (poly-) cyclic rings^37^ (Figure 2). Collectively, these classifications underscore the structural and functional richness of cyclic peptides, which continue to serve as valuable templates in drug discovery, chemical biology, and therapeutic development.

### 3.2 Types of Cyclization

Cyclic peptides exhibit remarkable structural diversity, and the mode of cyclization strongly influences their stability and biological properties. Head-to-tail cyclization is the most prevalent, forming a closed ring between the N-terminal amine and C-terminal carboxyl group (Figure 2). This orientation eliminates free termini, conferring resistance to exopeptidase degradation and often enhancing membrane permeability and intracellular delivery.^38^ Head-to-side and side-to-tail cyclizations introduce alternative ring closures by linking termini to reactive side chains (Figure 2), which can fine-tune conformational rigidity and surface exposure of functional groups.^39^ Side-to-side cyclization, such as disulfide bond formation between cysteine residues, stabilizes secondary structures and allows reversible redox control, making it particularly useful in mimicking natural peptide hormones and toxins.^40^

Beyond these canonical strategies, mixed-mode cyclization combines multiple linkages to create hybrid architecture with enhanced complexity. Such designs can lock peptides into highly defined conformations, improving receptor selectivity, bioavailability, and thermal stability. By carefully choosing the cyclization orientation, chemists can modulate peptide solubility, resistance to enzymatic breakdown, and overall pharmacokinetic behavior. A more critical comparison of these strategies is summarized in Table 1.

**Table 1:** Comparative Overview of Major Cyclic Peptide Cyclization Strategies, Information Summarized from Published Literature^39,41,42,43,44,45,46^

Cyclization strategy	Conformational rigidity vs flexibility	Metabolic soft spots introduced or mitigated	Overall metabolic stability	Synthetic complexity	Scalability and manufacturability
**Head-to-tail**	Highest global rigidity due to full backbone closure	Removes N- and C-terminal exopeptidase sites; internal amide cleavage still possible	Very high	Moderate (risk of oligomerization)	Excellent; SPPS-compatible; clinically proven
**Side-to-tail**	Intermediate rigidity; local constraint with retained backbone flexibility	N-terminus remains vulnerable; ester linkages are metabolic soft spots	Medium–high (linkage-dependent)	Moderate (orthogonal protection required)	Good; routinely scalable for lead optimization
**Head-to-side**	Intermediate rigidity; directionally biased constraint	C-terminus exposed to carboxypeptidases; stable if amide linkage used	Medium	Moderate	Moderate
**Side-to-side**	Localized rigidity only; high global flexibility	Both termini exposed; disulfides redox-labile; thioethers/lactams more stable	Low-medium	Variable (depends on linkage)	Variable
**Mixed strategies**	Maximal preorganization; risk of over-rigidification	Multiple liabilities masked; novel linkages may introduce new metabolic routes	Very high	High (usually multistep synthesis)	Poor-moderate

We analyzed cyclic peptides with CAS roles—THU, PAC, and PKT—from the CAS Content Collection using RDKit^16^ to classify them based on the number of amino acids they contain and cyclization type (Figure 3). For this, we selected the ring with the highest number of amide bonds (most likely the main macrocycle) followed by counting all amide bonds in that ring (ignoring the α-carbon filter for size naming). Lastly, we applied the following classification scheme: if the peptide contains 2 amides → cyclic dipeptide; 3 amides → cyclic tripeptide; 4 amides → cyclic tetrapeptide, 5 amides → cyclic pentapeptide; and ≥6 amides → macrocyclic peptide. If there were more than one amide-containing rings, then the peptides were classified as bicyclic or polycyclic.

**Figure 3:**
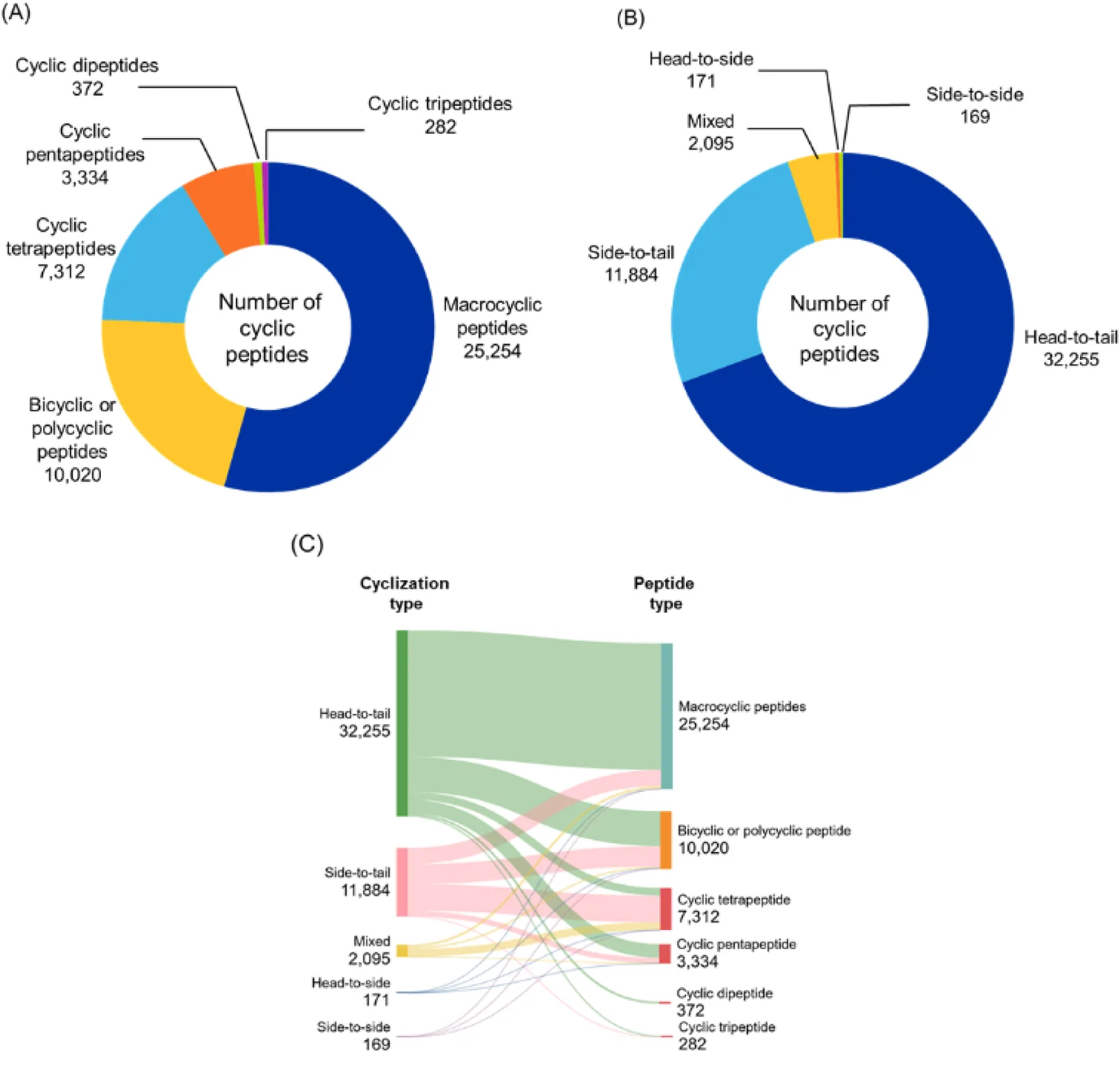
Distribution of identified cyclic peptides based on their (A) type and (B) cyclization type. (C) Sankey graph showing co-occurrence between peptide type and cyclization type. Only cyclic peptides indexed with CAS roles THU, or PAC, or PKT were included for the analysis for the period 2020–2025. Source: CAS Content Collection.

To determine the cyclization type, carbonyl carbon and amide nitrogen atoms were identified and checked for adjacency. If amide N is adjacent to an α-carbon, it was marked as head (backbone). If carbonyl C is adjacent to an α-carbon, it was marked as tail (backbone). The cyclization type was determined based on whether the two ends involved both as backbone (head-to-tail), one N backbone and other as C side-chain (head-to-side), one N side-chain and other as C backbone (side-to-tail), and both ends as side-chains (side-to-side).

Our analysis showed that, macrocyclic peptides were the most prevalent, accounting for more than half of the identified cyclic peptides, followed by bicyclic and polycyclic structures (Figure 3A). In contrast, cyclic dipeptides and tripeptides were least common, suggesting that larger peptides are generally preferred for therapeutic applications due to their superior stability and functional versatility. The distribution of cyclic peptide types across patents filed by leading commercial assignees (summarized in Supplementary Table S1) shows Bicycle Therapeutics as leaders in bicyclic or polycyclic peptides, whereas Bristol-Myers Squibb and Merck show most association with macrocyclic peptides.

Among cyclization strategies, head-to-tail cyclization was most dominant, accounting for nearly 2/3rd of the identified cyclic peptides, followed by side-to-tail and mixed cyclizations. Head-to-side and side-to-side account for a very small fraction of cyclic peptides indicating that these cyclization types may not be preferred or as explored (Figure 3B). Evaluation of patent portfolio from leading commercial assignees (Supplementary Table S2) shows strong co-occurrence of side-to-tail cyclized peptides in patents from Bicycle Therapeutics and head-to-tail cyclized peptides in patents from Bristol-Myers Squibb and Merck. Co-occurrence analysis between peptide type and cyclization strategy revealed that most head-to-tail cyclized peptides form macrocyclic architectures, whereas side-to-tail and mixed cyclizations contribute more diversely to bicyclic/polycyclic structures and smaller ring sizes such as tetrapeptides and pentapeptides (Figure 3C). Supplementary Figures S6B and S6C show the number of documents (journal articles versus patent families) for different peptide types and cyclization types, respectively. Both show dominance of journal articles compared to patents. Overall, these findings highlight clear co-occurrence patterns between cyclization strategies and observed peptide architectures within the analyzed data set.

We also looked at the MW distribution of these cyclic peptides, stratified by ring size and topology (Supplementary Figure S7). Most reported cyclic peptides cluster in the 1,000–2500 Da range, with macrocyclic peptides constituting the dominant class across these MWs, whereas bicyclic or polycyclic peptides appear comparatively less frequently but are preferentially represented at higher MW.

### 3.3 Cyclization Bonds and Chemical/Bioconjugate Modifications

Cyclic peptides can be stabilized through diverse cyclization bonds such as disulfide, ether, thioether, ester, and thioester linkages, each imparting unique structural and functional properties. Disulfide bonds, formed between cysteine residues, are among the most common and confer conformational rigidity, though they can be redox-sensitive.^47,48^ Thioether and ether linkages provide enhanced chemical stability compared to disulfides, as they are resistant to reduction and proteolytic cleavage.^49,50^ Ester bonds in cyclic peptides enhance protease resistance, solubility, and conformational control, making them useful for improving stability and drug-like properties.^51^ Thioester bonds, meanwhile, play a key role in natural biosynthesis and synthetic cyclization by enabling acyl shifts, facilitating efficient macrocyclization, and mimicking biological pathways.^52^ Furthermore, amide-to-ester substitutions in cyclic peptides can also improve their membrane permeability.^53^ These cyclization bonds directly influence the therapeutic potential of cyclic peptides by modulating stability, bioavailability, and target affinity.

Chemical or bioconjugate modifications on peptides constitute a fundamental strategy in the rational design of novel peptide entities and the expansion of their functional repertoire. By applying well established chemical methodologies, it is possible to modulate key physicochemical parameters, including net charge, hydrophobicity, conformational flexibility, amphiphilicity, and sequence composition that collectively govern peptide stability and biological performance.^54^ Some important modifications and their significance are summarized in Table 2. Such targeted modifications enable researchers to overcome intrinsic limitations of native peptides, thereby improving pharmacokinetic behavior, enhancing biological activity, and broadening therapeutic applicability.

**Table 2:** Peptide Modifications and Their Significance

Modification	Significance
Methylation	Improves oral bioavailability^55^
	Improves protease resistance^56^
	Increases membrane permeability^57^
Glycosylation	Improves solubility and stability, improves half-life^58^
	Enhances target binding^59^
	Reduces immunogenicity^60^
Lipidation	Increases membrane permeability^61^
	Improves receptor selectivity and potency^62^
	Increases enzymatic stability^63^
Phosphorylation	Impacts peptide conformation and interactions with target proteins^64^
Sulfation	Enhances receptor binding and improves aqueous solubility^65^
PEGylation	Reduces renal clearance^66^
	Prolongs circulation half-life^67^

Our analysis focused on cyclization bonds (disulfide and thioether) and chemical modifications listed in Table 2. Disulfide bonds were the most common, followed by thioether linkages. Among chemical modifications, *N*-methylation was most prevalent, followed by complex modifications (more than one modification) and lipidation. Glycosylation, PEGylation, sulfation, and phosphorylation were comparatively rare, likely because these modifications are more specialized and often used to enhance solubility, bioavailability, or targeting rather than being broadly applied across peptide classes (Figure 4A). Complex modifications, thioether bonds, disulfide bonds, and PEGylation are mentioned more frequently in patents than in journal articles (Figure S6D). An analysis of patent filings from leading commercial assignees (Supplementary Table S3) shows distinct, company-specific preferences in cyclic peptide modification strategies. Thioether-mediated cyclization predominates as compared to disulfide linkages. *N*-methylation and more complex modifications occur more frequently in portfolios of large pharmaceutical companies, while lipidation, glycosylation, sulfation, and PEGylation appear sporadically, consistent with their use in later-stage optimization. Co-occurrence analysis between modifications and peptide type revealed that larger peptides—macrocyclic and bicyclic/polycyclic—exhibit diverse and extensive modifications, whereas smaller peptides tend to have fewer modifications (Figure 4B). This trend suggests that structural complexity provides more opportunities for functional tailoring, which is critical for optimizing therapeutic performance.

**Figure 4:**
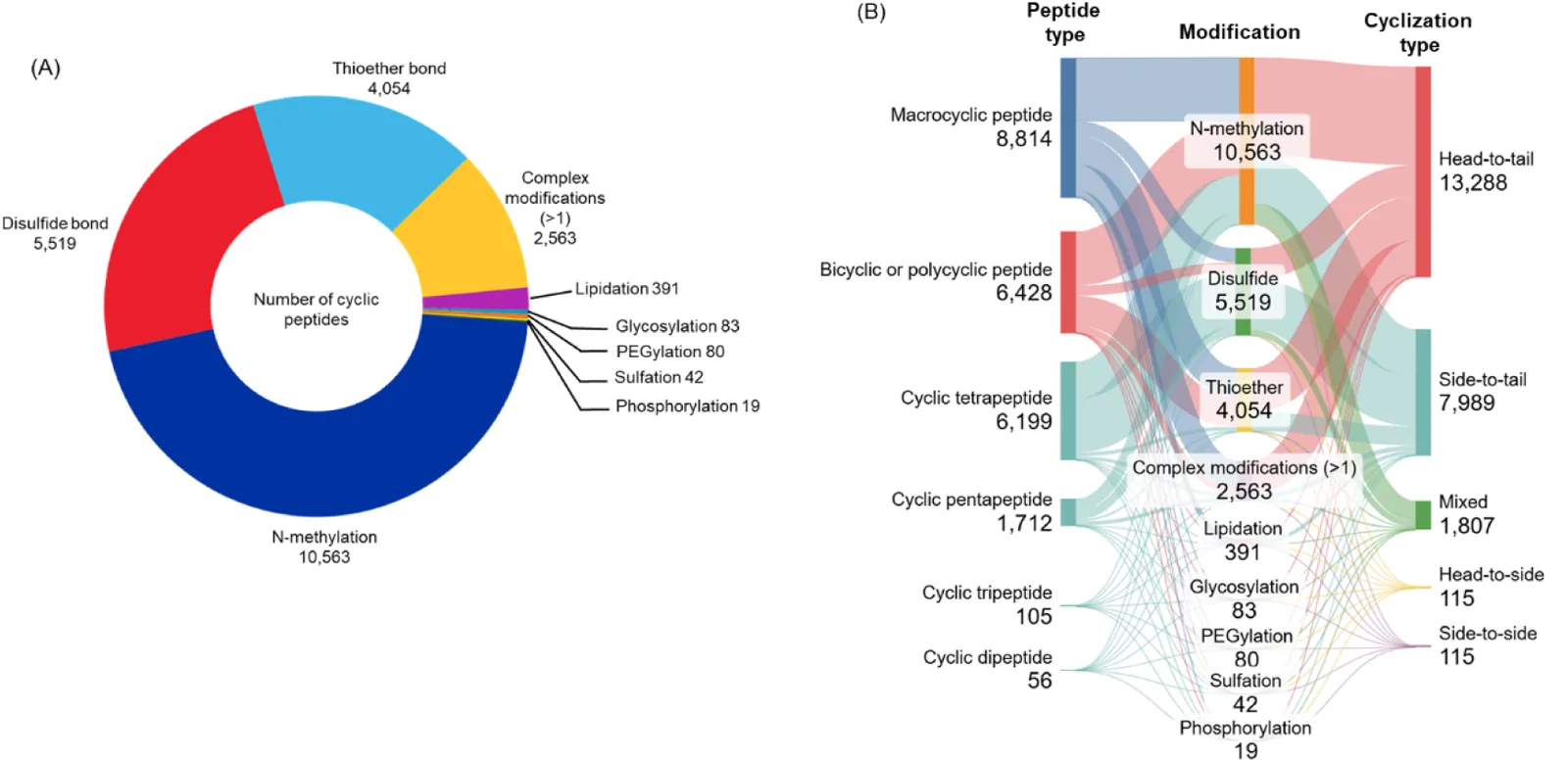
(A) Distribution of cyclic peptides with specified modifications. (B) Sankey graph showing co-occurrence between the various modifications, peptide type, and cyclization type. Only cyclic peptides indexed with CAS roles THU, or PAC, or PKT covering the period 2020–2025 were included for the analysis. Source: CAS Content Collection.

### 3.4 Physicochemical Landscape Analysis: Rational Design Principles for Cyclic Peptide Therapeutics

The comprehensive analysis of physicochemical properties provides crucial insights into the therapeutic potential and drug-like quality of cyclic peptides, informing rational selection strategies for experimental validation. We analyzed key physicochemical properties relevant to peptide drug-likeness, including hydrogen-bond donors and acceptors, molecular flexibility, polar surface area, lipophilicity, and p*K*_a_ distributions. Physicochemical property values were retrieved from CAS SciFinder, which employs ACD/Laboratories algorithms for property prediction (please see Methods section in Supporting Information for more details).

Figure 5A shows the percentage of cyclic peptides violating each individual criterion (Lipinski’s/Veber’s rules), revealing that a substantial fraction of the data set exceeds classical thresholds for MW, polar surface area, hydrogen-bond donors or acceptors, and rotatable bonds. This pattern reflects the inherent structural features of cyclic and macrocyclic peptides, which are larger and more polar than conventional small molecules. Figure 5B extends this analysis by depicting the percentage distribution of peptides according to the total number of rules violated simultaneously, demonstrating that most peptides breach multiple rules rather than failing a single parameter in isolation. Notably, peptides violating four or five rules constitute a major proportion of the data set, underscoring that therapeutic cyclic peptides predominantly reside in “beyond-Rule-of-5” chemical space. While this fact is well recognized in the literature,^68^ a systematic quantification across large data sets has been lacking. Here, we provide the first comprehensive analysis of Lipinski/Veber rule violations across 46,574 cyclic peptides.

**Figure 5:**
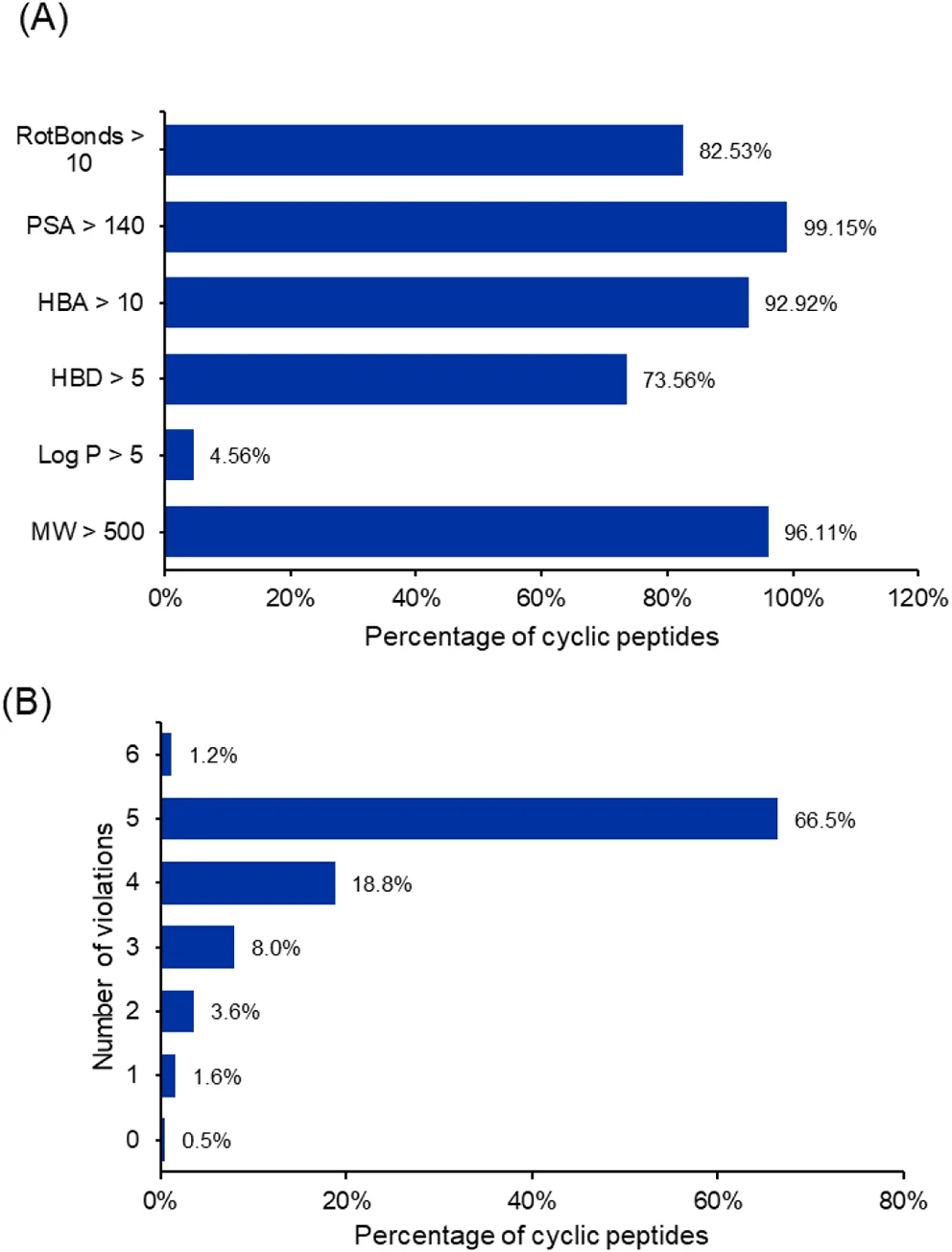
Percentage of cyclic peptides violating individual Lipinski’s and Veber’s drug-likeness criteria: (A) individual criteria and (B) number of rules violated. Source: CAS Content Collection.

Together, these percentage-based distributions highlight that classical drug-likeness rules systematically classify cyclic peptides as noncompliant, despite their known biological activity. This further emphasizes that alternative descriptors such as conformational rigidity, intramolecular hydrogen bonding, and three-dimensional shape are more appropriate for assessing the developability of peptide-based therapeutics.

The clustering observed across the physicochemical property distributions reflects a strong interplay between cyclic peptide size, conformational constraint, and therapeutic selection pressures inherent to the CAS-indexed data set (Figure 6A–E). Cyclization eliminates terminal functional groups and restricts backbone flexibility, which compresses properties such as hydrogen-bond donors, acceptors, and rotatable bonds into narrow, recurring ranges, particularly for macrocyclic peptides that dominate most bins. As peptide ring size increases, properties like polar surface area and hydrogen-bond acceptor count scale upward in a predictable manner due to additional amide bonds, yet these values remain clustered rather than broadly distributed because therapeutically relevant peptides are preferentially optimized to balance polarity, permeability, and binding affinity. Similarly, Log *P* values concentrate in the moderate range (0–5), reflecting medicinal chemistry bias against excessively hydrophobic or insoluble structures, while p*K*_a_ clustering around basic values arises from recurrent inclusion of cationic amino acids that enhance solubility and target interaction. Overall, the accumulation of specific peptide classes within discrete regions of each panel indicates that these compounds occupy a constrained and highly optimized chemical subspace, shaped not by random sequence diversity but by biological function, developability constraints, and the physicochemical requirements necessary for therapeutic activity in cyclic and macrocyclic peptides.

**Figure 6:**
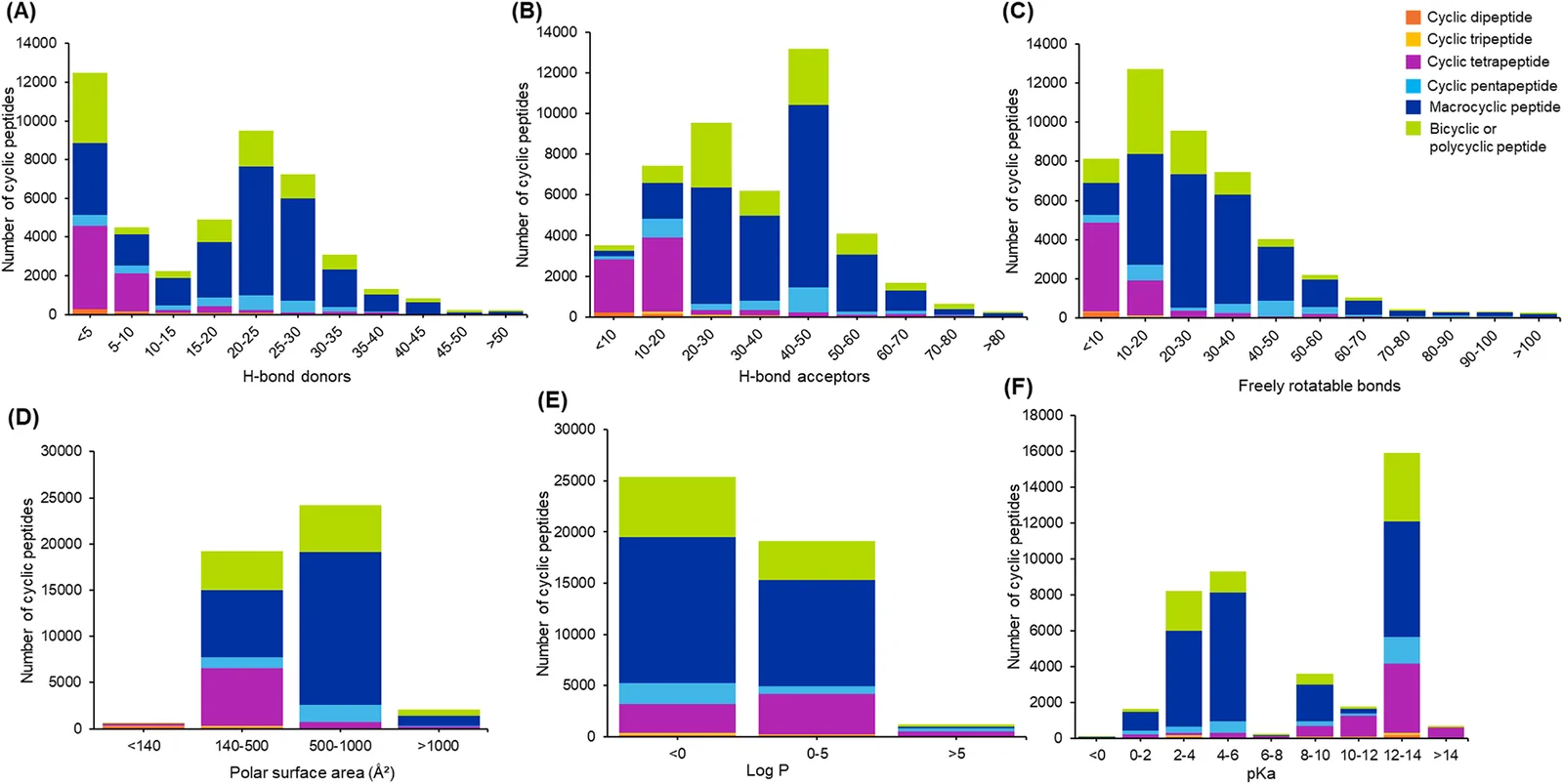
Distribution of key physicochemical properties of cyclic peptides: (A) hydrogen-bond donors, (B) hydrogen-bond acceptors, (C) rotatable bonds, (D) polar surface area, (E) Log *P*, and (F) p*K*_a_, shown as stacked bars colored by peptide type. Source: CAS Content Collection.

Figure 7 illustrates how the type of cyclization systematically likely influences the physicochemical property distributions of therapeutically annotated cyclic peptides. Across all panels, head-to-tail cyclization dominates the primary clusters for hydrogen-bond donors and acceptors, reflecting its prevalence in stabilizing peptide backbones while minimizing terminal polarity through amide closure. The clustering of freely rotatable bonds between ∼10 and 30 indicates that, despite differences in cyclization topology, most cyclic peptides converge toward a similar balance of rigidity and flexibility, with side-to-side and mixed cyclizations slightly shifting distributions toward higher flexibility due to additional linkers. Polar surface area shows pronounced accumulation in the 500–1000 Å^2^ range regardless of linkage type, underscoring that overall polarity is primarily dictated by peptide size and backbone composition rather than the specific cyclization chemistry, although side-chain cyclizations marginally populate the >1000 Å^2^ bin. Likewise, Log *P* values cluster predominantly between 0 and 5 across all cyclization modes, revealing a shared medicinal chemistry bias toward moderate lipophilicity, while basic p*K*_a_ values are enriched in mixed and side-chain cyclized peptides due to the frequent presence of ionizable side chains. Collectively, the observed clustering indicates that while cyclization topology introduces nuanced shifts in physicochemical profiles, therapeutically relevant cyclic peptides occupy a constrained and convergent chemical space shaped by developability and biological function rather than by cyclization mode alone. Together, our analysis shows that therapeutically relevant cyclic peptides, regardless of size or cyclization topology converge into a narrowly defined physicochemical space characterized by constrained hydrogen-bonding capacity, controlled flexibility, high but functionally masked polarity, and moderate lipophilicity, reflecting strong medicinal chemistry selection pressures rather than random structural diversity.

**Figure 7:**
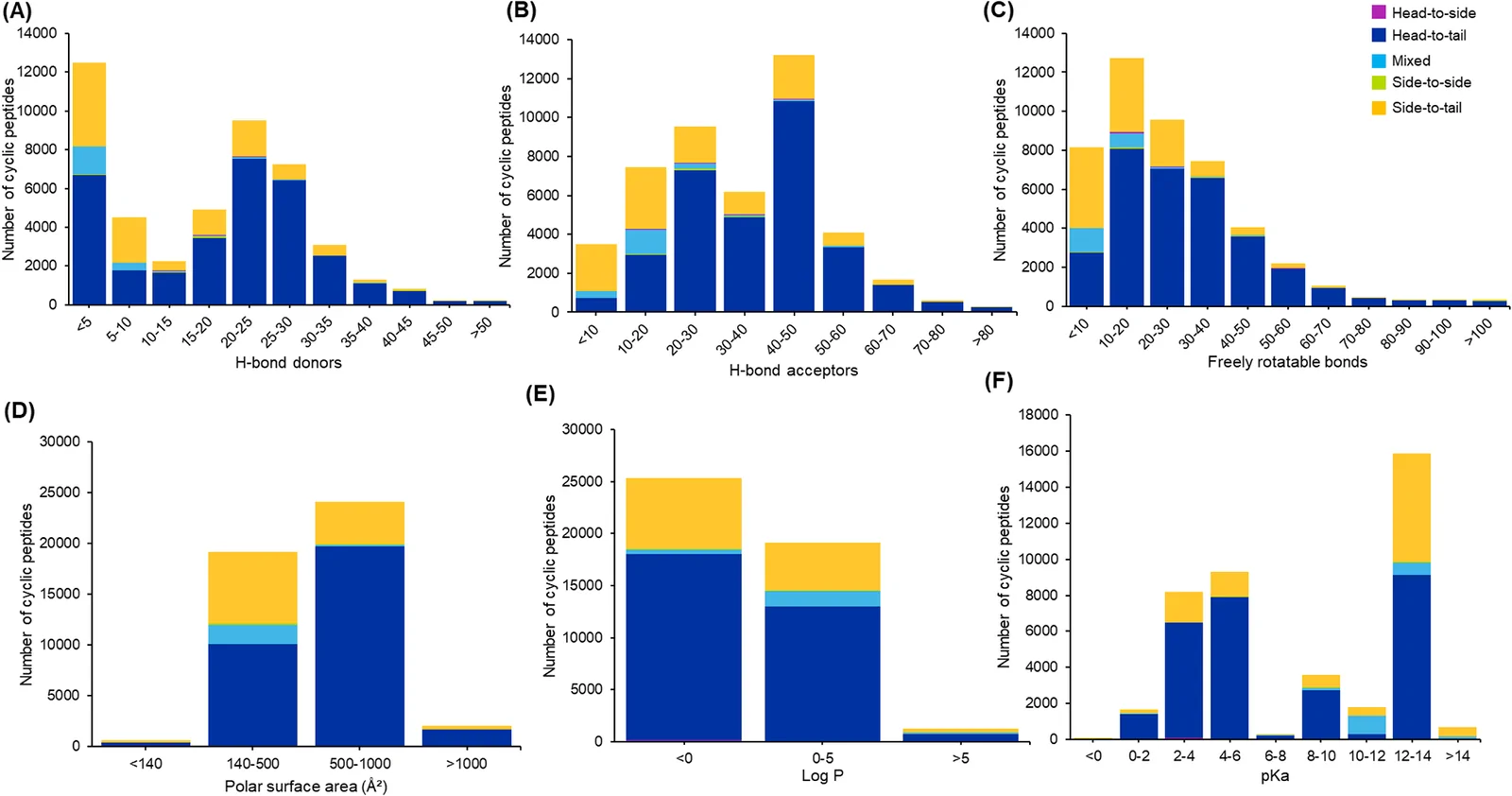
Distribution of key physicochemical properties of cyclic peptides: (A) hydrogen-bond donors, (B) hydrogen-bond acceptors, (C) rotatable bonds, (D) polar surface area, (E) Log *P*, and (F) p*K*_a_, shown as stacked bars colored by cyclization type. Source: CAS Content Collection.

MW vs Log *P* and MW vs polar surface area (PSA) scatter plots provide complementary perspectives for assessing cyclic peptide drug-likeness and permeability. The MW–Log *P* plot captures the balance between hydrophobicity and aqueous solubility, offering insight into the permeability–solubility trade-off relevant to peptide optimization, rather than strict compliance with small-molecule drug-likeness rules. In parallel, the MW–PSA plot provides mechanistic context by reflecting hydrogen-bonding capacity and overall polarity: higher PSA values generally indicate increased polarity that can limit passive membrane permeation, whereas moderate PSA, in combination with conformational flexibility, may enable chameleonic behavior through intramolecular hydrogen bonding. Taken together, these analyses support rapid visual filtering of peptide libraries, with MW–Log *P* highlighting compounds with balanced physicochemical profiles and MW–PSA explaining how structural features influence permeability, thereby guiding medicinal chemistry efforts toward more permeable, bioavailable cyclic peptide therapeutics.

The overlay (Figure 8A) and individual MW–PSA scatter plots (Supplementary Figure S8) show that therapeutically relevant cyclic peptides are not randomly distributed but instead occupy a narrow and constrained physicochemical space. Beyond the expected positive relationship between MW and polar surface area, the tight, elongated distribution indicates that polarity increases with size in a highly controlled way, suggesting that added mass arises mainly from similar, amide-rich structural features rather than diverse chemical functionalities. This consistent PSA–MW relationship points to optimization for partly shielded or “hidden” polarity, where high calculated PSA can be accommodated through conformational folding and intramolecular hydrogen bonding, particularly in larger macrocycles. Although different cyclization topologies (head-to-tail, side-to-tail, and mixed) vary in density and spread, they all follow the same overall scaling trend, indicating that cyclization influences the extent of polarity variation without altering the fundamental physicochemical regime. Importantly, approved and clinically advanced peptides fall within the same MW–PSA range as the broader data set, indicating that clinical success is achieved by maintaining this optimized balance rather than avoiding high MW or polarity. The lack of sparsely populated or clearly excluded regions further suggests selection against unfavorable property combinations, reflecting biological and medicinal-chemistry constraints. The extreme high-MW (>2500 Da) and high-PSA (>1400 Å^2^) outliers are largely older cyclic peptide drugs approved before 2010, consistent with discovery-driven development rather than deliberate property optimization.

**Figure 8:**
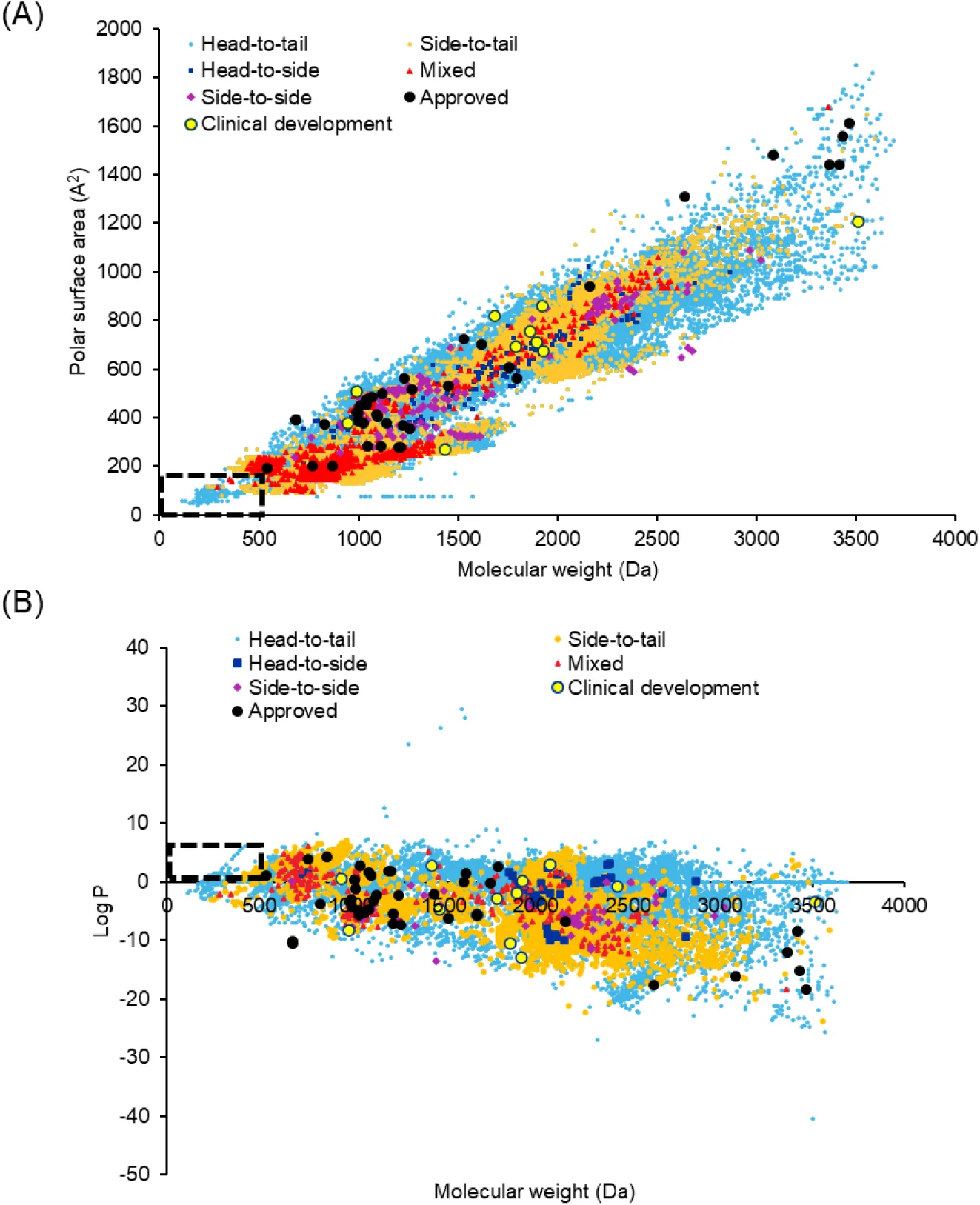
Scatter plots showing the relationship between (A) MW and polar surface area for cyclic peptides and (B) MW and Log *P*, stratified by cyclization types (colored symbols). Dashed black boxes indicate “ideal druggable properties” according to Lipinski’s and Veber’s rules. Source: CAS Content Collection.

The Log *P*–MW scatter plots (Figures 8B and S9) reveal that, unlike polar surface area, lipophilicity in cyclic peptides does not scale linearly with increasing MW but instead remains confined within a relatively narrow band across a wide MW range. This constrained distribution suggests that additional mass in larger cyclic and macrocyclic peptides is not accompanied by proportional increases in hydrophobic surface, indicating deliberate balancing between hydrophobic and polar residues during peptide design. The broad horizontal spread at intermediate MWs implies that peptides of similar size can achieve markedly different lipophilicity through sequence composition and cyclization strategy, highlighting Log *P* as a tunable rather than size-dictated property. When stratified by cyclization topology, head-to-tail peptides display the tightest clustering around moderate Log *P* values, reflecting backbone-dominated chemistry with limited hydrophobic diversification, whereas mixed and side-chain-cyclized peptides show greater variance and a tendency toward lower Log *P* at higher MWs, consistent with increased polar side-chain involvement. Importantly, approved and clinically developed peptides are distributed throughout the central Log *P* band rather than occupying extreme values, indicating that clinical success does not demand high lipophilicity even at elevated MWs. The absence of peptides with strongly increasing Log *P* at higher MWs further suggests negative selection against overly hydrophobic macrocycles, likely due to aggregation, poor solubility, or unfavorable pharmacokinetics. Overall, these plots demonstrate that therapeutic cyclic peptides achieve developability not through increasing hydrophobicity with size, but through maintaining lipophilicity within a constrained window while exploiting conformational control and intramolecular interactions to offset their large MW.

### 3.5 Co-Occurrence Patterns between Cyclic Peptide Features and Therapeutic Context

We further explored the co-occurrences between various peptide types and routes of administration, major therapeutic areas, and potential molecular targets. Figure 9 reveals several trends in the probable relationship between peptide design features and preferred administration routes.

- Oral, rectal, and intravenous (I.V.) delivery routes dominate almost all peptide classes, indicating that these routes remain the most feasible for diverse peptide structures. Macrocyclic, bicyclic, and polycyclic peptides are heavily represented in oral and I.V. routes, consistent with their enhanced metabolic stability and structural rigidity.^37,69^

- Subcutaneous (S.C.) and transdermal routes had very few cyclic peptides associated with them in our data set. Macrocyclic and polycyclic peptides are less dominant in the case of intramuscular and nasal routes. Their large size, rigidity, limited aqueous solubility, and poor absorption make them poorly suited for intramuscular and nasal routes, which rely on fast absorption of drugs in systemic circulation.^70,71^

- Some of the intermediate-sized cyclic peptides (like tetra- and pentapeptides) are being explored for intramuscular and nasal routes.^72,73^

- The topical route appears to have been explored mostly for bacterial skin infections, and inflammatory skin conditions like atopic dermatitis.
- Among cyclization types, head-to-tail, side-to-tail, and mixed types show maximum co-occurrence with oral, rectal, and I.V. routes, owing to their high stability and protease resistance.

**Figure 9:**
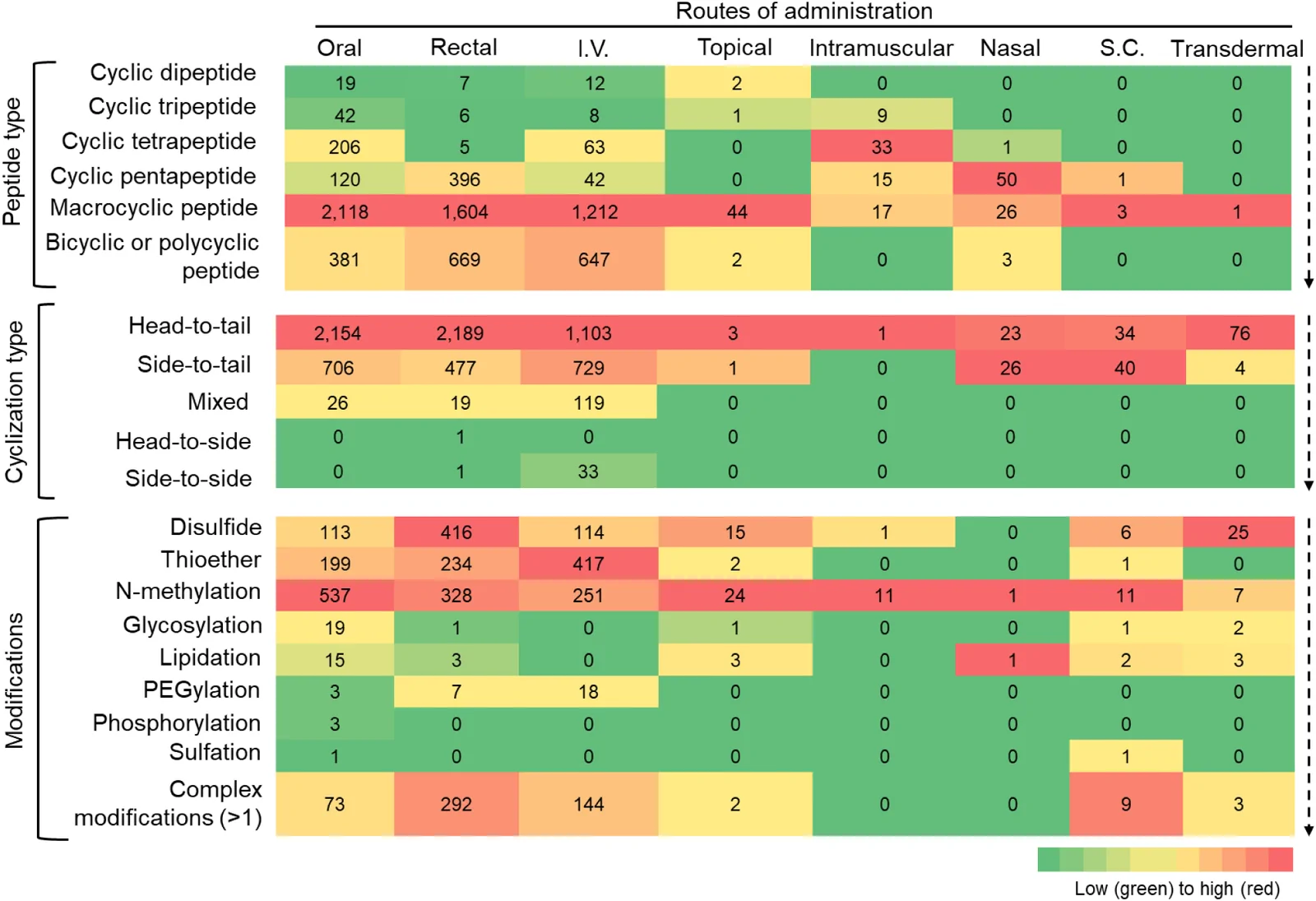
Heatmap showcasing the co-occurrence of cyclic peptide types, cyclization types, and modifications with various administration routes. The heatmap is to be read vertically, with color intensity indicating relative frequency of number of cyclic peptides (green = low, red = high). Only cyclic peptides indexed with CAS roles THU, or PAC, or PKT covering the period 2020–2025 were included for the analysis. Source: CAS Content Collection.

A closer examination of the modification patterns highlights how different chemical strategies influence compatibility with certain routes of administration (Figure 9).

- *N*-methylation shows one of the strongest co-occurrence across oral, rectal, and I.V. routes, underscoring its well-known role in increasing protease resistance and improving membrane permeability by reducing hydrogen-bond donors.^55^
- Disulfide and thioether linkages also appear prominently across major routes, reflecting their widespread use in stabilizing peptide secondary structures and constraining conformations to enhance metabolic stability.^74,75^

- Glycosylation and lipidation show distinct distribution, co-occurring mostly with oral route.
- PEGylation appears to be a modification mostly co-occurring with I.V. formulations, improving their half-life.^76^
- The category of complex modifications (more than one modification) shows notable co-occurrence with the major routes, reflecting how drug discovery strategies are employing combinatorial chemical modifications to simultaneously address the multifactorial barriers associated with these routes.

Next, we looked at co-occurrences of cyclic peptides with several diseases (Figure 10) and observed a strong prominence of macrocyclic peptides across nearly all therapeutic areas, with an especially high co-occurrence with cancer, infectious, inflammatory, and autoimmune diseases. This trend underscores the structural advantage of macrocycles—enhanced stability, surface area for target engagement, and the ability to modulate traditionally undruggable targets—which align well with the mechanistic challenges in these diseases.^77,78^ Apart from macrocyclic peptides, bicyclic and polycyclic peptides also show relatively high co-occurrence across all therapeutic areas. Cyclic pentapeptides and tetrapeptides show notable co-occurrences, although to a much lesser degree, suggesting that while these scaffolds are valued for their rigidity and selectivity, they have narrower applicability or face greater synthetic constraints.^79^ Our analysis highlights a few specific hotspots like in autoimmune diseases cyclic pentapeptides, macrocyclic, and bi- or polycyclic peptides appear to be widely explored.

**Figure 10:**
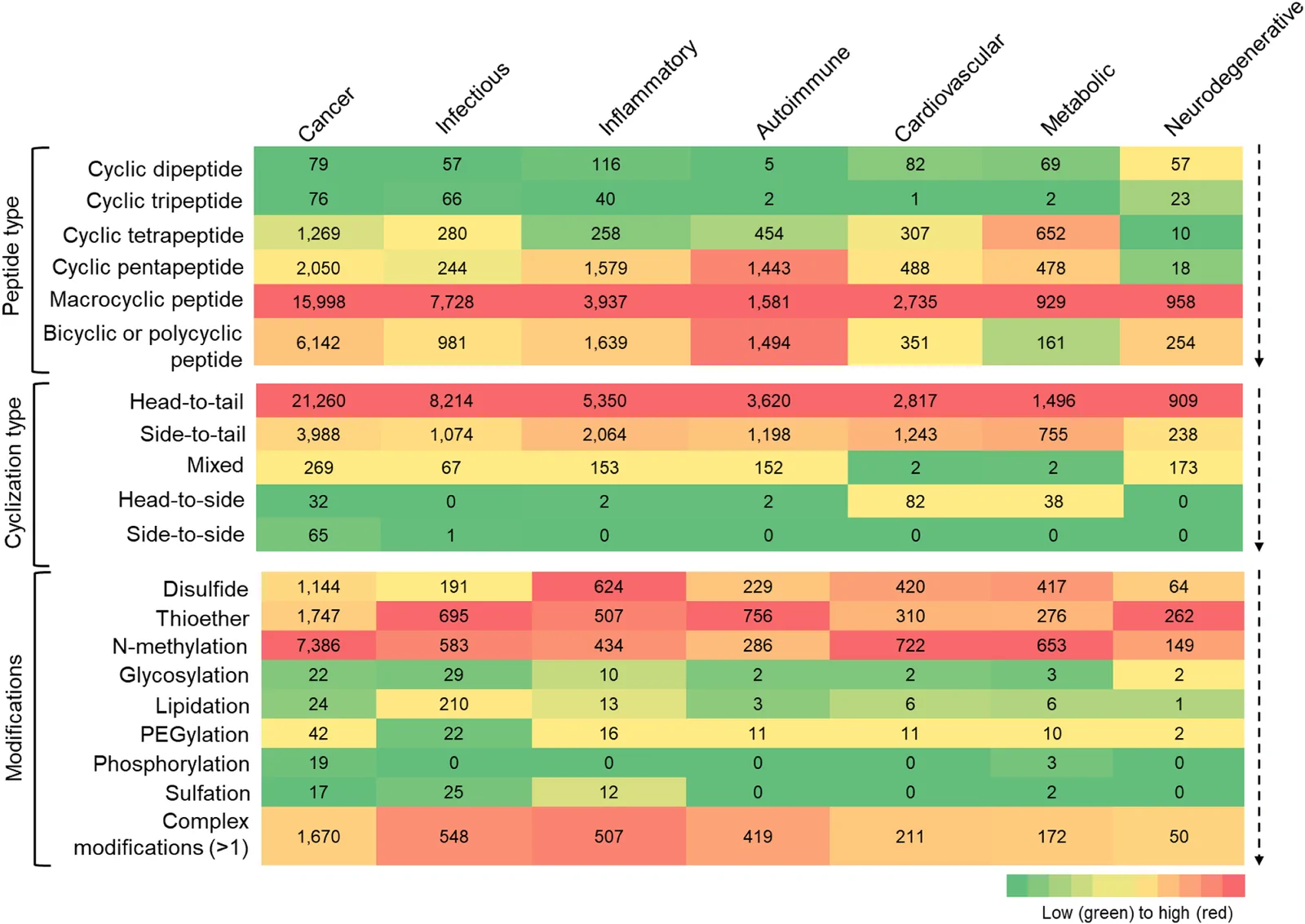
Heatmap showcasing the co-occurrence of cyclic peptide types, cyclization types, and modifications with various therapeutic areas. The heatmap is to be read vertically, with color intensity indicating relative frequency of number of cyclic peptides (green = low, red = high). Only cyclic peptides indexed with CAS roles THU, or PAC, or PKT covering the period 2020–2025 were included for the analysis. Source: CAS Content Collection.

Co-occurrence between cyclization patterns and therapeutic areas (Figure 10) highlights the well-known preference for head-to-tail cyclized peptides due to their reliability in imparting conformational restraint and protease resistance without introducing complex chemistries. Among modifications, disulfide and thioether linkages, *N*-methylation, and complex modifications show greater co-occurrences across all therapeutic areas. The relative co-occurrences of other modifications suggest that their use is selective rather than having broad applicability.

We leveraged our expertly curated CAS concept data to identify proteins co-occurring frequently with our identified cyclic peptides to understand trends with respect to different peptide types, cyclization strategies, and chemical modifications. These ten potential molecular targets (shown in Figure 11) represent a diverse collection spanning multiple cellular compartments and functions, yet they share common characteristics that make them attractive for cyclic peptide therapeutics. The targets include cell surface immune regulators (PD1/PDL1/PDL2), intracellular signaling proteins (Ras, SOS1, STAT3), nuclear factors (TP53, MDM family), secreted enzymes (Factor IIa), inflammatory mediators (TNF superfamily), and metabolic receptors (MCR family), with most having significant implications in cancer, autoimmune diseases, or metabolic disorders. While traditional druggability varies widely, from highly druggable targets like Factor IIa and TNF superfamily to historically “undruggable” proteins like Ras and TP53, all identified proteins/molecules represent viable cyclic peptide targets due to their involvement in protein–protein interactions (PPIs), large binding interfaces, or natural peptide ligand recognition. Their frequent appearance in cyclic peptide literature reflects both high therapeutic importance and the unique advantages cyclic peptides offer: larger binding surfaces for shallow PPI interfaces, enhanced selectivity through conformational constraint, tunable membrane permeability for intracellular targets, and the ability to access binding sites unavailable to traditional small molecules, making them particularly valuable for addressing previously intractable therapeutic targets. While PPIs represent a major focus of cyclic peptide therapeutic development, cyclic peptides have also been explored against other target classes, including enzymes and transporters. Enzymatic targets such as proteases and kinases can benefit from the conformational rigidity and extended interaction surfaces provided by cyclic peptides, enabling high affinity and selectivity, particularly for substrate-recognition or allosteric sites that are challenging for small molecules. Cyclic peptides have also been investigated against transporter and membrane-associated targets, where enhanced stability and constrained conformations may support extracellular or luminal engagement. The comparatively lower representation of enzymatic and transporter targets within the present data set likely reflects historical development priorities and platform-specific strategies, rather than intrinsic limitations of cyclic peptides for these target classes. As design strategies, delivery approaches, and permeability-enabling modifications continue to advance, these target classes are expected to represent expanding areas of cyclic peptide therapeutic exploration. The heatmap (Figure 11) shows clear patterns: macrocyclic peptides appear to co-occur with several molecular targets, particularly those associated with PD-1/PD-L1, Ras family, and SOS1. This indicates a strong preference for macrocyclic scaffolds in peptide libraries, likely due to their conformational rigidity and stability. Head-to-tail cyclization is emerging as the dominant strategy. Side-to-tail and mixed cyclization approaches appear more often with proteins such as MDM, p53, and Factor IIa, while chemical modifications like *N*-methylation are frequently associated with Ras proteins. Disulfide bridges and multimodifications occur broadly, reflecting design practices aimed at improving stability and permeability. It should be noted that these patterns represent descriptive co-occurrences within the data set and do not distinguish whether the observed preferences reflect intrinsic target-specific requirements or are influenced by historical development trajectories, platform technologies, or company-specific design strategies.

**Figure 11:**
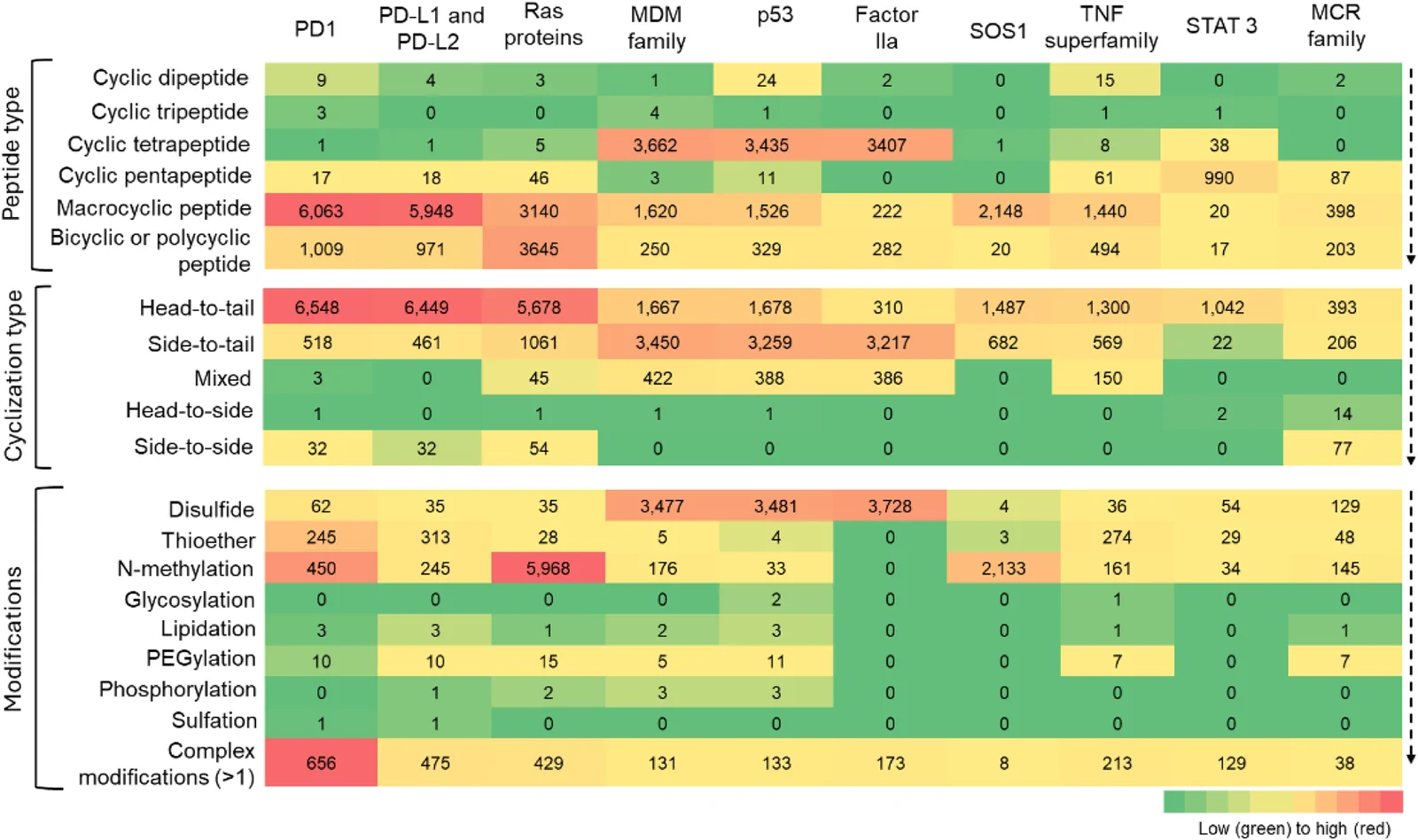
Heatmap summarizing the co-occurrence of cyclic peptide types, cyclization types, and modifications across various potential molecular targets. The heatmap is to be read vertically, with color intensity indicating relative frequency of number of cyclic peptides (green = low, red = high). Only cyclic peptides indexed with CAS roles THU, or PAC, or PKT covering the period 2020–2025 were included for the analysis. Source: CAS Content Collection.

The preceding analysis underscores the structural and functional diversity of cyclic peptides indexed in the CAS Content Collection, highlighting the interplay between cyclization strategies, chemical modifications, therapeutic indications, and molecular targets. While these findings illustrate the breadth of current research and development, they also emphasize a persistent limitation in the clinical translation of cyclic peptides: their restricted oral bioavailability. Recent advances in molecular design and formulation approaches have begun to address this challenge, positioning orally available cyclic peptides as an emerging and strategically important subdomain. The following section therefore examines this evolving area in greater detail, with particular attention to design principles and therapeutic implications.

## 4 Orally Bioavailable Cyclic Peptides

Traditional biologics such as monoclonal antibodies and recombinant proteins have revolutionized the treatment of diseases ranging from cancer to autoimmune disorders. Their ability to engage PPIs with high specificity has enabled the targeting of previously “undruggable” pathways.^80^ However, these macromolecules are limited by their need for parenteral administration, which reduces patient compliance, increases healthcare costs, and complicates treatment logistics, especially for chronic conditions requiring frequent dosing. Conversely, small molecules offer oral bioavailability and ease of administration but often lack the surface area and conformational flexibility needed to effectively modulate PPIs. This dichotomy has created a therapeutic void: targets that demand the precision of biologics, but the delivery simplicity of oral drugs remain largely inaccessible. Cyclic peptides, with their intermediate size and tunable properties, are uniquely positioned to fill this gap.

Our analysis of the cyclic peptides data set revealed distinct trends in the exploration of different drug administration routes across literature. Overall, the oral route dominated the field, accounting for the largest proportion of publications (Figure 12A) and showing a sharp and sustained rise in interest over the past decade (Figure 12B). This trend reflects the growing emphasis on improving oral bioavailability and stability of cyclic peptides, an area historically considered challenging due to their size and polarity. In comparison, other routes—including rectal, I.V., S.C., transdermal, topical, intramuscular, and nasal—appear far less represented with relatively modest and stable publication numbers over time.

**Figure 12:**
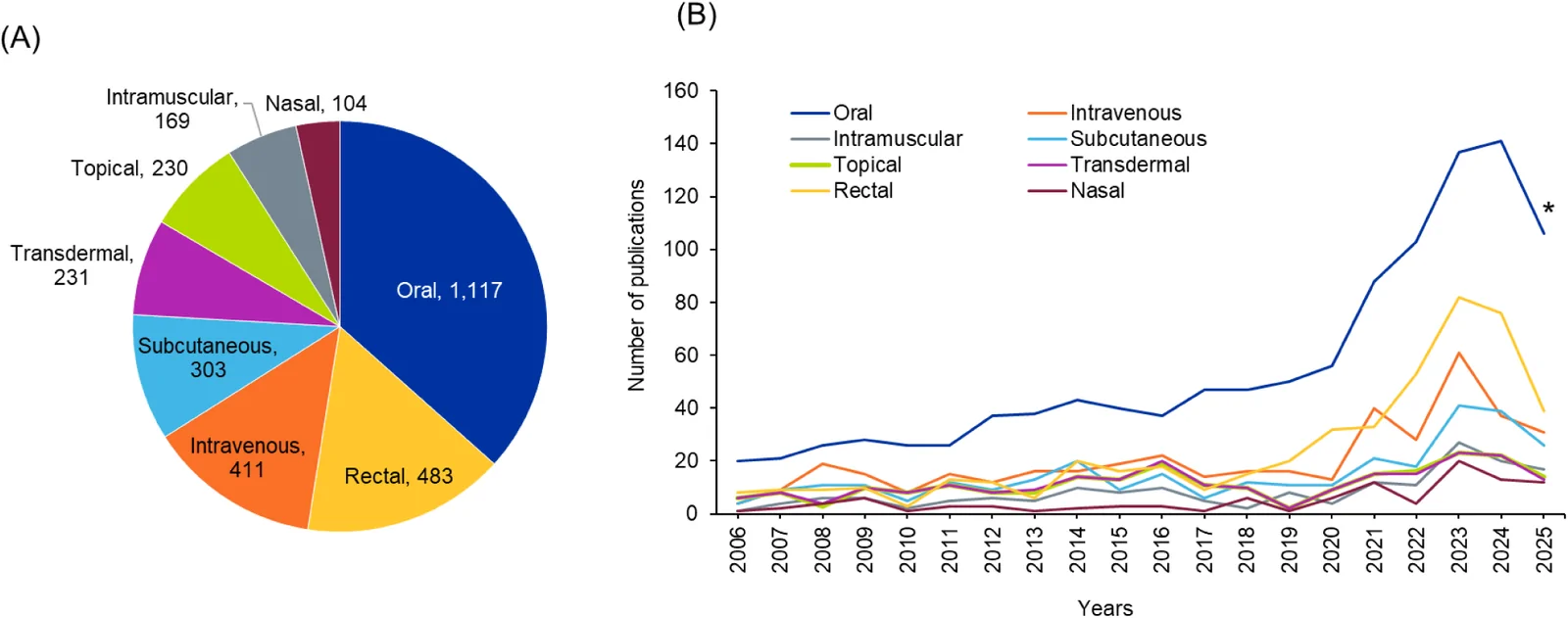
(A) Distribution of publications (journals and patents) and (B) their year-wise trends related to cyclic peptides based on the route of administration. Source: CAS Content Collection for the period 2006–2025. *Data until August 2025.

### 4.1 Key Challenges in Oral Delivery of Cyclic Peptides

The poor oral bioavailability of cyclic peptides arising from a constellation of interdependent factors, including physicochemical constraints, gastrointestinal (GI) tract biology, enzymatic degradation, active efflux, and first-pass metabolism, which collectively result in low and variable oral absorption, remains a significant obstacle. In this section, we briefly discuss the challenges associated with the oral delivery of cyclic peptides and highlight ongoing strategies to overcome these barriers.

#### 4.1.1 Physicochemical Limitations

Most cyclic peptides possess high MWs (typically >500 Da), large polar surface areas, and numerous hydrogen-bond donors and acceptors, which violate conventional oral drug design rules such as Lipinski’s Rule of Five^81^ and Veber’s criteria.^82^ These properties limit passive membrane permeation, a key requirement for oral absorption. While cyclization can reduce conformational flexibility and mask polar groups through intramolecular hydrogen bonding, improving proteolytic stability and sometimes permeability, however, residual polarity, size, and excessive rigidity still hinder both transcellular diffusion and paracellular passage. Achieving a balance between aqueous solubility (for dissolution in GI fluids) and lipophilicity (for membrane partitioning) remains a major formulation challenge. These physicochemical barriers are fundamental drivers of low permeability observed in in vitro models like Caco-2 cells and in vivo studies.^83^

#### 4.1.2 Enzymatic Degradation and Chemical Instability

The alimentary canal and the brush border present a rich complement of proteases (pepsin, trypsin, chymotrypsin, brush-border peptidases) and acidic/alkaline microenvironments that cleave peptide bonds or modify labile side chains. While cyclization frequently increases protease resistance relative to linear peptides, many cyclic scaffolds still present susceptible linkages or solvent-exposed residues that enzymes can attack.^84^ Moreover, pH-dependent chemical reactions (deamidation, epimerization) can further reduce the fraction of intact drug reaching absorptive surfaces.^85^

#### 4.1.3 Mucus Barrier and Epithelial Architecture

The mucus gel overlaying the epithelium acts as a barrier by trapping or slowing the diffusion of molecules that interact with mucin, especially large, charged, or hydrophobic peptides, thereby reducing their effective concentration at the epithelial surface. Studies show that cyclic peptides like cyclosporin A can aggregate gel-forming mucins (MUC2, MUC5AC, MUC5B), further impeding their diffusion.^86^ Beyond the mucus layer, tight junctions restrict paracellular transport to very small solutes (typically <500 Da), effectively excluding most cyclic peptides. These junctions form a selectively permeable barrier that blocks macromolecules and peptides from passing between epithelial cells.^87^ Dedicated uptake transporters for large cyclic scaffolds are rare. As a result, cyclic peptides rely on transcellular passive diffusion or receptor-/lectin-mediated endocytosis, which are often inefficient.

#### 4.1.4 Active Efflux and First-Pass Metabolism

Many peptide and macrocyclic scaffolds are substrates for efflux pumps such as P-glycoprotein (P-gp) and multidrug resistance proteins (MRPs), which actively transport them back into the intestinal lumen, thereby reducing net uptake and limiting oral bioavailability.^88^ Even when peptides are taken up into enterocytes, they may undergo degradation by intracellular and hepatic enzymes, resulting in substantial first-pass metabolic loss and contributing to interindividual variability in systemic exposure.^89,90^ Cyclosporine A, a classic example of an orally available cyclic peptide, illustrates how formulation strategies (e.g., microemulsions, liposomes, cyclodextrin complexes) and interactions with transporters like P-gp can lead to a wide range of bioavailability across different patient populations and product types.^91,92^

#### 4.1.5 Pharmacokinetic Variability and Clinical Implications

The combined effect of dissolution variability, GI transit, fed/fasted state, microbiome interactions, and transporter/enzyme expression results in erratic PK profiles for many orally dosed cyclic peptides.^93^ Low absolute bioavailability often necessitates large oral doses or parenteral alternatives, increasing cost and reducing patient convenience.

### 4.2 Strategies to Overcome Challenges in Oral Delivery

To translate cyclic peptides into practical oral therapeutics, contemporary strategies operate at three complementary levels: (1) molecular engineering to improve intrinsic permeability and stability, (2) formulation and excipient strategies to protect and present peptides to absorption sites, and (3) device- and platform-level innovations that bypass or actively traverse physiological barriers. Integration across levels, “molecule + formulation + device”, increasingly defines successful programs.

#### 4.2.1 Molecular and Chemical Design

##### 4.2.1.1 Backbone Modifications and Noncanonical Residues

Chemical modifications such as *N*-methylation, incorporation of d-amino acids, β-amino acids, and peptidomimetic linkers (e.g., azapeptides, amide isosteres) reduce hydrogen bond donors and proteolytic susceptibility, enhancing membrane permeability and metabolic stability. For example, incorporating d-amino acids into somatostatin analogs has increased stability and prolonged half-life.^94^ *N*-methylation has been used to develop peptide drugs like d-amino-8-d-arginine vasopressin (DDAVP, a synthetic form of vasopressin), which exhibits improved stability and antidiuretic activity.^95^ Combining *N*-methylation with head-to-tail cyclization has yielded peptides with improved oral absorption profiles.^55^ Our analysis shows a high co-occurrence between these two and the oral route of administration (Figure 9).

##### 4.2.1.2 Lipidation and Prodrugs

Lipidation, the chemical attachment of acyl or alkyl lipid chains to peptides, enhances amphipathicity and is widely employed to improve pharmacokinetic and pharmacodynamic properties. A recent study demonstrated the design and synthesis of cyclic lipidated peptides derived from the C-terminal region of Cx43, aimed at inhibiting hemichannel activity and targeting cardiac endothelium.^96^ Our analysis indicates lipidation is relatively less explored overall as compared to other modifications (Figure 4A) and even more so in the context of oral cyclic peptides (Figure 9).

The prodrug strategy involves chemically modifying peptides into inactive forms that are converted back into active drugs postabsorption. This approach improves key properties such as lipophilicity, membrane permeability, and metabolic stability. Common modifications include masking polar groups through esterification, altering amide bonds to evade enzymatic degradation, and attaching cleavable pro-moieties that aid transport and are activated enzymatically or chemically after uptake.^97^

##### 4.2.1.3 Chameleonic Design and Intramolecular H-Boning

As part of broader molecular strategies to enhance the oral bioavailability of cyclic peptides, chameleonic design addresses the challenge of balancing aqueous solubility with membrane permeability. Chameleonic cyclic peptides adopt environment-dependent conformations, exposing polar groups in aqueous media while reorganizing in low-polarity environments, such as lipid membranes, to form intramolecular hydrogen bonds (IMHBs) that transiently mask polar surface area and facilitate passive diffusion.^98,99^ In practice, this behavior is pursued through sequence- and backbone-level modifications that complement other approaches, including selective backbone *N*-methylation, strategic placement of hydrogen bond donors and acceptors, and incorporation of noncanonical or sterically tuned residues to shift conformational equilibria without excessive rigidification.^100^ These changes are typically introduced iteratively to preserve solubility, target affinity, and metabolic stability. Chameleonic design is commonly implemented using a semi rational workflow combining targeted structural modification with conformational analysis by molecular dynamics simulations and solvent dependent NMR studies.^101^ As predictive design rules are still evolving, chameleonicity is best viewed as a complementary and emerging strategy for improving cyclic peptide oral bioavailability.

##### 4.2.1.4 AI and Computational Prediction

Recent machine-learning tools specifically trained to predict cyclic peptide membrane permeability and chameleonic behavior (e.g., CycPeptMP^102^ and other AI models) accelerate the identification of sequences with favorable oral absorption properties, enabling library-scale preselection prior to synthesis. These computational advances are rapidly shortening discovery timelines and focusing medicinal chemistry efforts.^103^

#### 4.2.2 Formulation and Excipient Strategies

##### 4.2.2.1 Lipid-Based Systems

Self-emulsifying drug delivery systems (SEDDS) are isotropic mixtures of oil, surfactant/cosurfactant and solvent/cosolvent that spontaneously emulsify when diluted in aqueous fluids.^104^ Their renewed potential for oral peptides has emerged from a set of recent studies demonstrating that peptides could dissolve in the oil phase using the principle of hydrophobic ion pairing (HIP) through which peptide lipophilicity could be increased.^105^ Lipid-based nanoparticles, including liposomes, solid lipid nanoparticles (SLNs), and nanostructured lipid carriers (NLCs),^106^ offer another approach to peptide delivery.^107^ They improve solubilization, stabilize apolar cyclic peptides, and promote lymphatic transport, proven in the commercialization of cyclosporine (Neoral) and continually applied to newer macrocycles.^108^ The relative distribution of documents associated with various delivery systems for cyclic peptides is summarized in Supplementary Figure S10.

##### 4.2.2.2 Polymeric Nanoparticles and Mucoadhesives

Polymeric nanoparticles,^109^ commonly made from biodegradable and biocompatible polymers such as poly­(lactic-*co*-glycolic acid) (PLGA), polylactic acid (PLA), chitosan, polyhydroxyalkanoates (PHAs), and thiolated polymer systems, can encapsulate peptides within their matrix or adsorb them onto their surface, shielding them from luminal enzymes, enabling controlled release, and increasing residence time via mucoadhesion.^110,111,112^ Chitosan derivatives can also transiently modulate tight junctions to enhance paracellular flux. Safety and reversibility of permeation modulation are critical concerns. The preactivated thiolated chitosan nanoparticles loaded with octreotide increased systemic exposure and sustained the hypoglycemic effect in rats.^113^

##### 4.2.2.3 Enteric and pH-Responsive Coatings

Enteric polymers shield peptides from gastric acid and release them in the intestine where absorption potential is greater. Smart polymers that release payloads in target intestinal segments further refine exposure windows and reduce premature degradation.^114^

##### 4.2.2.4 Coformulation with Protease Inhibitors and Permeation Enhancers

Coadministration of enzyme inhibitors is used to protect peptides from degradation in the GI tract by temporarily inhibiting digestive enzymes like trypsin and chymotrypsin. Agents such as aprotinin, soybean trypsin inhibitor, and bacitracin have shown enhanced peptide absorption in preclinical studies. However, concerns around safety, interference with normal digestion, and regulatory challenges limit their clinical application.^115^

Permeation enhancers temporarily increase intestinal epithelial permeability, enabling absorption of peptides with poor membrane permeability. They act by opening tight junctions (enhancing paracellular transport), disrupting cell membranes (facilitating transcellular uptake), and inhibiting efflux transporters to boost intracellular drug levels.^116,117,118^ A notable clinical example is MYCAPSSA (octreotide), approved by the U.S. FDA in June 2020.^119,120^ It uses Transient Permeation Enhancer (TPE) technology, an enteric-coated capsule containing an oily suspension of octreotide and sodium caprylate, which transiently opens tight junctions to improve absorption.^121^

#### 4.2.3 Device- and Platform-Level Innovations

##### 4.2.3.1 Ingestible Microneedle/Biologic Devices

A new generation of ingestible devices is redefining oral delivery by physically bypassing traditional absorption barriers and enabling direct translocation of biologics across the GI mucosa. The self-orienting millimeter-scale applicator (SOMA) has demonstrated proof-of-concept for gastric mucosal injection of biologics, enabling systemic exposure without full-thickness penetration of the stomach wall.^122^ Related platforms, including Rani Therapeutics’ RaniPill and the intestinally deploying LUMI device, have advanced toward clinical or late preclinical evaluation, achieving bioavailability levels exceeding 10% in large animal models.^123,124^ Liquid-injecting SOMA (L-SOMA) and other self-unfolding proximity-enabling devices have further shown rapid systemic exposure for proteins and peptides such as insulin, with pharmacokinetics comparable to parenteral administration in swine models.^125,126^

To date, these platforms have been primarily evaluated using proteins or linear peptide therapeutics, rather than cyclic peptides specifically. However, their ability to deliver large, impermeable macromolecules without reliance on molecular permeation suggests potential applicability to cyclic peptides that remain refractory to chemical or formulation-based oral delivery approaches. While further validation is required to establish suitability, targeting precision, and long-term safety for cyclic peptide applications, ingestible device-based delivery systems represent a promising complementary avenue for addressing oral bioavailability limitations in this modality.

##### 4.2.3.2 High-Velocity and Convective Delivery Capsules

Emerging platforms that employ mechanical or fluidic forces to transiently penetrate or permeate intestinal tissue (e.g., high-velocity jet capsules, expanding structures) are under development and show promise to deliver intact peptide doses to the submucosa, reducing enzymatic exposure.^127,128^

##### 4.2.3.3 Transporter Exploitation and Biomimetic Conjugation

Conjugating peptides to molecular moieties recognized by endogenous intestinal transporters (bile acids, dipeptide motifs) can enable receptor- or carrier-mediated uptake. This strategy leverages host physiology for active uptake and is being actively explored in preclinical programs.^129^

Experience shows no single tactic suffices for all cyclic peptides. Rather, integrated solutions, combining rational sequence design (*N*-methylation, noncanonical residues), predictive AI selection, protective/targeted formulations (lipids, nanoparticles, enteric coatings), and, where necessary, device-based delivery, produce the best chance of attaining clinically meaningful oral bioavailability. Recent advances in computational models and proof-of-concept device successes suggest that peptides once considered strictly parenteral may now be amenable to oral dosing strategies. Continued emphasis on safety (especially for permeation enhancers and mechanical devices), manufacturability, and regulatory acceptability will be essential as multiple platforms progress from preclinical to clinical stages.

### 4.3 Clinically Successful Oral Cyclic Peptides: Why Do They Remain Rare?

Although cyclic peptides are increasingly explored as therapeutic modalities, clinically meaningful oral exposure remains rare. The most notable example, cyclosporine A, achieves modest and variable oral bioavailability through a unique combination of macrocyclization, conformational flexibility, and extensive intramolecular hydrogen bonding that masks polar functionality.^19^ Related analogues, such as voclosporin, further illustrate that only minor structural changes can significantly affect permeability and pharmacokinetics, underscoring the narrow design space for oral cyclic peptides.^130^

Currently, successful oral cyclic peptides represent rare, highly optimized outliers rather than the norm. These compounds typically exploit a combination of macrocyclization, extensive intramolecular hydrogen bonding, conformational flexibility, and carefully balanced lipophilicity to mask polarity during membrane transit while remaining sufficiently soluble in aqueous environments. In summary, oral cyclic peptides remain uncommon because clinical success requires an exceptional alignment of properties like effective polarity masking, adaptive conformational behavior, metabolic stability, and sufficient systemic exposure that only a very small fraction of cyclic peptides can achieve simultaneously. Despite limited clinical success so far, the strong research emphasis on oral delivery underscores ongoing efforts to address the challenges limiting oral cyclic peptides.

## 5 Clinical Landscape

Cyclic peptide therapeutics have demonstrated substantial clinical impact, with regulatory approvals spanning more than seven decades and addressing diverse therapeutic areas (Supplementary Table S4). The progression of cyclic peptide therapeutics reflects advances in drug development technologies and evolving clinical needs across diverse therapeutic areas. In this section, we will discuss successful cyclic peptide therapeutics that have received regulatory approval along with promising cyclic peptide therapeutic candidates that are currently in the clinical development pipeline. The initial wave of cyclic peptide approvals (1940s–1970s) established these molecules primarily as antibacterial agents, with bacitracin (1948), polymyxin B (1951), and vancomycin (1958) representing foundational therapeutics for serious bacterial infections. These early compounds were administered predominantly via intravenous or topical routes and frequently incorporated complex chemical modifications to optimize antimicrobial activity. For instance, vancomycin contains disaccharide modifications that contribute to its mechanism of bacterial cell wall synthesis inhibition, while polymyxin B features fatty acid chains that facilitate membrane disruption. This era established cyclic peptides as clinically viable therapeutic agents, despite their structural complexity and parenteral administration requirements.

Beginning in the 1980s, the therapeutic scope of cyclic peptides expanded dramatically beyond anti-infective applications. This period witnessed approvals targeting endocrine pathologies (octreotide for acromegaly, 1988), cardiovascular emergencies (nesiritide for acute heart failure, 2001), and immune-mediated disorders (voclosporin for lupus nephritis, 2021). This diversification reflects advances in peptide engineering, target identification, and understanding of complex disease mechanisms. The development of somatostatin analogs exemplifies this evolution, with octreotide, lanreotide, and pasireotide demonstrating refined receptor selectivity profiles and improved pharmacokinetic properties across successive generations.

Recent approvals (2020–2023) underscore the continued innovation within this therapeutic class, addressing previously intractable clinical challenges. Setmelanotide (2020) targets melanocortin-4 receptor pathways in genetic obesity disorders, representing a precision medicine approach to metabolic disease. Zilucoplan (2023) inhibits complement C5 for generalized myasthenia gravis, while rezafungin (2023) provides extended-spectrum antifungal activity against invasive candidiasis. These contemporary therapeutics reflect sophisticated target selection, advanced peptide engineering strategies, and comprehensive understanding of pharmacology-disease relationships.

The approved cyclic peptide landscape demonstrates remarkable molecular diversity, with MW ranging from approximately 540 g/mol (romidepsin) to over 43,000 g/mol (pegcetacoplan). This 80-fold range reflects transformative advances in bioconjugation chemistry, particularly PEGylation technologies that extend circulation half-life and reduce immunogenicity.

Route of administration has evolved substantially from early parenteral-only formulations to include diverse delivery modalities. Oral formulations now exist for immunosuppressants (voclosporin, octreotide), antiviral agents (voxilaprevir, grazoprevir), and gastrointestinal therapeutics (plecanatide, linaclotide). Alternative routes include nasal administration (desmopressin, salmon calcitonin), specialized intrathecal delivery (ziconotide for severe chronic pain), and subcutaneous self-administration (zilucoplan, setmelanotide, vosoritide). These delivery innovations have significantly improved patient convenience, adherence, and quality of life, particularly for chronic conditions requiring long-term therapy.

The clinical landscape of cyclic peptides demonstrates progression in molecular design, target selection, and diversified therapeutic indications. The field has transitioned from traditional drug discovery to rational, structure-guided therapeutic development. This evolution positions cyclic peptides as a mature yet still-expanding drug class capable of addressing complex pathophysiology across diverse therapeutic areas.

The therapeutic landscape of cyclic peptides has experienced remarkable growth. Highlighted in Table 3 and below are 11 promising candidates currently advancing through clinical development. Current drug candidates represent diverse therapeutic areas from oncology to infectious diseases. These macrocyclic compounds leverage the structural advantages of peptides, including high selectivity and potency, reducing off-target effects, while addressing traditional limitations through innovative delivery systems and novel mechanisms of action.^69^

**Table 3:** Highlighted Cyclic Peptides in Clinical Development^a^ ^b^

Drug	CAS RN	Indications	Molar Mass (g/mol)	Target	Delivery route	Clinical trials (NCT)	Phase/Status	Company, location
ALXN2420	n/a	Acromegaly	Not disclosed	Growth hormone receptor	S.C.	NCT07037420	Phase II/Recruiting	Alexion, USA
AMY-101 (compstatin 40)	1427001–89–5	Gingivitis	1,789.10	Complement C3	Intragingival injection	NCT03694444	Phase II/Completed	Amyndas Pharmaceuticals, USA
Balixafortide (POL6326)	1051366–32–5	Metastatic pancreatic ductal adenocarcinoma	1,864.10	CXCR4	I.V.	NCT06981806	Phase I/Recruiting	Spexis, Switzerland
BT1718	2227366–66–5	Advanced solid	3,511.40	Tubulin	I.V.	NCT03486730	Phase II/Completed	Bicycle Therapeutics, UK
BT5528	2648849–70–9	EphA2+ advanced solid tumors	4,403.14	EphA2	I.V.	NCT04180371	Phase II/Active	Bicycle Therapeutics, UK
BT7480	2762739–36–4	Nectin-4/CD137+ advanced solid tumors	7,200.00	Nectin-4 (tumor) and CD137 (immune)	I.V.	NCT05163041	Phase II/Active	Bicycle Therapeutics, UK
CAP-232 (TT-232)	147159–51–1	Renal cell carcinoma	947.14	Somatostatin receptor (SSTR1/4)	I.V.	NCT00422786	Phase II/Completed	Thallion Pharmaceuticals, Canada
Certepetide (LSTA1)	2580154–02–3	Metastatic PDAC	989.09	αv-integrins and NRP-1	I.V.	NCT05042128	Phase II/Active	Lisata Therapeutics, USA
Dolcanatide (SP-333)	1092457–65–2	Opioid-induced constipation; colorectal cancer prevention	1,681.89	GC-C (guanylate cyclase-C)	Oral	NCT01983306; NCT03300570	Phase II/Completed; Phase I/Completed	Bausch Health, Canada
Icotrokinra (JNJ-77242113)	2763602–16–8	Psoriatic Arthritis; Ulcerative colitis	1,898.17	IL-23 receptor	Oral	NCT06878404/NCT07196748	Phase III/Recruiting; Phase III/Recruiting	Johnson & Johnson, USA
Lonodelestat (POL6014)	906547–89–5	Cystic fibrosis	1,478.73	Human neutrophil elastase	Inhalation	NCT03748199	Phase II/Completed	Santhera Pharmaceuticals, Switzerland
MK-0616	2861205–06–1	Hypercholesterolemia	1,722.09	PCSK9	Oral	NCT06492291	Phase III	Merck, USA
Noraramtide (BHV-1100)	2580150–96–3	Multiple myeloma	4,633.11	CD38	I.V.	NCT04634435	Phase 1/Completed	Biohaven Pharmaceuticals, Dana Farber Cancer Institute, US
Paluratide (LUNA18)	2676177–63–0	KRAS mutant solid tumors	1,437.68	RAS proteins	Oral	NCT05012618	Phase I/Active	Chugai Pharmaceutical, Japan
PL8177	n/a	Ulcerative colitis	996.13	Melanocortin-1 receptor	Oral (gut-restricted)	NCT05466890	Phase II/Active	Palatin Technologies, USA
PL9643	n/a	Dry eye disease	Not disclosed	Melanocortin receptor (MC1R-5R)	Ophthalmic solution	NCT05201170	Phase III/Completed	Palatin Technologies, USA
Rusfertide (PTG-300)	1628323–80–7	Polycythemia vera	2,441.96	Ferroportin	S.C.	NCT05210790	Phase III/Active	Protagonist Therapeutics, USA
Solnatide (AP301)	259206–53–6	ARDS	1,923.10	ENaC	Inhalation	NCT03567577	Phase II	APEPTICO, Austria
Sulanemadlin (ALRN-6924)	1451199–98–6	Advanced solid tumors, lymphomas	1,930.25	MDM2/MDMX (p53 activator)	I.V.	NCT02264613	Phase II/Completed	Aileron Therapeutics, USA
VT1021	2942310–76–9	Glioblastoma	Not disclosed	CD36/CD47	I.V.	NCT03970447	Phase III/Recruiting	Vigeo Therapeutics, USA
Zelenectide Pevedotin (BT8009)	2407446–48–2	Nectin-4 expressing advanced tumors; advanced or metastatic urothelial cancer	4,171.00	Nectin-4	I.V.	NCT04561362; NCT06225596	Phase II/Active; Phase III/Recruiting	Bicycle Therapeutics, UK
Zolucatetide (FOG-001)	3044032–95–0	Solid tumors with Wnt pathway activation	2,076.40	β-catenin–TCF4 interaction	I.V.	NCT05919264	Phase II/Recruiting	Parabilis Medicines, USA

a Source: CAS Content Collection and ClinicalTrials.gov.^131^

b ARDS, acute respiratory distress syndrome; I.V., intravenous; n/a, not available; S.C., subcutaneous.

### 5.1 Oncology Indications

Certepetide (LSTA-1), with a molar mass of 989.09 g/mol, demonstrates the potential of dual-targeting strategies in cancer therapy. This cyclic peptide simultaneously engages αv-integrins and neuropilin-1 (NRP-1), both critical mediators of tumor angiogenesis and metastasis.^11^ Certa Therapeutics has advanced this compound to Phase II trials (NCT05042128) for metastatic pancreatic ductal adenocarcinoma through the targeting of pathways involved in pancreatic cancer progression.

Paluratide (LUNA18) exemplifies the growing interest in cyclic peptides for oncology, particularly in targeting previously “undruggable” pathways. This 1,437.68 g/mol macrocyclic compound disrupts RAS–SOS1 interactions, a key driver of KRAS mutant solid tumors.^11^ Developed by Chugai Pharmaceutical, LUNA18 is being evaluated in Phase I trials (NCT05012618) as an oral monotherapy and in combination with cetuximab. By directly interfering with pan-RAS signaling, LUNA18 addresses a long-standing challenge in cancer therapy, effectively targeting KRAS mutations. Its oral administration and novel mechanism of action position LUNA18 as a potential first-in-class agent in the RAS-targeted therapy space, offering hope for patients with limited treatment options.

VT1021 represents a novel approach to glioblastoma treatment, one of the most challenging malignancies in oncology. This 638.76 g/mol cyclic peptide targets the CD36/CD47 axis, a critical immune checkpoint pathway that enables tumor immune evasion, currently being evaluated in Phase III trials (NCT03970447). Developed by Vigeo Therapeutics, VT1021 addresses the urgent need for effective treatments that can cross the blood–brain barrier and modulate the immunosuppressive tumor microenvironment characteristic of glioblastoma.^132^

In the realm of antibody-drug conjugates, zelenectide pevedotin (BT8009) exemplifies the evolution toward bicyclic peptide-drug conjugates. With a molar mass of approximately 4171 g/mol, this innovative therapeutic targets Nectin-4-expressing tumors.^11^ Phase III trial NCT06225596 is evaluating BT8009 in the treatment of urothelial cancer, while Phase II trial NCT04561362 is evaluating the treatment of Nectin-4-expressing advanced malignancies. Bicycle Therapeutics has engineered this compound to combine the selectivity of cyclic peptides with the cytotoxic potency of traditional chemotherapy.^133^ Zelenectide pevedotin demonstrates how cyclic peptide scaffolds can serve as precision delivery vehicles for cytotoxic payloads, potentially reducing systemic toxicity while enhancing therapeutic efficacy.

### 5.2 Nononcology Indications

In the endocrinology space, AZP-3813 demonstrates the potential of cyclic peptides as growth hormone receptor antagonists for acromegaly treatment. AZP-3813, currently in Phase I development, represents an alternative to existing somatostatin analogs, potentially offering improved selectivity and reduced side effects through S.C. administration.

Icotrokinra represents a major advancement in oral peptide therapeutics for immune-mediated diseases. This ∼1900 g/mol cyclic peptide targets the IL-23 receptor, a critical node in the inflammatory cascade implicated in psoriasis and ulcerative colitis.^11^ Developed by Johnson & Johnson in collaboration with Protagonist Therapeutics, Icotrokinra is currently in Phase III trials (NCT06878404) under the ICONIC program for moderate-to-severe psoriatic arthritis, with additional studies ongoing for ulcerative colitis (NCT07196748). The oral delivery system marks a significant technological achievement, offering patients a convenient alternative to injectable biologics while maintaining high target selectivity and potency. If successful, Icotrokinra could redefine treatment paradigms for chronic inflammatory disorders by combining biologic-like efficacy with oral dosing convenience.^134^

MK-0616 represents a breakthrough in oral peptide delivery for cardiovascular disease. This 1,722.09 g/mol proprotein convertase subtilisin/kexin type 9 (PCSK9) inhibitor, developed by Merck, has reached Phase III trials (NCT06492291) as an oral treatment for hypercholesterolemia. The successful development of an orally bioavailable cyclic peptide targeting PCSK9 represents a significant technological achievement, potentially offering the efficacy of injectable PCSK9 inhibitors with the convenience of oral administration.^135^

PL8177 showcases the versatility of cyclic peptides in inflammatory diseases, specifically targeting melanocortin-1 receptor for active ulcerative colitis treatment in Phase II trials (NCT05466890). Developed by Palatin Technologies, this 996.13 g/mol compound employs a gut-restricted oral delivery system, representing a significant advancement in peptide formulation technology.^136^ The ability to achieve local gastrointestinal activity while minimizing systemic exposure addresses a key challenge in inflammatory bowel disease treatment, potentially reducing side effects associated with systemic immunosuppression.

Complementing PL8177, PL9643 demonstrates Palatin Technologies’ expertise in melanocortin receptor modulation across different therapeutic areas. This cyclic peptide targets multiple melanocortin receptors (MC1*R*/3R/4*R*/5R) for dry eye disease treatment through ophthalmic delivery in Phase II trials (NCT05201170). PL9643 represents the growing recognition of melanocortin pathways in ocular inflammation and the potential for topical peptide therapeutics to address unmet needs in ophthalmology.^137^

Rusfertide (PTG-300) exemplifies the application of cyclic peptides in hematologic disorders, specifically targeting the hepcidin pathway for polycythemia vera treatment. This 2,441.96 g/mol compound, developed by Protagonist Therapeutics, has advanced to Phase III trials (NCT05210790) with S.C. delivery. Rusfertide represents a mechanistically novel approach to managing iron metabolism disorders, potentially offering improved efficacy and tolerability compared to traditional phlebotomy-based treatments.^138^

Finally, Solnatide (AP301) addresses critical care medicine through targeting of epithelial sodium channels (ENaC) for acute respiratory distress syndrome (ARDS) treatment. Developed by APEPTICO, this 1,923.10 g/mol cyclic peptide utilizes inhaled delivery to directly target pulmonary epithelium, promoting alveolar fluid clearance, a critical mechanism in ARDS recovery.^139^ The compound’s progression to Phase II trials (NCT03567577) represents a significant advancement in critical care therapeutics, where few effective treatments currently exist.

Collectively, these 11 cyclic peptides demonstrate the maturation of peptide therapeutics as a drug class, with successful applications across oncology, inflammatory conditions, hematology, critical care, endocrinology, and cardiovascular medicine. The diversity of targets, delivery systems, and therapeutic applications highlights the versatility of cyclic peptide scaffolds in addressing complex medical challenges. We utilized the workflow mentioned in this manuscript (Methods section in Supporting Information) and analyzed the candidates in clinical development, listed in Table 3. The dominance of macrocyclic and bicyclic or polycyclic peptides and head-to-tail cyclization is closely aligned with the distribution observed in the CAS data set (Figure 3), underscoring the translational relevance of these architectures from chemical space to clinical development. Among the modifications, disulfide linkages and *N*-methylation are the most common among candidates which are currently in clinical development (Supplementary Table S5). The advancement of multiple compounds to late-stage clinical trials suggests that cyclic peptides are poised to become a significant component of the therapeutic arsenal across multiple disease areas, offering the potential for improved efficacy, selectivity, and patient outcomes compared to traditional small molecules and biologic approaches.

### 5.3 Physicochemical Characteristics of Approved and Clinical-Stage Cyclic Peptides

To anchor the broader physicochemical trends in a translational context, we examined the properties of approved cyclic peptide drugs and selected clinical-stage candidates (Tables 4 and 5, S6 and S7 for structures). These compounds represent a highly selective subset of the cyclic peptide design space and illustrate the physicochemical boundaries compatible with clinical advancement.

**Table 4:** Physiochemical Properties of Selected Approved Cyclic Peptides (2010 Onwards)^a^ ^b^

Drug	CAS RN	H bond acceptors	H bond donors	Freely rotatable bonds	Log *P*	MW	Molar refractivity (m^3^/mol)	Number of atoms	Log *S*	Log BB	PSA (Å^2^)	Plasma protein binding	Predicted toxicity (EPA category)	LD_50_ (mg/kg)	Predicted p*K*_a_
Rezafungin (Rezzayo)	1396640–59–7	25	13	18	–5.447	1226.39	317.38	88	–6.47	–4.14	366.42	70.41	III	1438	9.859 ± 0.26
Motixafortide (Aphexda)	664334–36–5	52	43	53	–6.742	2159.53	560.61	152	–15.57	–6.81	942.64	51.03	I	9.92	NR
Zilucoplan (Zilbrysq)	1841136–73–9	NR	NR	NR	NR	NR	NR	NR	–18.26	–9.89	NR	100	I	10.4	NR
Voclosporin (Lupkynis)	515814–01–4	23	5	16	1.85	1214.62	332.68	86	–11.32	–1.71	278.8	100	III	1160	13.315 ± 0.70
Setmelanotide (Imcivree)	920014–72–8	27	20	20	–3.175	1117.31	290.34	78	–9.02	–4.46	497.76	23.15	III	1193.53	12.818 ± 0.70
Bremelanotide (Vyleesi)	189691–06–3	24	15	18	–1.14	1025.16	268.58	74	–8.55	–4	376.47	53.24	III	1728.05	3.371 ± 0.70
177Lu-DOTATATE (Lutathera)	437608–50–9	NR	NR	NR	NR	1609.55	343.19	101	NR	NR	NR	NR	NR	NR	NR
Plitidepsin (Aplidin)	137219–37–5	22	4	15	1.0003	1110.34	288.59	79	–9.61	–1.98	284.74	99.94	III	1750.18	11.278 ± 0.70
Plecanatide (Trulance)	467426–54–6	44	26	28	–5.693	1681.89	398.46	113	–3.72	–7.87	819.33	0	I	15.5	3.606 ± 0.21
Voxilaprevir (Vosevi)	1535212–07–7	15	3	9	4.171	868.94	202.66	60	–8.62	–0.49	203.6	95.48	III	3139.78	5.485 ± 0.40
Grazoprevir (Zepatier)	1350514–68–9	15	3	8	3.935	766.91	193.37	54	–6.42	–0.84	203.6	73.86	III	3514.21	5.511 ± 0.40
Oritavancin (Orbactiv)	171099–57–3	36	22	19	2.651	1793.1	440.43	125	–14.59	–5.69	560.98	100	III	2474.05	2.929 ± 0.70
Terlipressin (Glypressin)	14636–12–5	31	21	25	–7.085	1227.38	305.69	85	–6.01	–3.6	563.45	0	I	8.01	9.901 ± 0.15
Linaclotide (Linzess)	851199–59–2	36	21	13	–6.127	1526.75	367.4	101	NR	NR	725.71	0	I	14.66	3.053 ± 0.10
Pasireotide (Signifor)	396091–73–9	19	11	18	2.712	1047.21	287.04	77	–11.8	–1.48	281.2	87.61	III	848.5	11.858 ± 0.46
Median values		25	15	18	–1.14	1220.5	305.69	85	–8.82	–3.8	376.47	70.41	III	1193.53	5.51

a Source: CAS Content Collection.

b NR = Not reported.

**Table 5:** Physiochemical Properties of Cyclic Peptides in Clinical Development^a^ ^b^

Drug	CAS RN	H bond acceptors	H bond donors	Freely rotatable bonds	Log *P*	MW	Molar refractivity (m^3^/mol)	Number of atoms	Log *S*	Log BB	PSA (Å^2^)	Plasma protein binding	Predicted toxicity (EPA category)	LD_50_ (mg/kg)	Predicted p*K*_a_
AMY-101 (Compstatin 40)	1427001895	41	25	30	–2.828	1789.1	464.67	126	–13.11	–5.85	692.35	52.58	III	2537.89	4.004 ± 0.10
Balixafortide (POL6326)	1051366325	45	28	23	–10.582	1864.1	472.95	131	–8.63	–6.01	756.46	0	III	1079.48	9.890 ± 0.15
BT1718	2227366–66–5	78	27	60	–3.405	3511.4	885.45	243	–15.71	–12.3	1207.3	43.07	NR	NR	NR
CAP-232 (TT-232)	147159–51–1	19	15	16	0.451	947.14	250.01	66	–5.65	–2.38	376.58	42.78	III	667.26	9.901 ± 0.15
Certepetide (LSTA1)	2580154–02–3	28	18	15	–8.305	989.09	235.48	67	–2.79	–4.62	509.32	0	III	582.19	4.005 ± 0.10
Dolcanatide (SP-333)	1092457–65–2	44	26	28	–5.693	1681.89	398.46	113	–3.72	–7.87	819.33	0	II	470.99	4.445 ± 0.10
Icotrokinra (JNJ-77242113)	2763602–16–8	42	24	40	–1.911	1898.17	488.84	134	–10.64	–6.14	709.17	19.17	III	819.9	4.445 ± 0.10
Lonodelestat (POL6014)	906547–89–5	34	17	23	–4.755	1478.73	NR	NR	NR	NR	NR	NR	NR	NR	NR
Paluratide (LUNA18)	2676177–63–0	24	3	13	2.742	1437.68	369.87	102	–13.7	–1.53	270.09	100	III	842.76	13.236 ± 0.70
Rusfertide (PTG-300)	1628323–80–7	55	33	67	–0.894	2441.96	NR	NR	NR	NR	NR	NR	NR	NR	NR
Solnatide (AP301)	259206–53–6	50	31	27	–13.017	1923.10	471.7	134	–5.86	–8.65	861.1	0	I	9.05	2.951 ± 0.70
Sulanemadlin (ALRN-6924)	1451199–98–6	43	25	37	0.051	1930.25	502.91	138	–14.22	–5.79	674.43	51.94	I	7.44	4.481 ± 0.10
Zolucatetide (FOG-001)	3044032–95–0	43	20	18	3.014	2076.4	NR	NR	NR	NR	NR	NR	NR	NR	NR
Median values		43	25	27	–2.82	1826.6	468.18	128.5	–9.63	–5.93	700.76	30.97	III	667.26	4.445

a Source: CAS Content Collection.

b NR = Not reported.

Approved and late-stage cyclic peptides consistently exceed classical small-molecule thresholds for MW and polar surface area, frequently approaching or surpassing 1 kDa and 500–1,000 Å^2^, respectively. Clinical success within this regime is therefore not driven by adherence to conventional drug-likeness criteria, but by compensatory features such as conformational constraint, stability, and context-dependent polarity masking. Parenterally administered peptides tolerate particularly high polarity and hydrogen-bonding capacity, whereas the few orally administered examples occupy a noticeably narrower property window, consistent with partial shielding of polar functionality and limited effective flexibility.

Clinical-stage candidates further highlight the sources of attrition, with many exhibiting extreme molecular size, high flexibility, poor solubility, or unfavorable permeability predictions. These properties likely contribute to rapid clearance, restricted tissue distribution, or formulation challenges. Collectively, these benchmarks emphasize that cyclization alone does not ensure developability; instead, successful cyclic peptide therapeutics emerge from a narrow, context-dependent balance of size, polarity, flexibility, and stability tailored to the route of administration and target accessibility.

### 5.4 Clinical Advancement and Attrition in Cyclic Peptide Development

Our above analysis is inherently subject to survivorship bias, as it focuses on successful compounds reported in the literature and active clinical pipelines. Failed programs particularly those discontinued in preclinical or early clinical phases are systematically underreported, creating a significant knowledge gap. The structural features we identify in successful compounds (*N*-methylation, appropriate macrocycle size, stabilized secondary structures) are contributing factors that can enhance, but do not ensure, the likelihood of clinical advancement. A few failed examples of cyclic peptide therapeutics are discussed to provide some balance to our analysis.

Cilengitide, a cyclic pentapeptide integrin αvβ3/αvβ5 inhibitor with *N*-methylated valine and d-phenylalanine for enhanced stability, achieved high receptor affinity (*K*_d_ = 0.6 nM) and brain penetration. However, it failed to meet its primary end point in the Phase III CENTRIC trial (NCT00689221/2013) for glioblastoma with median overall survival of 26.3 months for both control and therapeutic groups for no survival benefit. Failure was attributed to target biology complexity; integrin inhibition may paradoxically promote invasion and suboptimal dosing schedules.^140,141^ This illustrates that structural optimization (*N*-methylation, cyclization) successfully addressed stability and affinity but could not overcome inadequate target validation and complex tumor biology.

Romidepsin (FK228), a bicyclic depsipeptide HDAC inhibitor approved in 2009, contains an intramolecular disulfide bond that undergoes reductive cleavage within cells to unmask a zinc-binding thiol as the pharmacologically active species.^142^ Despite poor oral bioavailability (<10%) and the disulfide being viewed as a metabolic liability due to reduction by glutathione and thioredoxin in plasma, romidepsin succeeded where more “stable” analogues failed. Structure–activity relationship studies on spiruchostatin-based compounds with thioether replacements, FK228 lactam variants with amide bonds replacing the depsipeptide ester, and largazole derivatives with nonreducible linkers all demonstrated 10- to 100-fold reduced HDAC inhibition despite improved metabolic stability.^143^ These findings illustrate that the disulfide serves dual purposes: enabling cellular uptake as a lipophilic prodrug and providing conformational flexibility upon reduction for optimal active site engagement. Overstabilization eliminated these beneficial features, demonstrating that apparent structural liabilities can be integral to the therapeutic mechanism.

Despite their promise, many cyclic peptides fail to progress beyond early development due to a combination of pharmacokinetic and formulation challenges. Rapid systemic clearance often driven by renal filtration and residual proteolytic susceptibility remains a major limitation, particularly for peptides lacking sufficient size, plasma protein binding, or stabilizing modifications. In addition, poor tissue penetration and unfavorable biodistribution can restrict efficacy, especially for intracellular or deep-tissue targets. Even when stability is improved through cyclization, the high polarity and hydrogen-bonding capacity of most cyclic peptides continue to limit membrane permeability, necessitating parenteral administration for the majority of candidates. Formulation challenges, including solubility, aggregation, and manufacturability, further complicate clinical translation. Together, these factors contribute to high attrition rates and underscore that cyclization alone does not guarantee favorable drug-like properties. Addressing these limitations requires integrated optimization of structure, physicochemical properties, delivery strategies, and formulation, highlighting the need for continued innovation to fully realize the therapeutic potential of cyclic peptides.

Together, these challenges highlight that realizing the full therapeutic potential of cyclic peptides will depend on continued advances in design, delivery, and enabling technologies, which are discussed in the following section.

## 6 Future Directions

Cyclic peptides have emerged as a transformative modality in drug development, bridging the gap between small molecules and biologics. Their unique structural features, conformational rigidity, enhanced proteolytic stability, and high target specificity, make them ideal candidates for addressing previously “undruggable” targets such as PPIs. Nevertheless, despite notable successes, many challenges remain and with them, many exciting opportunities for future research. Below we highlight key directions likely to propel the field in the coming years.

### 6.1 Enhancing Oral Bioavailability and Cell Permeability

One of the most critical challenges for cyclic peptide therapeutics remains achieving oral bioavailability and cellular uptake, particularly for intracellular targets. Current strategies focus on multiple complementary approaches to overcome these barriers. Structural modifications are at the forefront of permeability enhancement. *N*-methylation of backbone amides has proven effective in reducing polar surface area while maintaining target affinity, as demonstrated by successful oral cyclic peptides like cyclosporine. Incorporation of noncanonical amino acids, including d-amino acids and β-amino acids, not only enhances permeability but also provides proteolytic resistance. Lipidation strategies, exemplified by semaglutide’s fatty acid modification, have shown promise in improving both membrane permeability and pharmacokinetic profiles.^55,144,145^ Intramolecular hydrogen bonds, particularly those involving backbone amide carbonyls, enable partial shielding of polar functionality and reduce the energetic penalty associated with membrane desolvation. In parallel, conformational shielding, the ability of a cyclic peptide to adopt compact, folded conformations in nonpolar environments while remaining solvent-exposed in aqueous media plays a critical role in enabling permeability without sacrificing solubility. Importantly, these features must be carefully balanced, as over-rigidification can limit adaptive folding, while insufficient constraint exposes polar surface area and impairs permeability.^146^

Formulation approaches are equally important. Intestinal permeation enhancers such as sodium caprate and SNAC (sodium *N*-[8-(2-hydroxybenzoyl)­amino]­caprylate) have enabled oral delivery of peptides previously limited to parenteral administration.^147^ Nanoparticle encapsulation systems, including solid lipid nanoparticles and polymeric micelles, protect cyclic peptides from enzymatic degradation while facilitating transcellular transport.^148^ Microneedle patches represent an alternative transdermal delivery route that bypasses gastrointestinal barriers entirely.^149^ Additionally, designing cyclic peptides for active transport via peptide transporters (PepT1, PepT2) or exploiting transcytosis pathways can significantly improve oral bioavailability.^150^ These innovations collectively promise to transition cyclic peptides from injectable formulations to patient-friendly oral therapeutics within the coming decade.

### 6.2 De Novo Design and AI-Driven Discovery

The landscape of cyclic peptide discovery is rapidly evolving from natural product modification to computational de novo design powered by artificial intelligence (AI). As Heinis and colleagues emphasized, “new powerful techniques based on rational design and in vitro evolution have enabled the de novo development of cyclic peptide ligands to targets for which nature does not offer solutions.”^151^ Generative AI models, such as variational autoencoders (VAEs) and generative adversarial networks (GANs), have enabled the design of cyclic peptide sequences with optimized properties.^152,153^ More recently, diffusion models like CycleDesigner and RFpeptides have shown promise in generating novel macrocyclic scaffolds by learning the distribution of successful peptide structures.^154,155^

Reinforcement learning (RL) frameworks, including CCPep and PepThink-R1, optimize multiple properties simultaneously like balancing affinity, selectivity, stability, and permeability.^156,157^ Specialized platforms such as PepFlow (flow-matching generative model), CycPeptMPNN (graph-based neural network for cyclic peptides), MultiCycPermea (multimodal permeability prediction), and CyclicChamp (energy-based heuristic design), address unique challenges like ring closure compatibility, conformational preferences, and membrane permeability.^102,158,159,160^

Structure prediction tools have also advanced: AlphaFold3 and its adaptations (e.g., AfCycDesign) now model cyclic peptides with noncanonical amino acids and disulfide bonds with atomic-level accuracy.^161^ Rosetta, Des3PI, and the cyclicpeptide Python package integrate structural modeling with predictive analytics for early-stage property analysis.^162,163,164^ Integrated modeling suites and machine learning-guided tools like NCPepFold predict optimal cyclization strategies and ring conformations, accelerating the design-to-validation pipeline.^165^

By combining these AI-driven platforms with automated synthesis and screening, the field is poised to dramatically shorten discovery timelines, transforming cyclic peptide development from years to months, especially for targets previously considered intractable.

### 6.3 Genetically Encoded and Display-Based Macrocycle Discovery Platforms

mRNA display-based technologies, including the RaPID (Random nonstandard Peptides Integrated Discovery) platform, have become powerful drivers of cyclic peptide discovery by enabling the screening of extremely large libraries (10^9^–10^12^ variants) against challenging biological targets. In mRNA display, peptides are covalently linked to their encoding mRNA via a puromycin tag, establishing a robust genotype–phenotype connection that allows rapid selection and amplification of high-affinity binders. RaPID further expands this capability by combining flexible in vitro translation with mRNA display to generate covalently cyclized peptide libraries incorporating diverse noncanonical amino acids and tailored cyclization chemistries, such as thioether linkages. These features significantly extend accessible chemical space beyond that of ribosomal peptides composed solely of natural residues. Cyclization within these libraries reduces conformational flexibility, lowering the entropic cost of target binding and improving proteolytic stability, while enabling high affinity and selectivity for difficult targets such as protein–protein interfaces.^166,167^

Recent applications of mRNA display/RaPID-derived cyclic peptides illustrate the breadth of this platform across discovery and mechanistic research. Cyclic peptide scaffolds identified by mRNA display have been used as chemical probes to interrogate enzyme mechanisms, exemplified by inhibitors of mammalian N-terminal cysteine oxidase (ADO) that revealed key binding interactions and catalytic residues.^168^ In therapeutic discovery, optimized mRNA-display workflows have yielded high-affinity bicyclic peptide inhibitors of FGFR3c that outperform linear and monocyclic counterparts in both potency and plasma stability.^169^ The technology has also enabled targeting of structurally challenging or underexplored protein domains, such as the extraterminal domain of BRD3, by identifying cyclic peptide binders directly from mammalian cell lysates.^170^

Genetically encoded macrocycle libraries provide a powerful platform for cyclic peptide discovery by enabling the generation of highly diverse libraries with precise control over structural properties while maintaining a direct genotype–phenotype linkage. These systems allow the incorporation of noncanonical amino acids and predefined cyclization motifs, thereby expanding accessible chemical space beyond conventional peptide libraries. When coupled with selection methodologies such as display technologies, genetically encoded libraries facilitate rapid identification of cyclic peptides with high affinity and selectivity for challenging targets. As library design and chemical encoding strategies continue to advance, these platforms are expected to play an increasingly important role in discovering cyclic peptide scaffolds optimized for both target engagement and developability.^171^

Together, these technologies provide a versatile and scalable platform for both mechanistic investigation and therapeutic lead discovery.

### 6.4 Expanding the Druggable Proteome

Cyclic peptides are expanding the druggable proteome by enabling access to protein targets previously considered undruggable, such as transcription factors, scaffolding proteins, and intrinsically disordered proteins.^172,173^ These molecules can engage shallow or dynamic surfaces, induce conformational changes, and disrupt PPIs critical to disease pathways. Notable successes include cyclic peptides that inhibit the MDM2–p53 interaction, modulate BCL-2 family proteins, and target RAS-effector complexes, once deemed intractable.^174,175^ Advances in fragment-based and structure-guided design, combined with display technologies like mRNA and phage display, facilitate the discovery of potent cyclic binders.^163,176^ Moreover, the integration of covalent warheads into cyclic frameworks enhances specificity and expands targeting capabilities, positioning cyclic peptides as versatile tools in next-generation drug discovery.

### 6.5 Novel Cyclization and Stabilization Techniques

Advances in synthetic chemistry are enabling precise control over cyclic peptide architecture and stability, driving innovation in drug design. Orthogonal cyclization strategies, including head-to-tail, side-chain-to-side-chain (e.g., disulfide bridges, lactam bonds, hydrocarbon staples), and backbone cyclization, enhance conformational rigidity and proteolytic resistance.^42^ Enzymatic methods such as cyanobactin macrocyclases, split-intein systems, and sortase-mediated ligation offer site-specific and traceless cyclization under mild conditions.^177,178,179^ Click chemistry approaches like copper-catalyzed azide–alkyne cycloaddition (CuAAC), strain-promoted azide–alkyne cycloaddition (SPAAC), and tetrazine-alkene cycloadditions provide bioorthogonal and rapid macrocycle formation.^180^ Metal-mediated techniques, including ruthenium-catalyzed ring-closing metathesis and palladium-catalyzed cross-coupling, introduce structural rigidity and enable incorporation of aromatic linkers.^181^ Manufacturing innovations, automated solid-phase synthesis, microwave-assisted cyclization, and flow chemistry, are improving scalability, reproducibility, and cost-effectiveness, making cyclic peptide production more commercially viable.^182^

### 6.6 Conjugation and Multifunctional Platforms

Cyclic peptides emerge as versatile scaffolds for multifunctional therapeutic platforms, extending beyond traditional single-agent modalities. Peptide-drug conjugates (PDCs) combine targeting specificity with cytotoxic payloads, using cleavable linkers (e.g., hydrazone, disulfide, cathepsin-sensitive) or noncleavable linkers to enhance therapeutic indices.^183^ Bispecific formats, including peptidic bispecific antibodies, enable simultaneous engagement of multiple targets, showing promise in immunotherapy and resistance mitigation.^184^ Nanoparticle conjugation strategies utilize cyclic peptides like RGD (arginine (R), glycine (G), and aspartic acid (D)) for integrin-targeted delivery, improving biodistribution and reducing off-target effects.^185^ In PROTAC applications, cyclic peptides act as E3 ligase recruiters or target binders, facilitating targeted protein degradation and expanding therapeutic scope.^186,187^ Theranostic platforms integrate cyclic peptides with imaging agents (e.g., PET tracers, fluorophores, MRI contrast agents), enabling real-time monitoring of drug distribution and response. These multifunctional strategies position cyclic peptides as central components in next-generation precision therapeutics.^188,189^

### 6.7 Emerging Therapeutic Areas

Cyclic peptides, while already established in therapeutic areas such as oncology, infectious diseases, and cardiovascular disorders, are poised to expand into new domains including neurological diseases, where they can target intracellular protein–protein interactions implicated in neurodegeneration.^190^ Their potential in metabolic disorders is also growing, with orally bioavailable macrocyclic peptides being explored for conditions like diabetes and obesity. In the realm of rare diseases and gene regulation, cyclic peptides are emerging as modulators of RNA-binding proteins and epigenetic regulators.^191,192^ Furthermore, their structural versatility makes them promising candidates in the fight against antimicrobial resistance, offering novel scaffolds capable of disrupting bacterial virulence pathways without inducing resistance mechanisms.^193,194^

## 7 Conclusion

Cyclic peptides have become an important focus in current drug discovery because they offer a balance between the selectivity of biologics and the stability of small molecules. Recent advances in synthesis, screening technologies, and design strategies have made it easier to generate diverse and more drug-like cyclic peptide structures. This progress is clearly reflected in the growing number of publications and patents in the past few years, indicating steadily increasing interest from both academia and industry.

In this study, we leveraged the data from CAS Content Collection, to provide a comprehensive data-driven overview of cyclic peptides and their therapeutic potential. Our analysis reveals a steady rise in research activity from 2006 to 2025, with journal publications predominating overall, but a recent surge in patent filings reflects increasing commercial interest. Patent classification trends highlight pharmaceuticals as the major innovation focus, alongside growing activity in immunochemistry, and production methodologies. Oral delivery has gained remarkable attention, underscoring advances aimed at improving metabolic stability and permeability of cyclic peptides, while most other routes remain modestly represented. Therapeutic area mapping identifies oncology as the dominant application space, followed by infectious and inflammatory diseases, and other emerging areas. Analyses of peptide architecture demonstrate a strong preference for macrocyclic, bicyclic, and polycyclic scaffolds, supported by dominant head-to-tail cyclization strategies and widespread use of stabilizing modifications such as disulfide bonds, thioethers, and *N*-methylation. Co-occurrence patterns between peptide features, administration routes, and therapeutic areas reveal that structural complexity, particularly in larger macrocycles, enables broader modification diversity and stronger alignment with oral and I.V. delivery, as well as with potentially challenging targets such as PD-1/PD-L1, Ras family proteins, p53, and SOS1. Together, these findings present a unified view of the evolving design principles, therapeutic priorities, and translational opportunities shaping the future landscape of cyclic peptide drug development.

Going forward, the field is likely to expand further as newer tools such as computational design and improved macrocyclization methods help address remaining challenges related to bioavailability, manufacturing, and clinical translation. Overall, cyclic peptides are increasingly established as a practical and versatile therapeutic modality, with clear potential for continued growth as technologies mature.

## Supplementary Material



## Notes

Supplementary Figure S1: publication trend related to peptide therapeutics, Supplementary Figure S2: leading patent assignees in the field of cyclic peptides, Supplementary Figures S3, S4, and S5: bibliometric analysis of leading research organizations and influential journals in cyclic peptide research, Supplementary Figure S6: number of documents contributing to CAS-indexed cyclic peptides, categorized by various parameters, Supplementary Figure S7: cyclic peptide types according to their MW, Supplementary Figure S8: individual scatter plots for MW versus PSA for each cyclization type, Supplementary Figure S9: individual scatter plots for MW versus Log *P* for each cyclization type, Supplementary Figure S10: delivery systems for cyclic peptides, Supplementary Tables S1, S2, and S3: distribution of cyclic peptide types or cyclization types or modifications across patents from leading commercial assignees, Supplementary Table S4: approved cyclic peptide therapeutics, Supplementary Table S5: SMILE-based analysis on the cyclic peptide therapeutics currently in clinical development, Supplementary Tables S6 and S7: structures and physicochemical properties of approved cyclic peptide drugs and clinical candidates (PDF)

## Abbreviations

AI: artificial intelligence
ARDS: acute respiratory distress syndrome
CAGR: compound annual growth rate
CuAAC: Cu­(I)-catalyzed azide–alkyne cycloaddition
CXCR4: chemokine receptor type 4
DDAVP: d-amino-8-d-arginine-vasopressin
ENaC: epithelial sodium channels
EphA2: ephrin type-A receptor 2
FDA: Food and Drug administration
GANs: generative adversarial networks
GC-C: guanylate cyclase-C
GI: gastrointestinal
HIP: hydrophobic ion pairing
I.V.: intravenous
IL-23: interleukin-23
MCR: melanocortin receptor
MDM: mouse double minute 2 homologue
MRI: magnetic resonance imaging
MRPs: multidrug resistance proteins
MUC: mucin
MW: molecular weight
NLCs: nanostructured lipid carriers
NRP-1: neuropilin-1
P-gp: P-glycoprotein
PCSK9: proprotein convertase subtilisin/kexin type 9
PD1: programmed cell death protein 1
PDC: peptide-drug conjugate
PDL1: programmed death-ligand 1
PDL2: programmed death-ligand 2
PEG: polyethylene glycol
PepT: peptide transporter
PET: positron emission tomography
PHA: polyhydroxyalkanoate
PK: pharmacokinetic
PLA: polylactic acid
PLGA: poly­(lactic-*co*-glycolic acid)
PPIs: protein–protein interactions
PROTAC: proteolysis targeting chimera
PSA RL: reinforcement learning
S.C.: subcutaneous
SEEDS: self-emulsifying drug delivery systems
SLNs: solid lipid nanoparticles
SNAC: sodium *N*-[8-(2-hydroxybenzoyl)­amino]­caprylate
SOMA: self-orienting millimeter-scale applicator
SOS1: Son of Sevenless homologue 1
SPAAC: strain-promoted azide–alkyne cycloaddition
SSTR: somatostatin receptor
STAT3: Signal Transducer and Activator of Transcription 3
TCF4: transcription factor 4
TNF: tumor necrosis factor
TP53: tumor protein 53
VAEs: variational autoencoders
