# Supplementary material for: Cyclic Peptides in Modern Drug Discovery: Trends and Therapeutic Directions
Source: J Med Chem. 2026 Jul 21;69(15):17770–99. doi: 10.1021/acs.jmedchem.6c01522 (PMC13492385; doi:10.1021/acs.jmedchem.6c01522)
Supplement: Supplementary file 1 [file jm6c01522_si_001.pdf]

Supplementary information for

Cyclic peptides in modern drug discovery: trends and therapeutic directions

Trupti Thite[1], Kavita A Iyer[2], Preeti Jain[1], Janet M Sasso[2], Rumiana Tenchov[2], Sudarshan Murmu[1], Mahaveer Surendranath[1], Ankush Maind[1], Umesh T Balande[1], Qiongqiong Angela Zhou[*,2]

[1]ACS International India Pvt. Ltd., Pune, 411044, India

[2]CAS, a division of the American Chemical Society, Columbus, Ohio 43210, United States

*Corresponding author; Email: qzhou@cas.org

Contents of SI:

- Methods
- Figures S1 to S10
- Tables S1 to S7
- References

Methods

1. Search strategy and data extraction:

CAS subject matter experts adjusted the search query iteratively to minimize noise while ensuring maximum coverage of the field resulting in a search query utilizing the following terms: ?CYCLIC? PEPTIDE? OR CYCLOPEPTIDE? OR CYCLOTIDE? OR PEPTIDES, CYCLIC OR LASSO PEPTIDE?. The search query resulted in 23,471 documents (journal and patent publications) over a period of 2006-2025. Data corresponding to the 23,471 documents was extracted from the CAS Content Collection and consisted of the following information: title, abstract, year of publication, number of citations, the first author's affiliation, the country/region of the affiliation as well as patent offices, patent assignees and claims in case of patent publications. In addition to bibliographic information, we also extracted concept data consisting of CAS indexed terms and their roles associated from the CAS Content Collection. Finally, substance data associated with documents restricted to the period 2020-2025 and having CAS roles therapeutic (THU), pharmacological (PAC) and pharmacokinetic (PKT) were also extracted. Data for 2025 is partial and includes data for January-August.

2. Data analysis:

Leading research organizations and journal publications were identified on the basis of volume of research output, number of citations, and a combination of both. Leading patent assignees were identified based on volume of patents filed and separated into commercial and non-commercial patent assignees. Geographical information (country/region) and organizational affiliation was that of the first author or the first patent assignee for journal and patent publications, respectively. Data analysis was performed using Tableau by searching for specific keywords consisting of multiple synonyms as well as abbreviations in the following fields: title, abstract, keywords, and CAS indexed terms associated with publications.

Figures were created using a combination of Tableau, Microsoft Excel and Microsoft PowerPoint. Where indicated/specified www.BioRender.com was also utilized for creating some of the schematic illustrations.

3. Identification of cyclic peptide, their cyclization types and modifications:

The following is a detailed description of the methodology used to identify cyclic peptides, classify the identified cyclic peptides into various types based on various parameters including number of amide bonds, types of cyclization and types of modifications achieved using Python and RDKit module [1]

Identification of cyclic peptides:

A molecule was considered a cyclic peptide if all three of the following conditions were true:

1. It contains at least one ring (has_any_ring = True).
2. It has ≥ 2 amide bonds overall (len(amide_bidx) >= 2).
3. It has a ring with ≥ 2 backbone-like amide bonds (both ends adjacent to α-carbons).

If any one of these rules was not satisfied, the peptide was classified as linear.

Identification of type of cyclic peptides:

Selected the ring with the highest number of amide bonds (most likely the main macrocycle) and counted all amide bonds in that ring (ignore α-carbon filter for size naming).

Applied the following classification:

2 amides → cyclic dipeptide

3 amides → cyclic tripeptide

4 amides → cyclic tetrapeptide

5 amides → cyclic pentapeptide

≥ 6 amides → macrocyclic peptide

If more than one amide-containing ring exists → bicyclic or polycyclic peptide.

Identification of type of modification:

We analyzed the identified cyclic peptides for the following modifications:

(i) Disulfide bond (–S–S–):

Detected sulfur–sulfur single bonds connecting two cysteine-like atoms. If at least one such linkage exists, the peptide is marked as disulfide-bond.

(ii) Glycosylation:

Identified five- or six-membered rings containing at least two oxygen atoms, with at least one exocyclic oxygen atom or oxygen-linked carbon atom outside the ring. Identified rings were interpreted as carbohydrate moieties attached to the peptide, marking glycosylation.

(iii) Lipidation:

Searched for continuous chains of eight or more sp^3-hybridized carbon atoms without heteroatoms (O, N, S). Presence of such a chain connected to either a terminal nitrogen atom (head) or terminal oxygen/carbonyl carbon atom (tail) was marked as lipidation.

(iv) Phosphorylation and Sulfation

Phosphorylation was identified by a phosphorus atom connected to an oxygen atom via a double bond while also being bonded to two additional oxygen atoms via singly bonds (P(=O)(O)(O)).

Sulfation was identified by a sulfur atom attached to two oxygen atoms via double-bonds and to a hydroxyl oxygen via a single bond (S(=O)(=O)(O)).

(v) N-methylation

N-methylation was inferred when an amide nitrogen atom lacked a hydrogen atom and was instead bound to an alkyl carbon atom.

(vi) PEG-like (polyethylene glycol) chain

Identified repeating $–O–CH_2–CH_2–$ patterns appearing more than twice consecutively signaling PEGylation or attachment of ethylene-oxide units.

4. **Physicochemical properties of cyclic peptides:**

The values for physicochemical properties discussed in section 3.4 (hydrogen bond donor, hydrogen bond acceptor, freely rotatable bonds, were retrieved form SciFinder which employs ACD/Labs algorithms for property prediction[2]. While Lipinski and Veber originally defined hydrogen-bond acceptors and rotatable bonds using simple, heuristic criteria, contemporary tools such as SciFinder and ACD/Labs apply structure-aware algorithms that account for protonation state, resonance, and conformational constraints, leading to systematic differences in descriptor values, particularly for cyclic peptides and macrocycles. These differences can significantly influence the apparent number and may lead to systematic differences in reported hydrogen-bond acceptor and rotatable bond counts compared

with other software platforms or manual rule interpretations. Consequently, the degree of rule compliance reported here should be interpreted relative to the specific descriptors used.

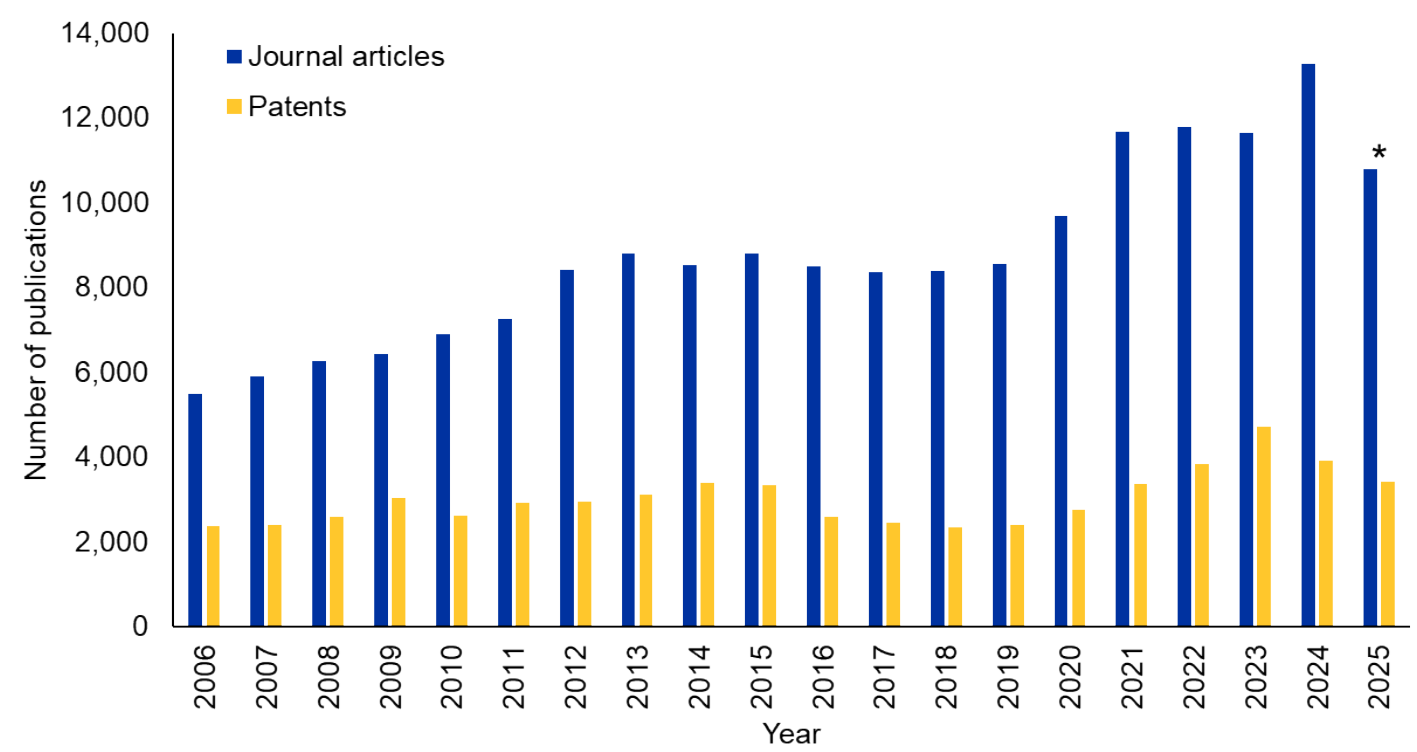

Figure S1: Yearly publication trend (journal articles and patents) related to peptide therapeutics. Source: STN, CAS Content Collection, *Data is till November 2025.

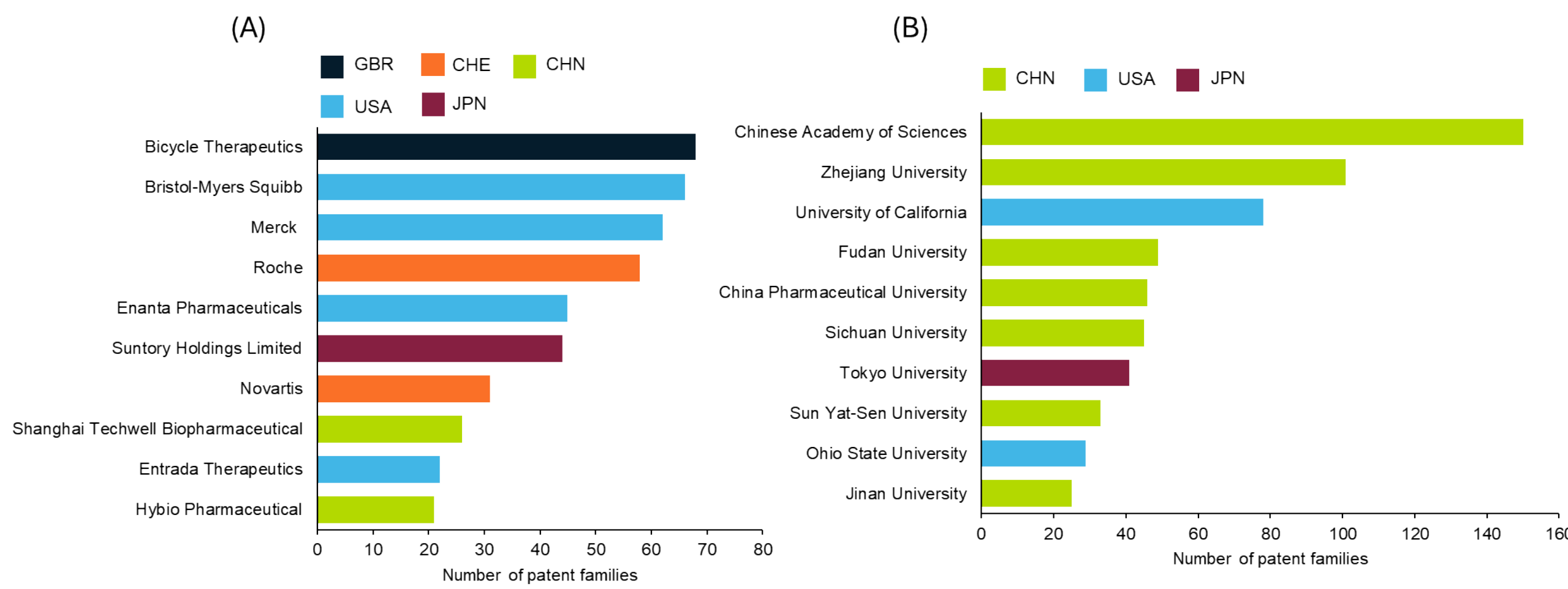

Figure S2: Leading (A) commercial and (B) non-commercial patent assignees related to cyclic peptides. Data includes patents related to cyclic peptides for the period 2006-2025. Source: CAS Content Collection. Standard three letter abbreviations used to represent countries: GBR, United Kingdom; CHE, Switzerland; CHN, China; USA, United States of America; JPN, Japan.

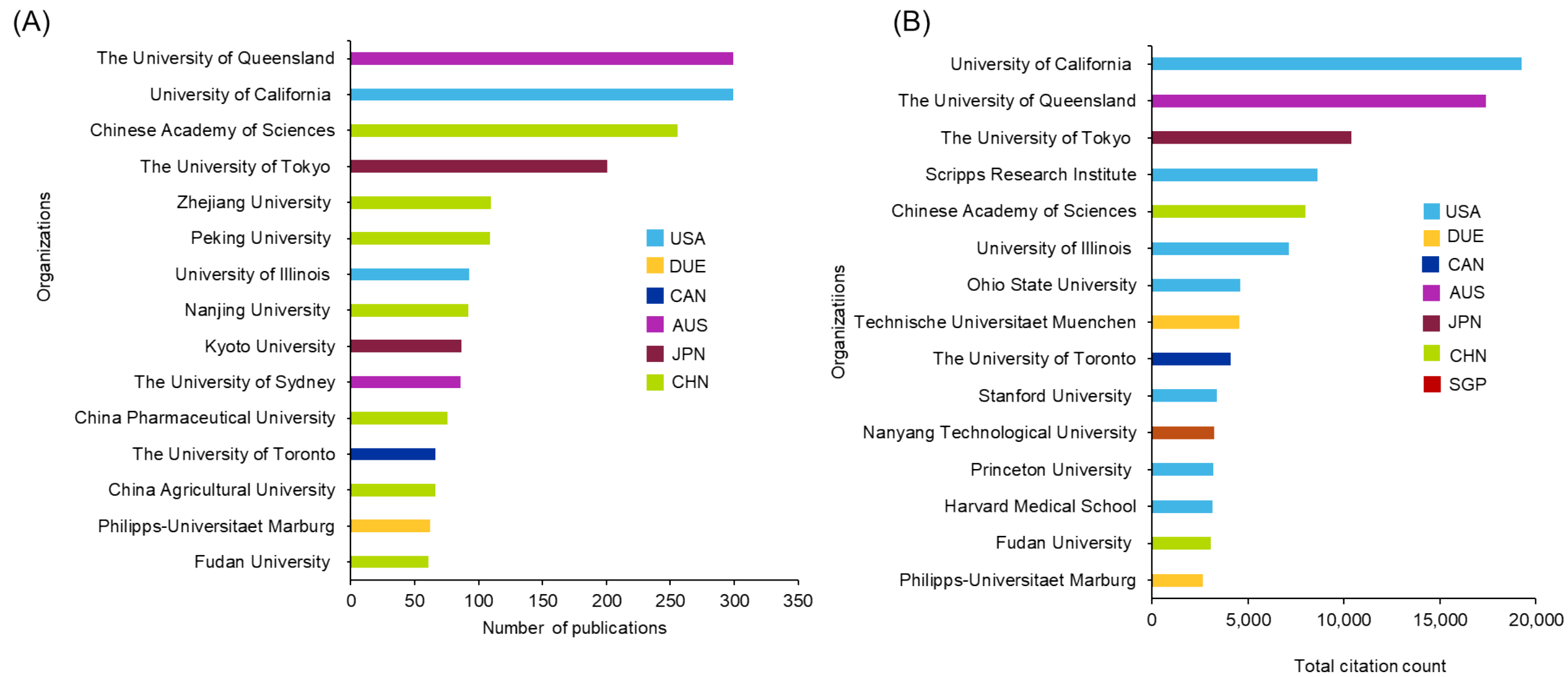

Figure S3: Leading research organizations in the field of cyclic peptides based on (A) publication output and (B) total citations. Data for the period 2006-2025. Source: CAS Content Collection. Standard three letter abbreviations used to represent countries: USA, United States of America; DUE, Germany; CAN, Canada; AUS, Australia; JPN, Japan; CHN, China, SGP, Singapore.

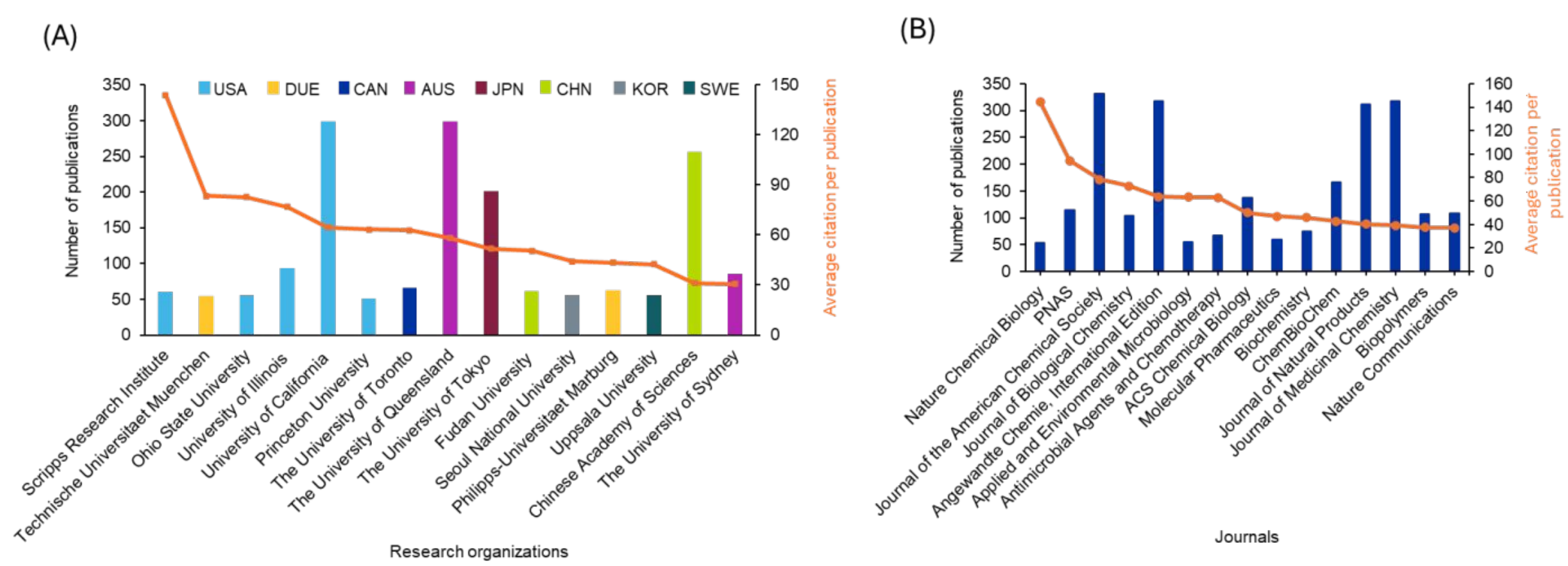

Figure S4: Leading (A) research organizations and (B) scientific journals in the field of cyclic peptides based on journal publication and citation data for the period 2006-2025. Source: CAS Content Collection. Abbreviations used: PNAS, Proceedings of the National Academy of Sciences; USA, United States of America; DUE, Germany; CAN, Canada; AUS, Australia; JPN, Japan; CHN, China; KOR, South Korea; SWE, Sweden.

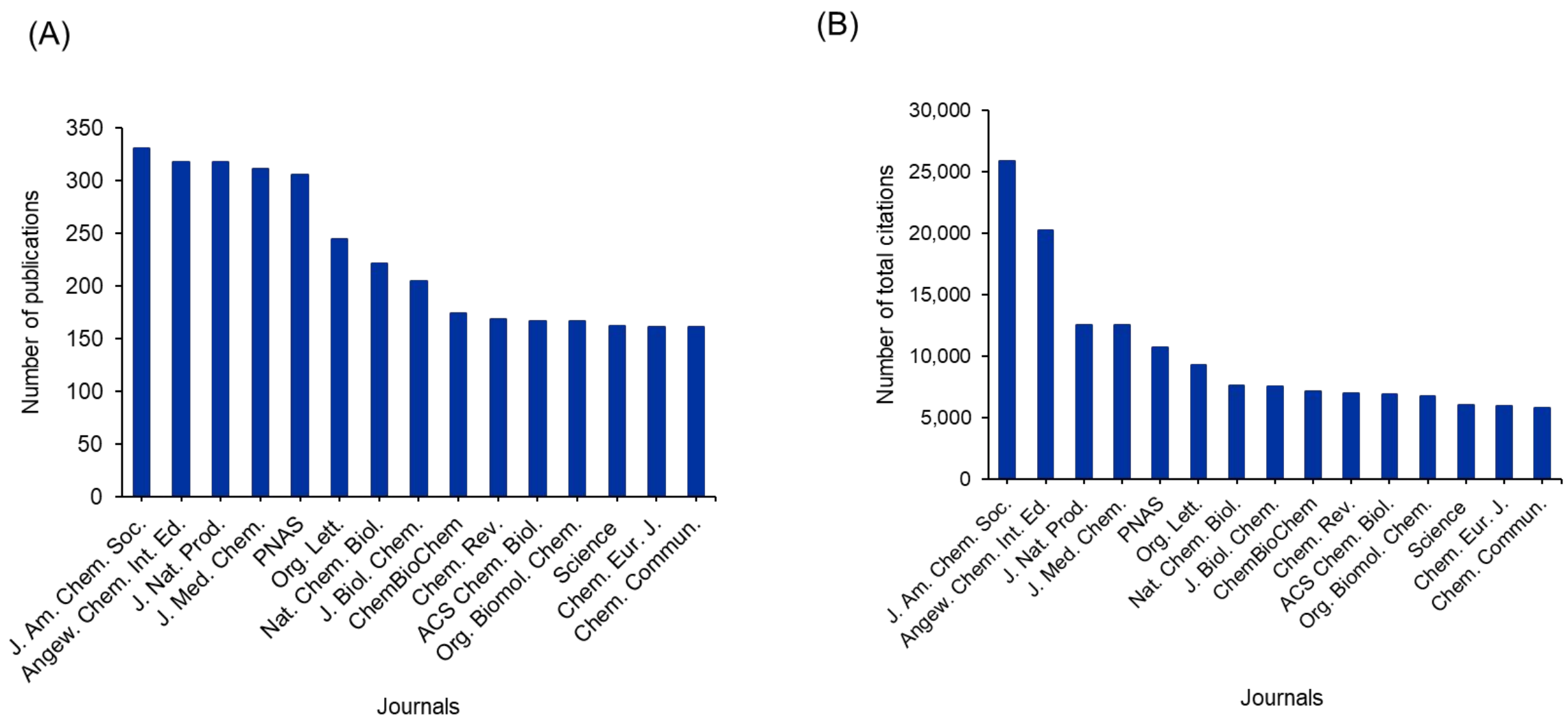

Figure S5: Most influential journals in cyclic peptide research based on their (A) number of publications and (B) citation count. Data includes journal publications related to cyclic peptides for the period 2006-2025. Source: CAS Content Collection. Abbreviations used: JACS, Journal of the American Chemical Society; Angew. Chem. Int. Ed., Angewandte Chemie, International Edition; J. Nat. Prod., Journal of Natural Products; J. Med. Chem., Journal of Medicinal Chemistry; PNAS; Proceedings of the National Academy of Sciences; Org. Lett., Organic Letters; Nat. Chem. Biol., Nature Chemical Biology; J. Biol. Chem., Journal of Biological Chemistry; Chem. Rev., Chemical Reviews; ACS Chem. Biol., ACS Chemical Biology; Org. Biomol. Chem., Organic and Biomolecular Chemistry; Chem. Eur. J., Chemistry - A European Journal; Chem. Commun., Chemical Communications.

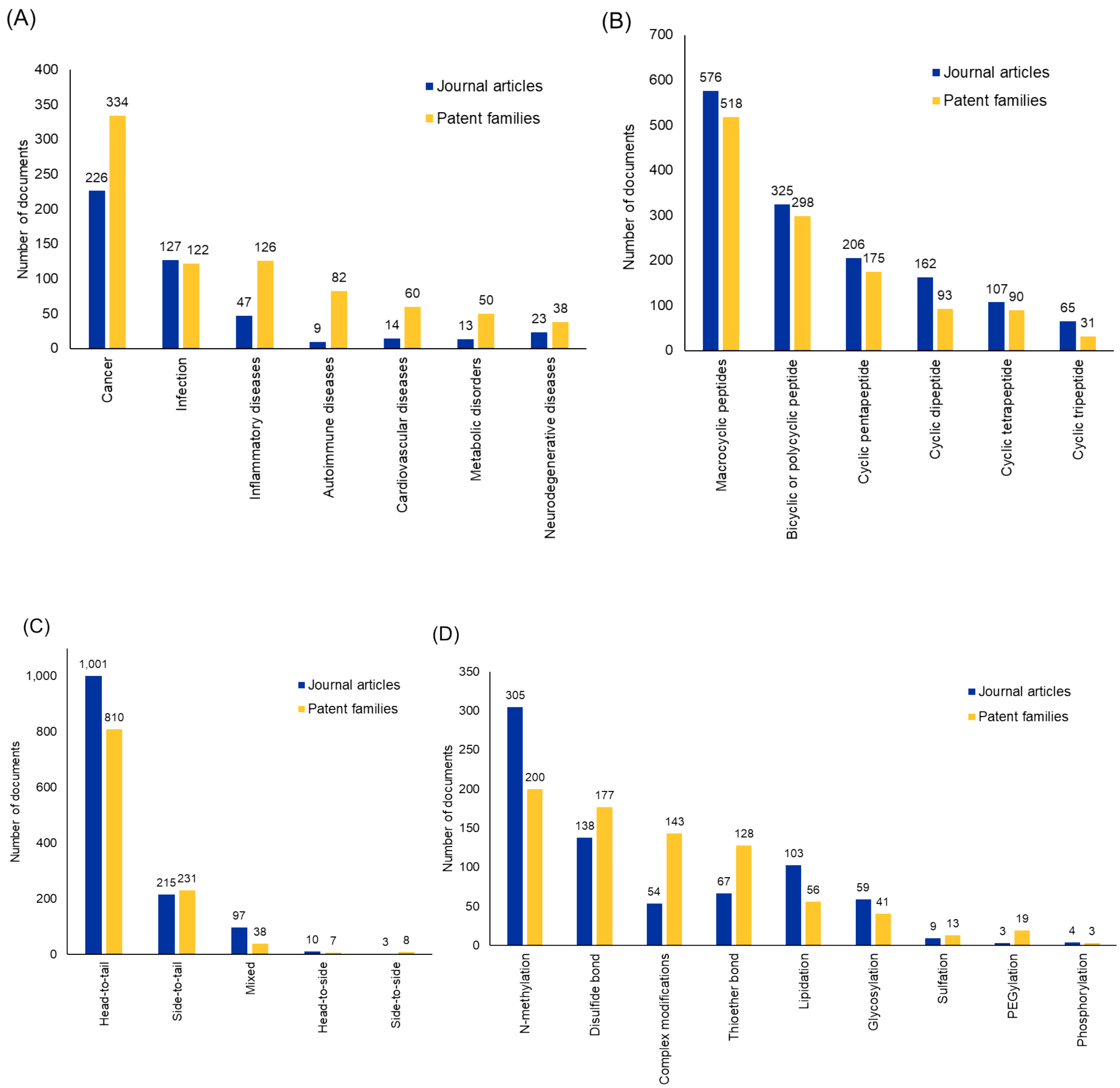

Figure S6: Number of documents contributing to CAS-indexed cyclic peptides, categorized by (A) therapeutic areas, (B) cyclic peptide type, (C) cyclization type, and (D) modifications. Only cyclic peptides indexed with CAS roles THU, or PAC, or PKT covering the period 2020-2025 were included for the analysis. Source: CAS Content Collection.

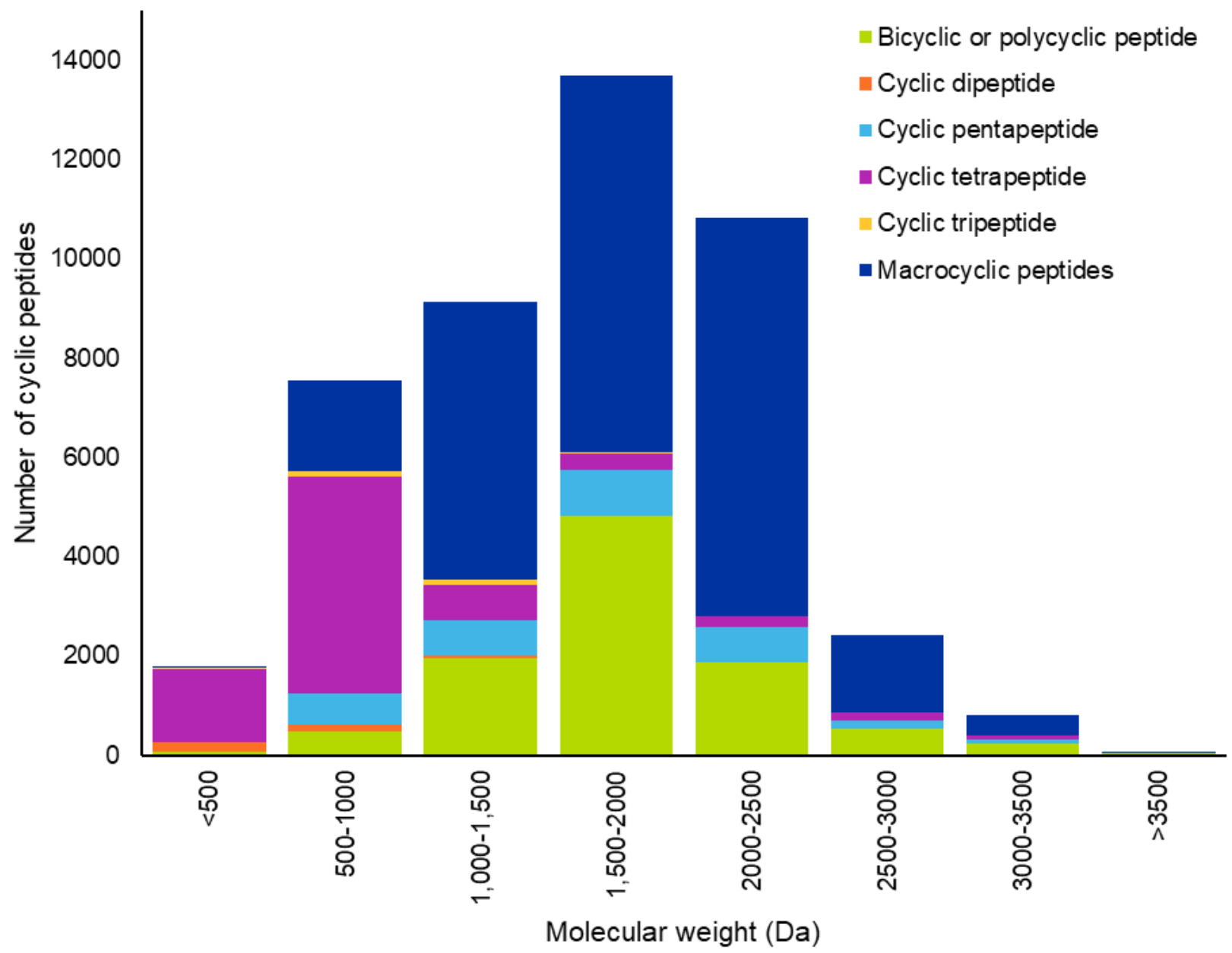

Figure S7: Distribution of various types of cyclic peptides according to their molecular weight. Only cyclic peptides indexed with CAS roles THU, or PAC, or PKT covering the period 2020-2025 were included for the analysis. Source: CAS Content Collection.

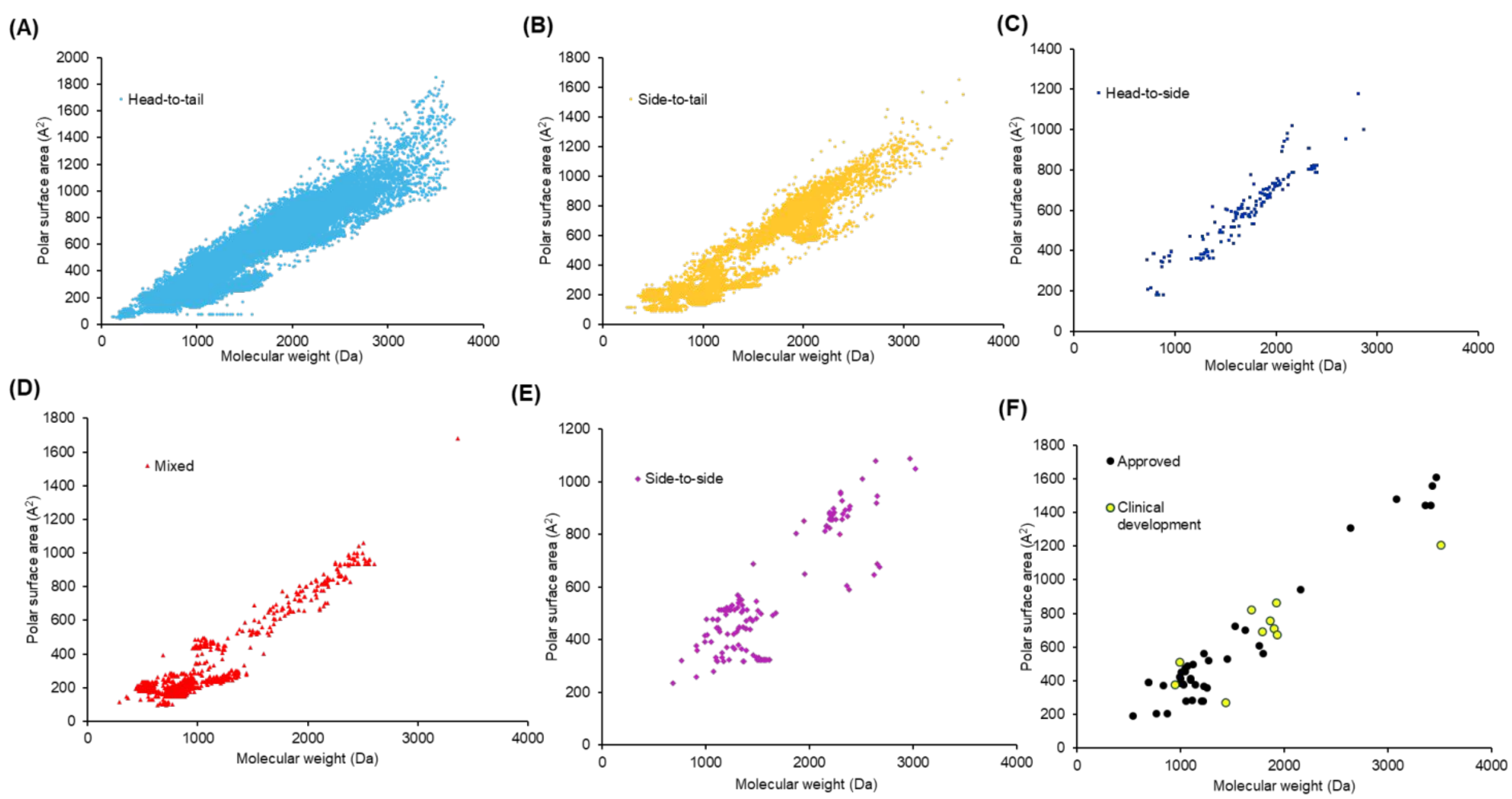

Figure S8: Scatter plots showing the relationship between molecular weight and polar surface area for cyclic peptides according to each cyclization type: (A) Head-to-tail (B) side-to-tail (C) head-to-side (D) mixed (E) side-to-side, and (F) overlay of clinically approved drugs and candidate in clinical development

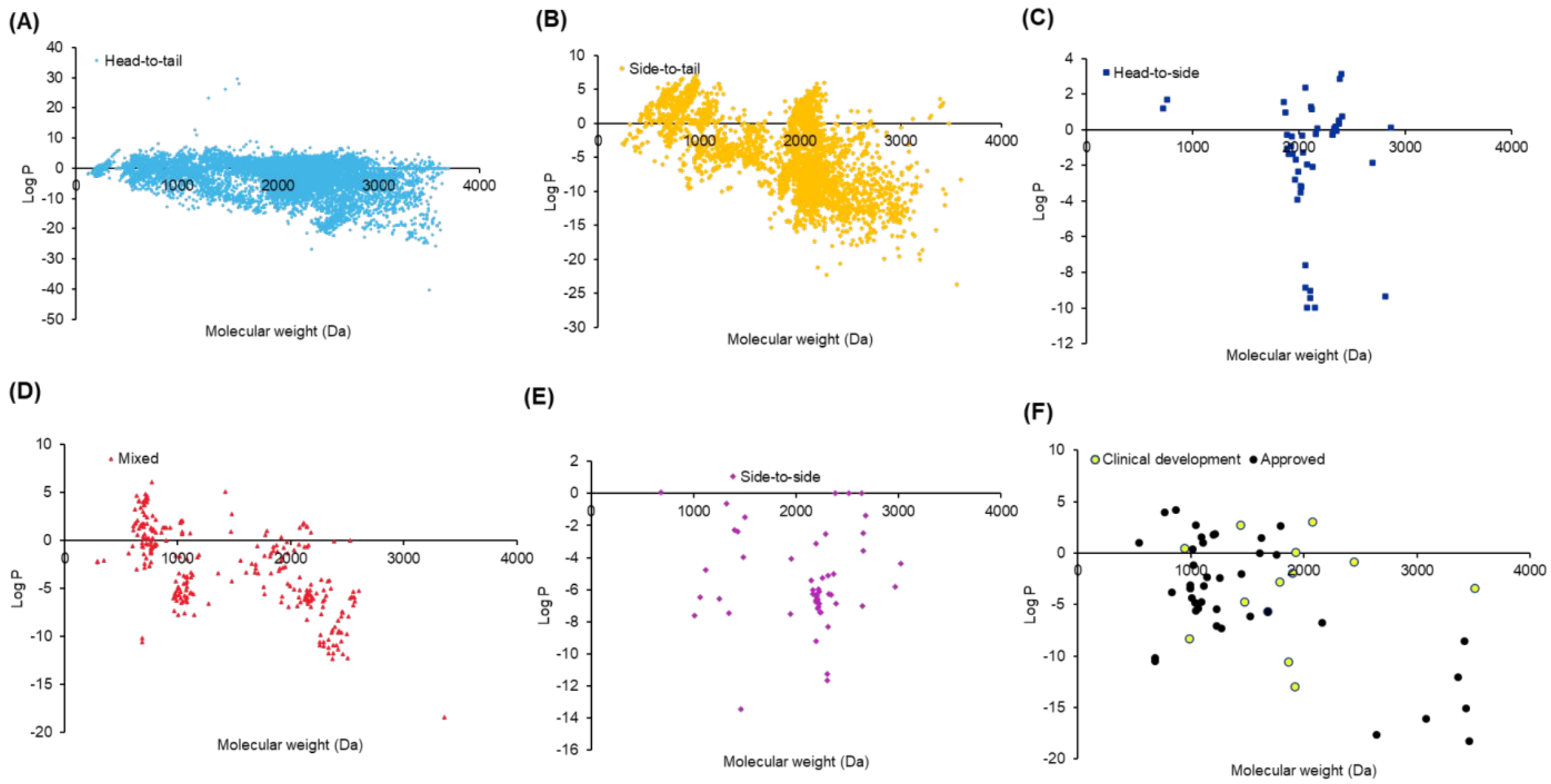

Figure S9: Scatter plots showing the relationship between molecular weight and Log P for cyclic peptides according to each cyclization type: (A) Head-to-tail (B) Side-to-tail (C) Head-to-side (D) Mixed (E) Side-to-side (F) overlay of clinically approved drugs and candidate in clinical development

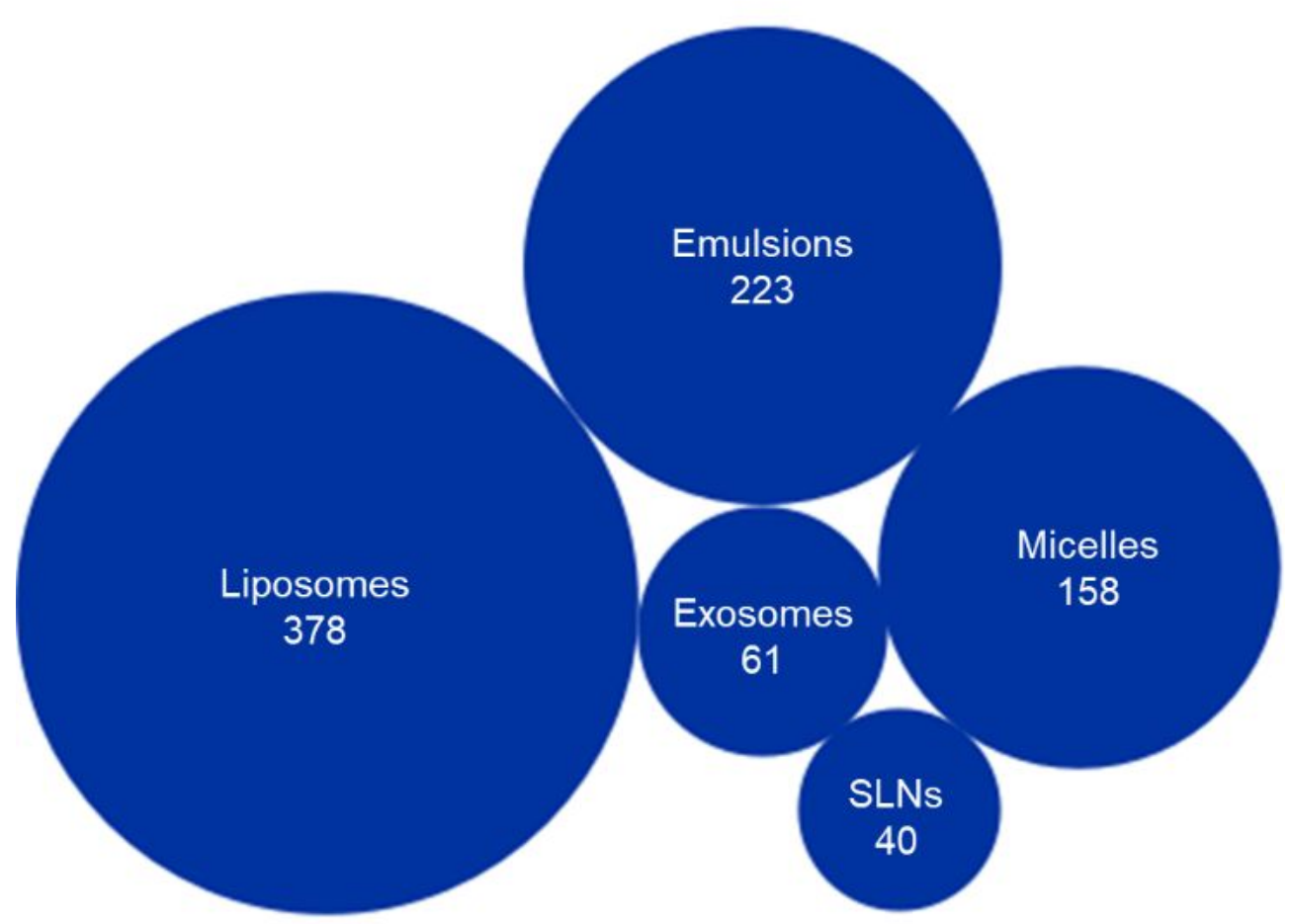

Figure S10: Number of documents associated with various delivery systems for cyclic peptides. The size of the circles reflects the number of documents. Data for the period 2006-2025. Source: CAS Content Collection.

Table S1: Distribution of cyclic peptide types across patents from leading commercial assignees

Commercial patent assignee	Macrocyclic peptides	Bicyclic or polycyclic peptide	Cyclic tetrapeptide	Cyclic pentapeptide	Cyclic dipeptide	Cyclic tripeptide
Bicycle Therapeutics	38	3,411	0	0	0	0
Bristol-Myers Squibb	7,200	60	0	0	1	0
Merck	2,295	392	4	3	2	0
Roche	0	0	0	0	0	2
Enanta Pharmaceuticals	0	6	0	0	0	0
Novartis	166	7	173	310	0	0
Entrada Therapeutics	355	55	0	4	0	0
Hybio Pharmaceutical	2	1	0	0	0	0

Table S2: Distribution of cyclization types across patents from leading commercial assignees

Commercial patent assignee	Head-to-tail	Side-to-tail	Mixed	Head-to-side	Side-to-side
Bicycle Therapeutics	904	2,543	0	0	2
Bristol-Myers Squibb	7,246	1	0	13	1
Merck	1,702	991	2	1	0
Roche	0	2	0	0	0
Enanta Pharmaceuticals	0	6	0	0	0
Novartis	128	490	0	38	0
Entrada Therapeutics	361	1	44	0	8
Hybio Pharmaceutical	3	0	0	0	0

Table S3: Distribution of cyclic peptide modifications across patents from leading commercial assignees

Commercial patent assignee	N-methylation	Disulfide bond	Thioether bond	Complex modifications	Lipidation	Glycosylation	PEGylation	Sulfation
Bicycle Therapeutics	0	0	1,417	41	1	1	0	0
Bristol-Myers Squibb	0	0	485	516	0	0	0	0
Merck	282	33	249	222	13	1	0	0
Roche	48	0	0	3	2	0	0	0
Enanta Pharmaceuticals	3	0	0	0	0	0	0	0
Novartis	453	2	1	102	0	1	0	0
Entrada Therapeutics	0	4	1	2	0	0	46	1
Hybio Pharmaceutical	1	4	1	0	1	0	0	1

Table S4: Highlighted regulatory approved cyclic peptide therapeutics. Source: CAS Content Collection

Approval year	Cyclic peptide therapeutic	CAS RN	Target	Indication	Molar mass (g/mol)	Delivery route	Lipidation	Glycosylation	Thioether bond	Disulfide bond	Company, location
2023	Rezafungin (Rezzayo)	1396640-59-7	β-(1,3)-D-glucan synthase	Invasive candidiasis and candidemia	1,285.44	IV	yes	no	No	no	Cidara Therapeutics, USA
2023	Motixafortide (Aphexda)	664334-36-5	CXCR4	Multiple myeloma	2,159.60	SC	no	no	no	yes	BioLineRx, Israel
2023	Zilucoplan (Zilbrysq)	1841136-73-9	Complement C5	Generalized myasthenia gravis	3,562.23	SC	yes	no	no	yes	UCB, Belgium
2021	Pegcetacoplan (Empaveli)	2019171-69-6	Complement C3	Paroxysmal nocturnal hemoglobinuria	43,520.10	SC, IV	no	no	no	no	Apellis Pharmaceuticals, USA
2021	Vosoritide (Voxzogo)	1480724-61-5	Natriuretic peptide receptor B (NPR-B)	Achondroplasia	4,102.78	SC	no	no	no	no	BioMarin Pharmaceuticals, USA
2021	Voclosporin (Lupkynis)	515814-01-4	Calcineurin	Lupus nephritis	1,214.65	Oral	no	no	no	no	Aurinia Pharmaceuticals, Canada
2020	Setmelanotide (Imcivree)	920014-72-8	Melanocortin-4 receptor (MC4R)	Obesity (genetic)	1,117.32	SC	no	no	no	yes	Ipsen, France
2019	Bremelanotide (Vyleesi)	189691-06-3	Melanocortin receptors (MC3R/MC4R)	Female sexual dysfunction	1,025.18	SC	no	no	no	no	AMAG Pharmaceuticals, USA
2018	177Lu-DOTATATE (Lutathera)	437608-50-9	Somatostatin receptors	Gastroenteropancreatic neuroendocrine tumors	1,607.60	IV	no	no	no	yes	Novartis, Switzerland
2018	Plitidepsin (Aplidin)	137219-37-5	eEF1A2 (eukaryotic	Multiple myeloma	1,110.36	IV	no	no	no	no	PharmaMar, Spain

Approval year	Cyclic peptide therapeutic	CAS RN	Target	Indication	Molar mass (g/mol)	Delivery route	Lipidation	Glycosylation	Thioether bond	Disulfide bond	Company, location
			elongation factor 1A2)								
2017	Plecanatide (Trulance)	467426-54-6	Guanylate cyclase-C (GC-C)	Chronic constipation, IBS-C	1,681.89	Oral	no	no	no	yes	Bausch Health, Canada
2017	Voxilaprevir (Vosevi)	1535212-07-7	HCV NS3/4A protease	Chronic hepatitis C	868.90	Oral	no	no	no	no	Gilead Sciences, USA
2016	Grazoprevir (Zepatier)	1350514-68-9	HCV NS3/4A protease	Chronic hepatitis C virus genotype 1 or 4	766.90	Oral	no	no	no	no	Merck, USA
2014	Vasopressin (Vasostrict)	11000-17-2	Vasopressin receptors (V1A, V1B, V2)	Diabetes insipidus, vasodilatory shock	1,084.24	IV, IM, SC	no	no	no	yes	Par Pharmaceutical, USA
2014	Oritavancin (Orbactiv)	171099-57-3	Bacterial cell wall synthesis (D-Ala-D-Ala)	Skin and soft tissue infections	1,793.12	IV	yes	yes - 3 sugars	no	no	The Medicines Company, USA
2014	Dalbavancin (Dalvance)	171500-79-1	Bacterial cell wall synthesis (D-Ala-D-Ala)	Skin and soft tissue infections	1,816.71	IV	yes	yes - 2 sugars	no	no	Durata Therapeutics/AbbVie, USA
2012	Terlipressin (Glypressin)	14636-12-5	Vasopressin receptors (V1A)	Hepatorenal syndrome	1,227.38	IV	no	no	no	yes	Ferring Pharmaceuticals, Switzerland
2012	Linaclotide (Linzess)	851199-59-2	Guanylate cyclase-C (GC-C)	Irritable bowel syndrome, constipation	1,526.73	Oral	no	no	no	yes	Allergan/AbbVie, USA
2012	Pasireotide (Signifor)	396091-73-9	Somatostatin receptors (SSTR1-5)	Cushing's disease, acromegaly	1,047.23	SC, IM	no	no	no	yes	Novartis, Switzerland
2009	Romidepsin (Istodax)	128517-07-7	Histone deacetylases (HDAC1, HDAC2)	T-cell lymphoma	540.69	IV	no	no	yes	no	Celgene/Bristol Myers Squibb, USA

Approval year	Cyclic peptide therapeutic	CAS RN	Target	Indication	Molar mass (g/mol)	Delivery route	Lipidation	Glycosylation	Thioether bond	Disulfide bond	Company, location
2009	Telavancin (Vibativ)	372151-71-8	Bacterial cell wall synthesis (D-Ala-D-Ala)	Skin and soft tissue infections	1,755.65	IV	yes	yes - 2 sugars	no	no	Cumberland Pharmaceuticals, USA
2007	Lanreotide (Somatuline)	108736-35-2	Somatostatin receptors (SSTR2, SSTR5)	Acromegaly, neuroendocrine tumors	1,096.33	IM	no	no	no	yes	Ipsen, France
2006	Anidulafungin (Eraxis)	166663-25-8	β-(1,3)-D-glucan synthase	Invasive candidiasis	1,140.25	IV	yes	no	no	no	Pfizer, USA
2005	Pramlintide (Symlin)	151126-32-8	Amylin receptors	Diabetes	3,949.44	SC	no	no	no	yes	AstraZeneca, UK
2004	Ziconotide (Prialt)	107452-89-1	N-type voltage-gated calcium channels (Cav2.2)	Severe chronic pain	2,639.14	Intrathecal	no	no	no	yes	Jazz Pharmaceuticals, Ireland
2003	Daptomycin (Cubicin)	103060-53-3	Bacterial cell membrane	Gram-positive bacterial infections	1,620.69	IV	yes	no	no	no	Merck, USA
2002	Micafungin (Mycamine)	235114-32-6	β-(1,3)-D-glucan synthase	Invasive candidiasis	1,270.28	IV	yes	no	no	no	Astellas Pharma, Japan
2001	Nesiritide (Natrecor)	124584-08-3	Natriuretic peptide receptor A (NPR-A)	Acute heart failure	3,464.07	IV	no	no	no	yes	Johnson & Johnson, USA
2001	Caspofungin (Cancidas)	162808-62-0	β-(1,3)-D-glucan synthase	Invasive fungal infections	1,093.33	IV	yes	no	no	no	Merck, USA
2000	Atosiban (Tractocile)	90779-69-4	Oxytocin and vasopressin V1A receptors	Preterm labor	994.19	IV	no	no	no	no	Ferring Pharmaceuticals, Switzerland
1998	Eptifibatide (Integrilin)	188627-80-7	Glycoprotein IIb/IIIa receptor	Acute coronary syndromes	831.97	IV	no	no	no	no	Millennium Pharmaceuticals, USA

Approval year	Cyclic peptide therapeutic	CAS RN	Target	Indication	Molar mass (g/mol)	Delivery route	Lipidation	Glycosylation	Thioether bond	Disulfide bond	Company, location
1997	Carbetocin (Duratocin)	37025-55-1	Oxytocin receptors	Postpartum hemorrhage	998.17	IV, IM	no	no	no	yes	Ferring Pharmaceuticals, Switzerland
1995	Carperitide (Hanp)	89213-87-6	Natriuretic peptide receptor A (NPR-A)	Acute heart failure	3,080.48	IV	no	no	no	yes	Daiichi Sankyo, Japan
1988 / 2020	Octreotide (Sanostatine)	83150-76-9	Somatostatin receptors (SSTR2, SSTR5)	Acromegaly, neuroendocrine tumors	1,019.25	SC, IM (1988), Oral (2020)	no	no	no	yes	Novartis, Switzerland
1987	Teicoplanin (Targocid)	61036-62-2	Bacterial cell wall synthesis (D-Ala-D-Ala)	Gram-positive bacterial infections	1,879.67	IV, IM	yes	yes - 3 sugars	no	no	Sanofi, France
1986	Human Calcitonin (Cibacalcin)	21215-62-3	Calcitonin receptors	Osteoporosis, Paget's disease	3,417.88	SC, Nasal spray	no	no	no	yes	Novartis, Switzerland
1983	Cyclosporine (Neoral/Sandimmune)	59865-13-3	Calcineurin	Immunosuppression, organ transplant	1,202.64	Oral, IV	no	no	yes	no	Novartis, Switzerland
1981	Elcatonin (Elcitonin)	60731-46-6	Calcitonin receptors	Osteoporosis, Paget's disease	3,363.83	SC, IM	no	no	no	yes	Asahi Kasei Pharma, Japan
1980	Oxytocin (Pitocin)	50-56-6	Oxytocin receptors	Labor induction, postpartum hemorrhage	1,007.19	IV, IM	no	no	no	yes	Par Pharmaceutical, USA
1978	Salmon Calcitonin (Miacalcin)	47931-85-1	Calcitonin receptors	Osteoporosis, Paget's disease	3,431.90	SC, Nasal spray	no	no	no	yes	Novartis, Switzerland
1978	Desmopressin (DDAVP)	16679-58-6	Vasopressin V2 receptors	Diabetes insipidus, bedwetting	1,069.22	Nasal spray, Oral, SC	no	no	no	yes	Ferring Pharmaceuticals, Switzerland
1974	Enviomycin (Tuberactin)	33103-22-9	Bacterial 70S ribosome	Tuberculosis	685.70	IM	no	no	yes	no	Asahi Kasei Pharma, Japan

Approval year	Cyclic peptide therapeutic	CAS RN	Target	Indication	Molar mass (g/mol)	Delivery route	Lipidation	Glycosylation	Thioether bond	Disulfide bond	Company, location
1971	Demoxytocin (Sandopart)	113-78-0	Oxytocin receptors	Labor induction, postpartum mastitis	992.18	Buccal	no	no	no	yes	Sandoz, Switzerland
1971	Ornipressin (POR-8)	3397-23-7	Vasopressin V1A receptors	Esophageal varices bleeding	1,042.20	IV	no	no	no	yes	Ferring Pharmaceuticals, Switzerland
1971	Capreomycin (Capastat)	11003-38-6	Bacterial 70S ribosome	Tuberculosis	1,321.44	IM	no	no	no	no	Akorn, USA
1969	Felypressin (Octapressin)	56-59-7	Vasopressin V1A receptors	Local anesthetic vasoconstrictor	1,040.23	Local injection	no	no	no	no	Dentsply Sirona, USA
1964	Dactinomycin (Cosmegen)	50-76-0	DNA transcription	Renal Wilms' tumor	1,255.44	IV	no	no	no	no	Merck, USA
1962	Colistin (Coly-Mycin)	1066-17-7	Bacterial cell membrane	Multidrug-resistant gram-negative infections	1,155.46	IV, Inhalation	yes	no	no	no	Hospira, USA
1953	Viomycin (Viocin)	32988-50-4	Bacterial 70S ribosome	Multidrug-resistant gram-positive infections	685.69	IV, Oral	no	no	no	no	Pfizer, USA
1958	Vancomycin (Vancocin)	1404-90-6	Bacterial cell wall synthesis (D-Ala-D-Ala)	Gram-positive bacterial infections	1,449.27	IV, Oral	no	yes - 2 sugars	no	no	ANI Pharmaceuticals, USA
1951	Polymyxin B (Poly-Rx)	1404-26-8	Bacterial cell membrane	Multidrug-resistant gram-negative infections	1,203.50	IV, Topical	yes	no	no	no	Pfizer, USA
1948	Bacitracin (Baciguent)	1405-87-4	Bacterial cell wall synthesis (undecaprenyl	Bacterial infections	1,422.71	Topical	no	no	no	no	Perrigo, Ireland

Approval year	Cyclic peptide therapeutic	CAS RN	Target	Indication	Molar mass (g/mol)	Delivery route	Lipidation	Glycosylation	Thioether bond	Disulfide bond	Company, location
			pyrophosphate)								

Table S5: SMILE-based analysis (as per methods section) of cyclic peptide therapeutics currently in clinical development

Drug	CAS RN	Peptide type	Cyclization type	Modification
AMY-101 (Compstatin 40)	1427001-89-5	Macrocyclic peptide	Head-to-tail	Disulfide bond, N-methylation
AZP-3813*	n/a	-	-	-
Balixafortide (POL6326)	1051366-32-5	Bicyclic or polycyclic peptide	Head-to-tail	Disulfide bond
BT1718	2227366-66-5	Bicyclic or polycyclic peptide	Head-to-tail	No modification
BT5528*	2648849-70-9	-	-	-
BT7480*	2762739-36-4	-	-	-
CAP-232 (TT-232)	147159-51-1	Cyclic tetrapeptide	Head-to-tail	Disulfide bond
Certepetide (LSTA1)	2580154-02-3	Macrocyclic peptide	Head-to-tail	Disulfide bond
Dolcanatide (SP-333)	1092457-65-2	Bicyclic or polycyclic peptide	Head-to-tail	Disulfide bond
Icotrokinra (JNJ-77242113)	2763602-16-8	Cyclic pentapeptide	Head-to-tail	Disulfide bond, N-methylation
Lonodelestat (POL6014)	906547-89-5	Macrocyclic peptide	Head-to-tail	No modification
MK-0616	2861205-06-1	Bicyclic or polycyclic peptide	Side-to-tail	Lipidation
Noraramtide (BHV-1100)*	2580150-96-3	-	-	-
Paluratide (LUNA18)	2676177-63-0	Macrocyclic peptide	Head-to-tail	N-methylation
PL8177*	n/a	-	-	-
PL9643*	n/a	-	-	-
Rusfertide (PTG-300)	1628323-80-7	Macrocyclic peptide	Side-to-side	Disulfide bond
Solnatide (AP301)	259206-53-6	Macrocyclic peptide	Head-to-tail	Disulfide bond
Sulanemadlin (ALRN-6924)	1451199-98-6	Macrocyclic peptide	Head-to-tail	No modification
VT1021*	2942310-76-9	-	-	-
Zelenectide Pevedotin (BT8009)*	2407446-48-2	-	-	-
Zolucatetide (FOG-001)*	3044032-95-0	-	-	-

* SMILE not available in CAS registry
n/a: Not available

Table S6: Structures and physiochemical properties of selected approved cyclic peptides (2010 onwards). Source: CAS Content Collection.

Drug	CAS RN	H bond acceptors	H bond donors	Freely rotatable bonds	logP	Molecular weight	Molar refractivity (m^3/mol)	Number of atoms	Polar surface area ($Å^2$)	Plasma protein binding	Predicted pKa
Rezafungin (Rezzayo) 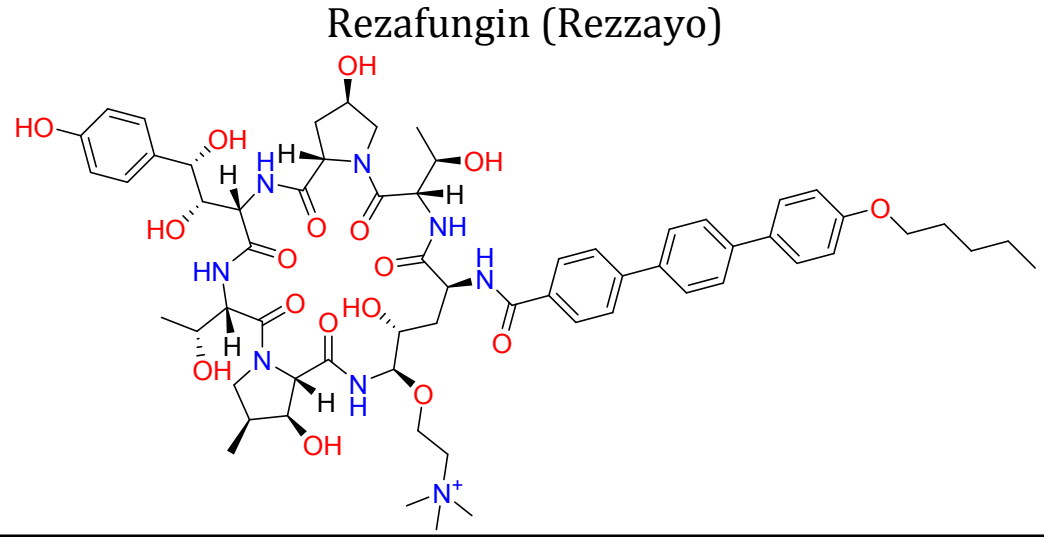	1396640-59-7	25	13	18	-5.447	1226.39	317.38	88	366.42	70.41	9.859±0.26
Motixafortide (Aphexda) 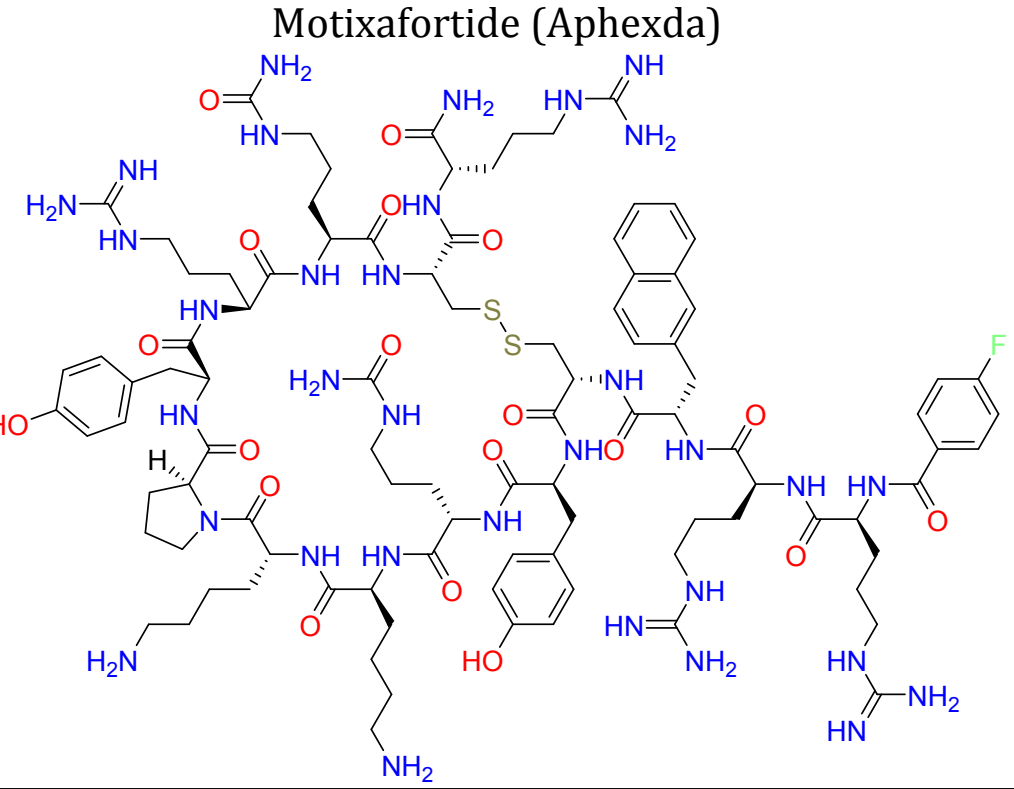	664334-36-5	52	43	53	-6.742	2159.53	560.61	152	942.64	51.03	NR
Zilucoplan (Zilbrysq) 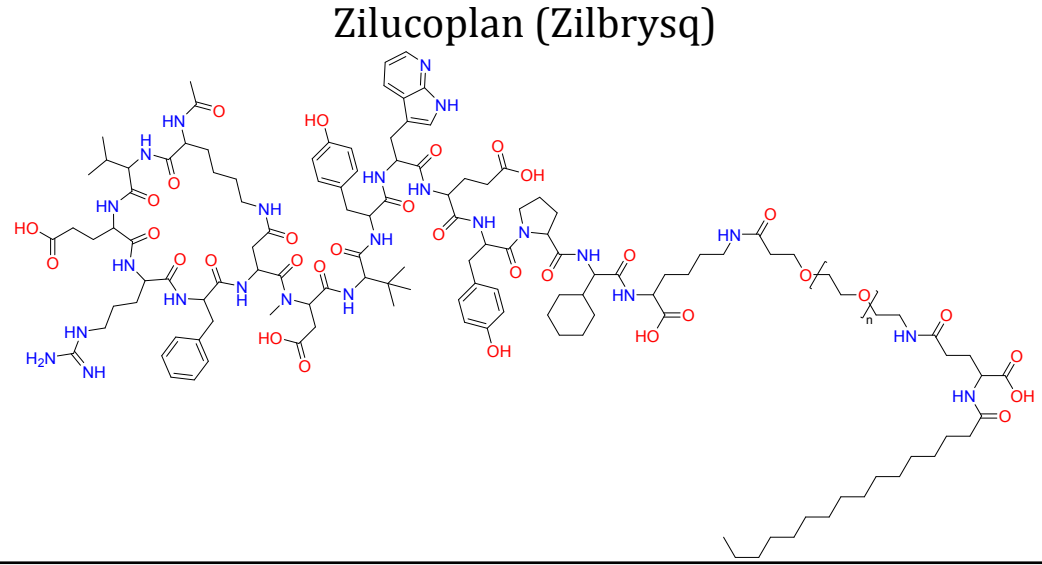	1841136-73-9	NR	NR	NR	NR	NR	NR	NR	NR	100	NR

Voclosporin (Lupkynis) 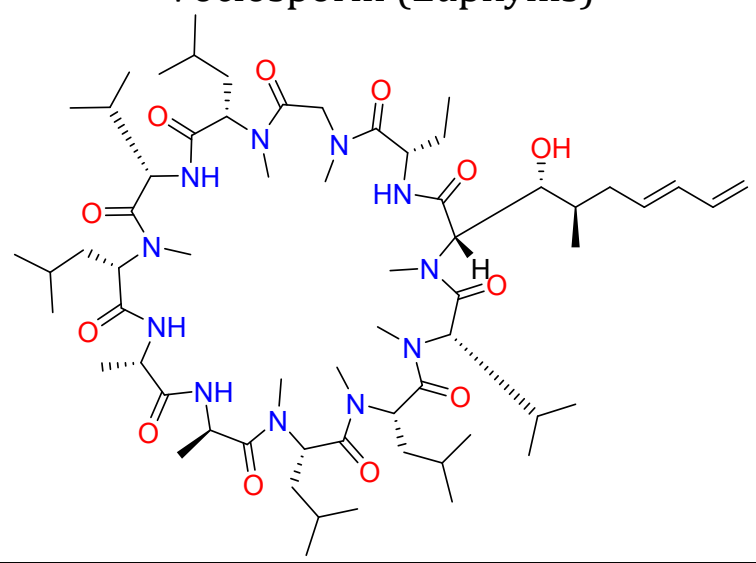	515814-01-4	23	5	16	1.85	1214.62	332.68	86	278.8	100	13.315±0.70
Setmelanotide (Imcivree) 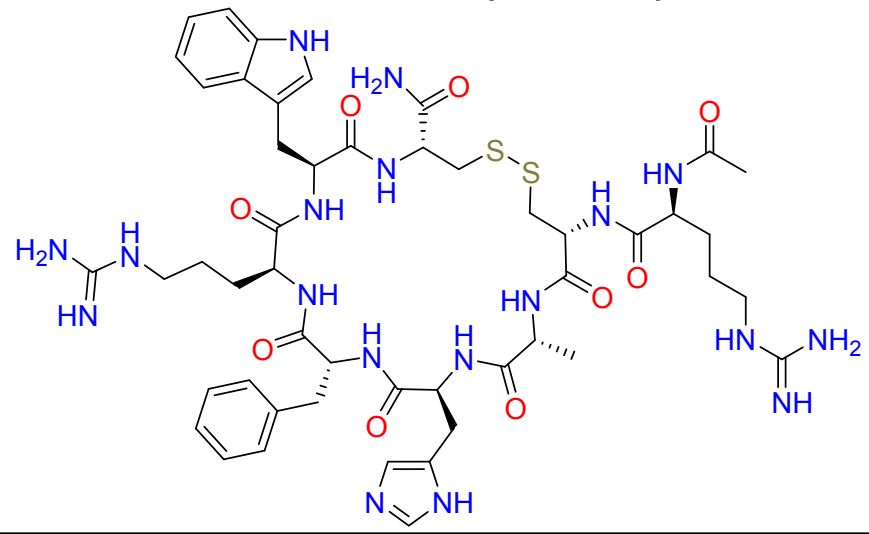	920014-72-8	27	20	20	-3.175	1117.31	290.34	78	497.76	23.15	12.818±0.70
Bremelanotide (Vyleesi) 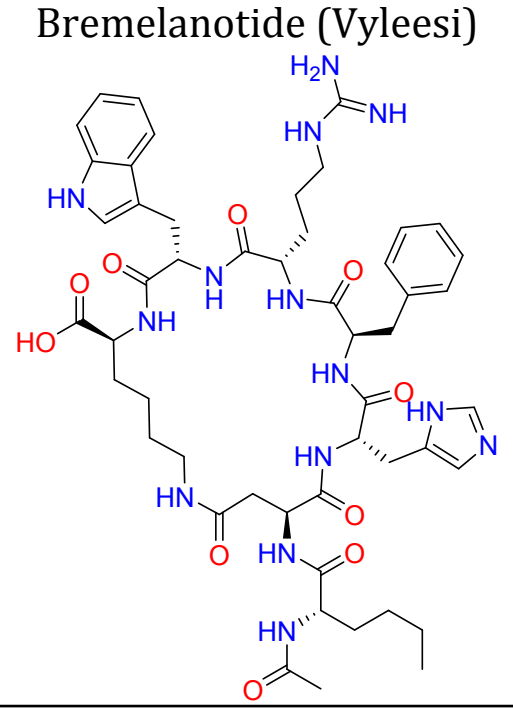	189691-06-3	24	15	18	-1.14	1025.16	268.58	74	376.47	53.24	3.371±0.70
177Lu-DOTATATE (Lutathera) 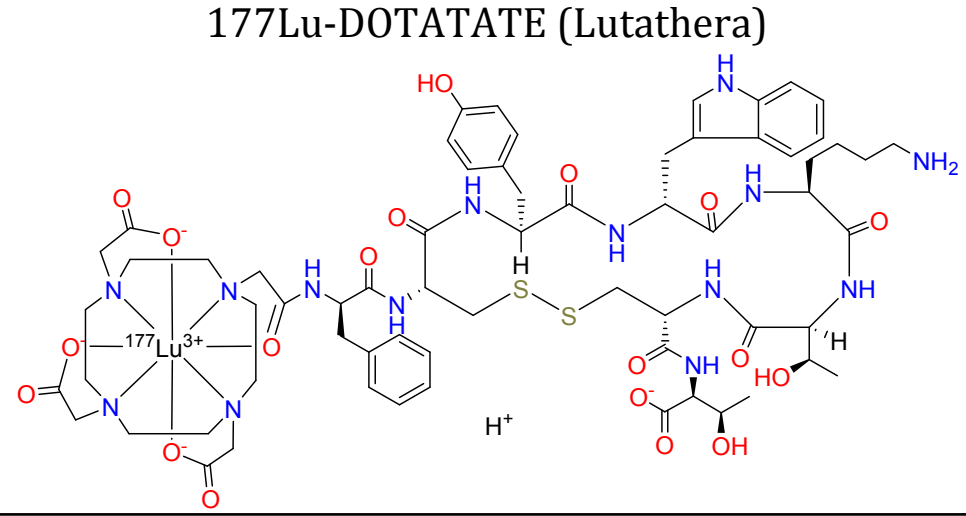	437608-50-9	NR	NR	NR	NR	1609.55	343.19	101	NR	NR	NR

Plitidepsin (Aplidin) 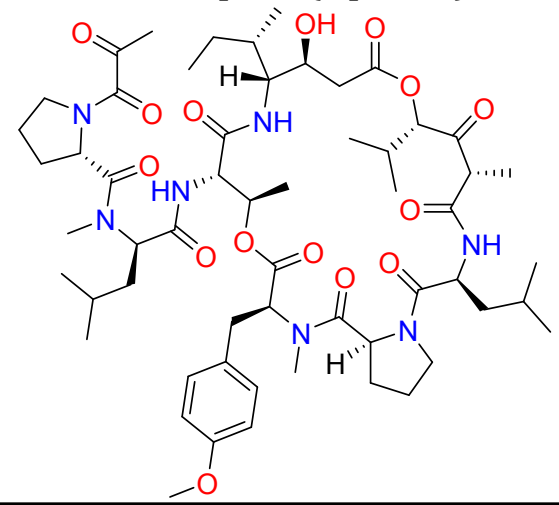	137219-37-5	22	4	15	1.0003	1110.34	288.59	79	284.74	99.94	11.278±0.70
Plecanatide (Trulance) 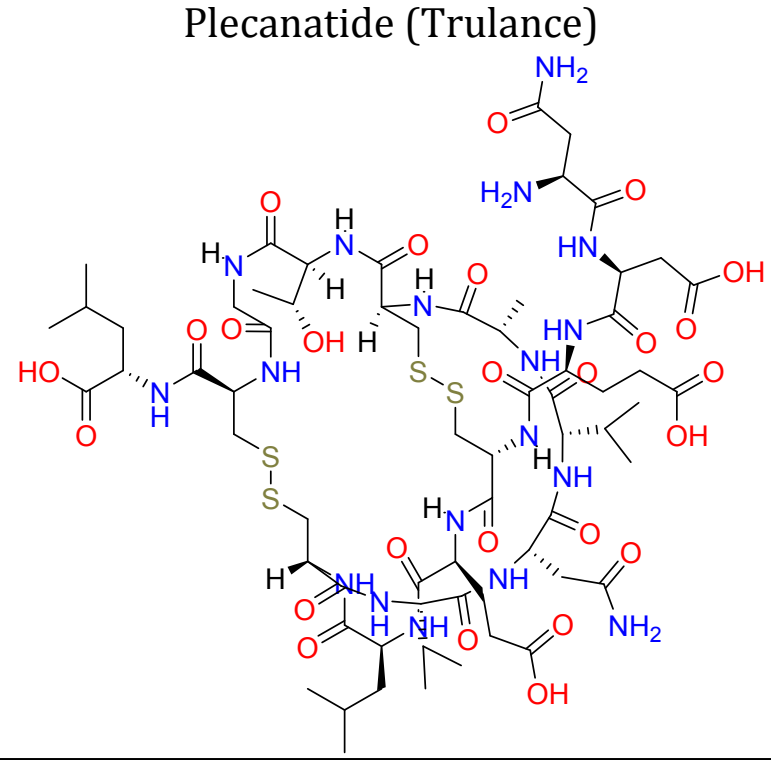	467426-54-6	44	26	28	-5.693	1681.89	398.46	113	819.33	0	3.606±0.21
Voxilaprevir (Vosevi) 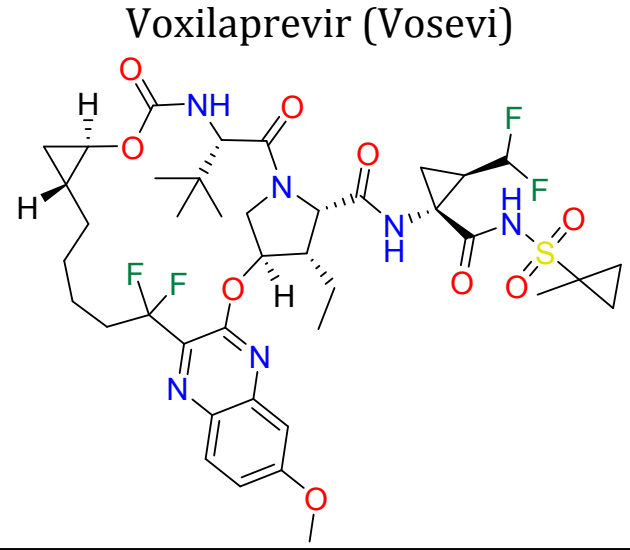	1535212-07-7	15	3	9	4.171	868.94	202.66	60	203.6	95.48	5.485±0.40
Grazoprevir (Zepatier) 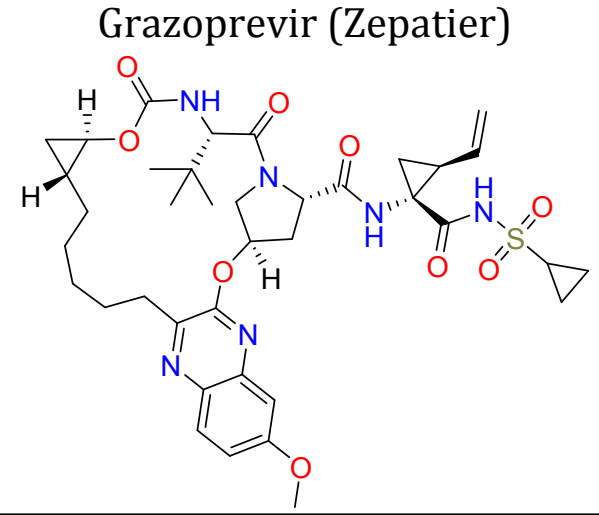	1350514-68-9	15	3	8	3.935	766.91	193.37	54	203.6	73.86	5.511±0.40
Oritavancin (Orbactiv)	171099-57-3	36	22	19	2.651	1793.1	440.43	125	560.98	100	2.929±0.70

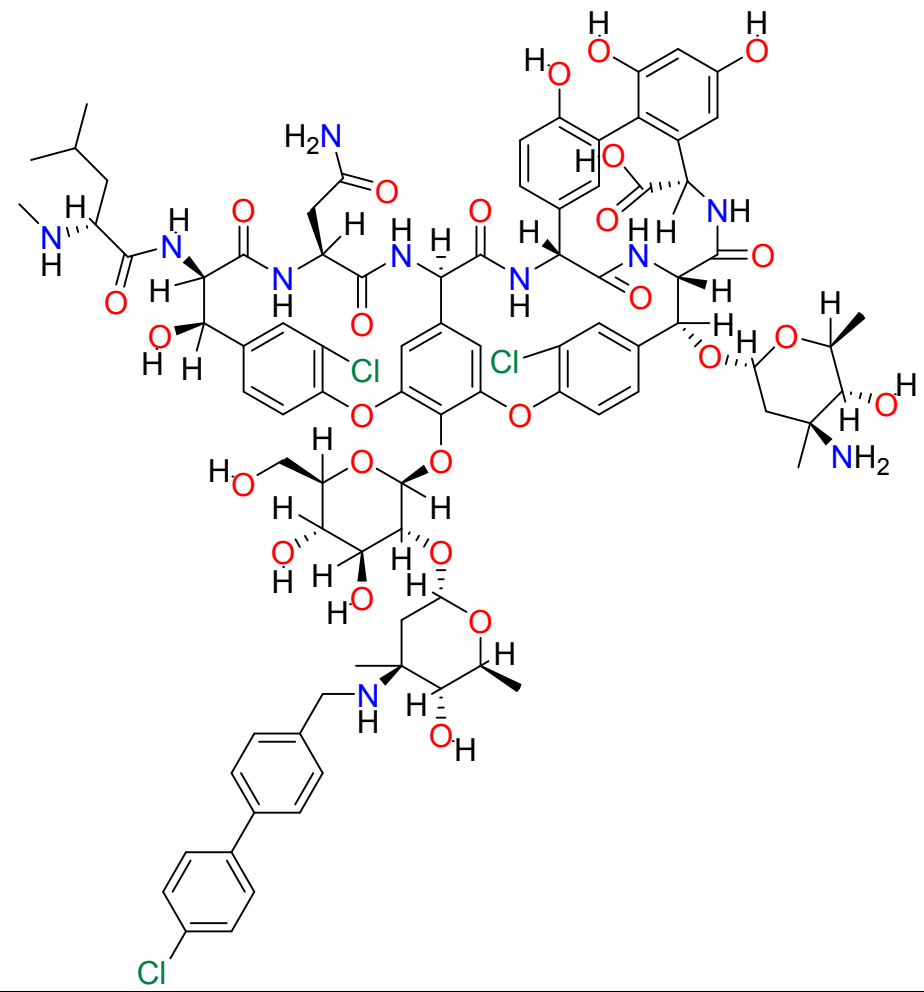											
Terlipressin (Glypressin) 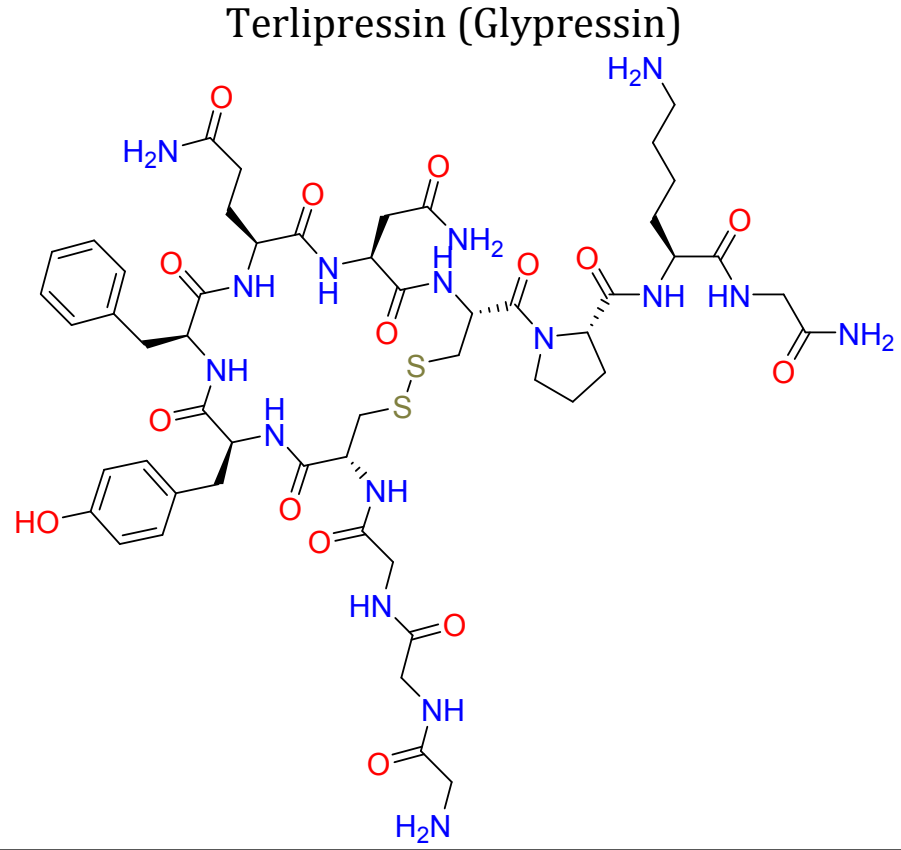	14636-12-5	31	21	25	-7.085	1227.38	305.69	85	563.45	0	9.901±0.15
Linaclotide (Linzess)	851199-59-2	36	21	13	-6.127	1526.75	367.4	101	725.71	0	3.053±0.10

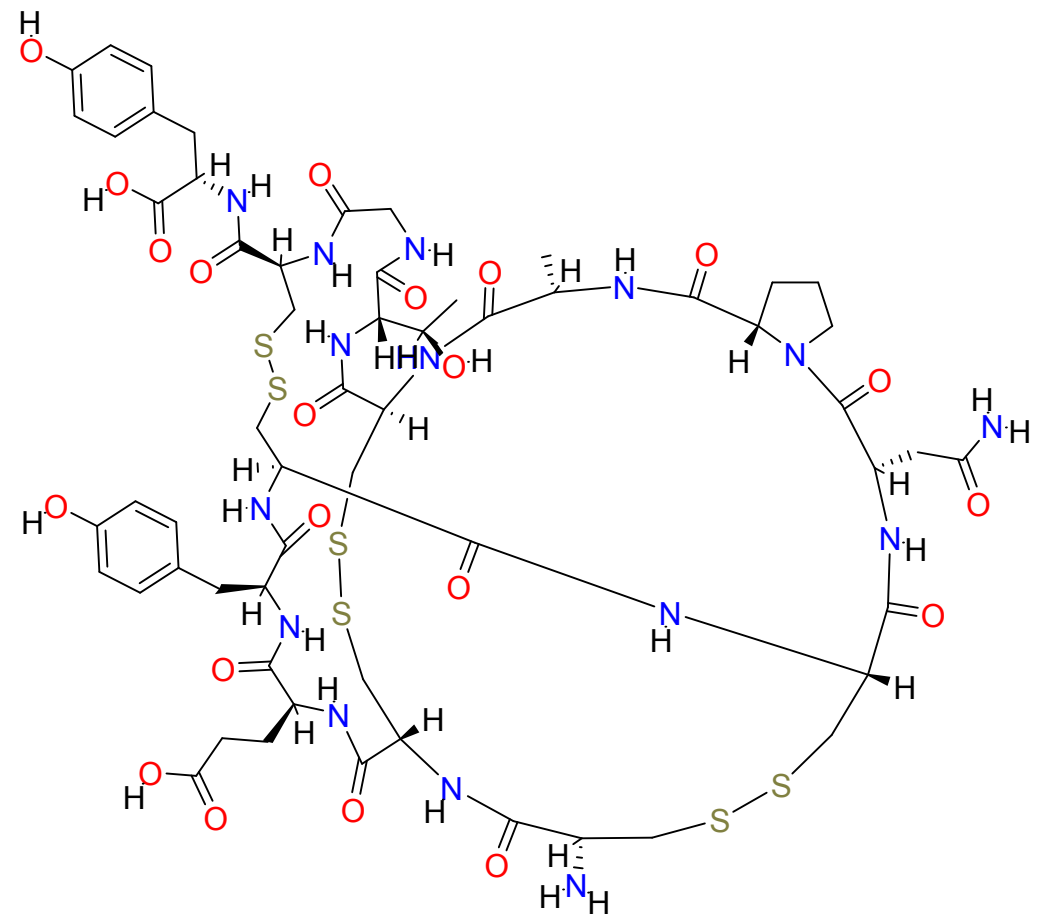											
Pasireotide (Signifor)<br>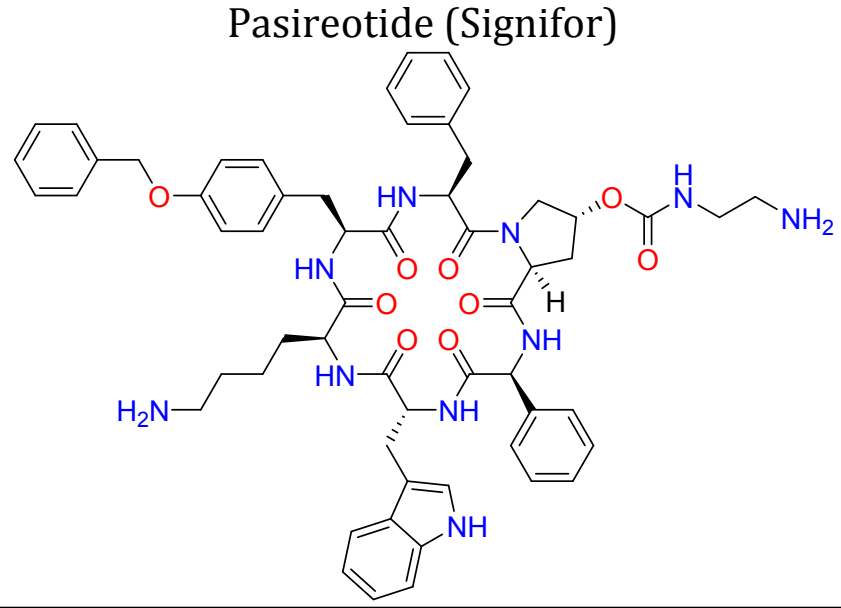	396091-73-9	19	11	18	2.712	1047.21	287.04	77	281.2	87.61	11.858±0.46

Table S7: Structures and physiochemical properties of cyclic peptides in clinical development. Source: CAS Content Collection.

Drug	CAS RN	H bond acceptors	H bond donors	Freely rotatable bonds	logP	Molecular weight	Molar refractivity (m^3/mol)	Number of atoms	Polar surface area $(Å^2)$	Plasma protein binding	Predicted pKa
Rezafungin (Rezzayo) 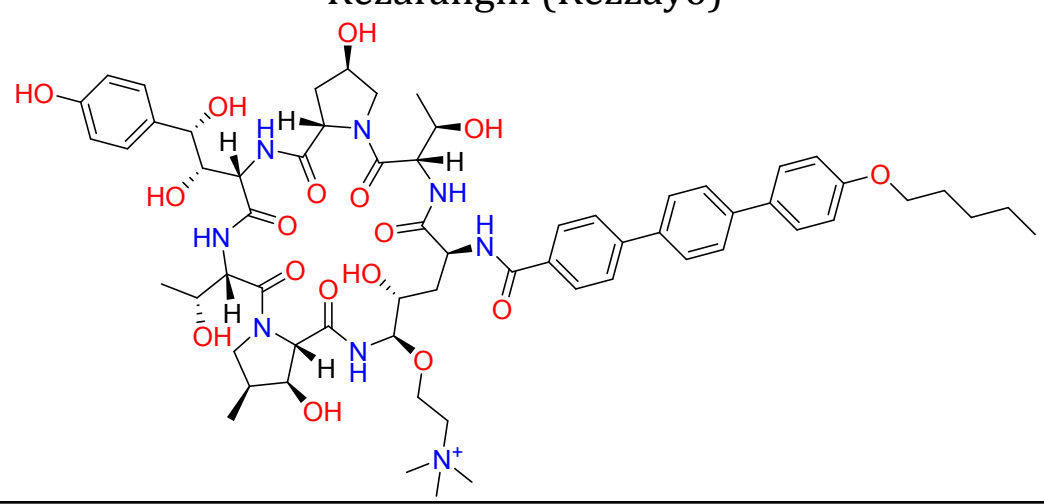	1396640-59-7	25	13	18	-5.447	1226.39	317.38	88	366.42	70.41	9.859±0.26
Motixafortide (Aphexda) 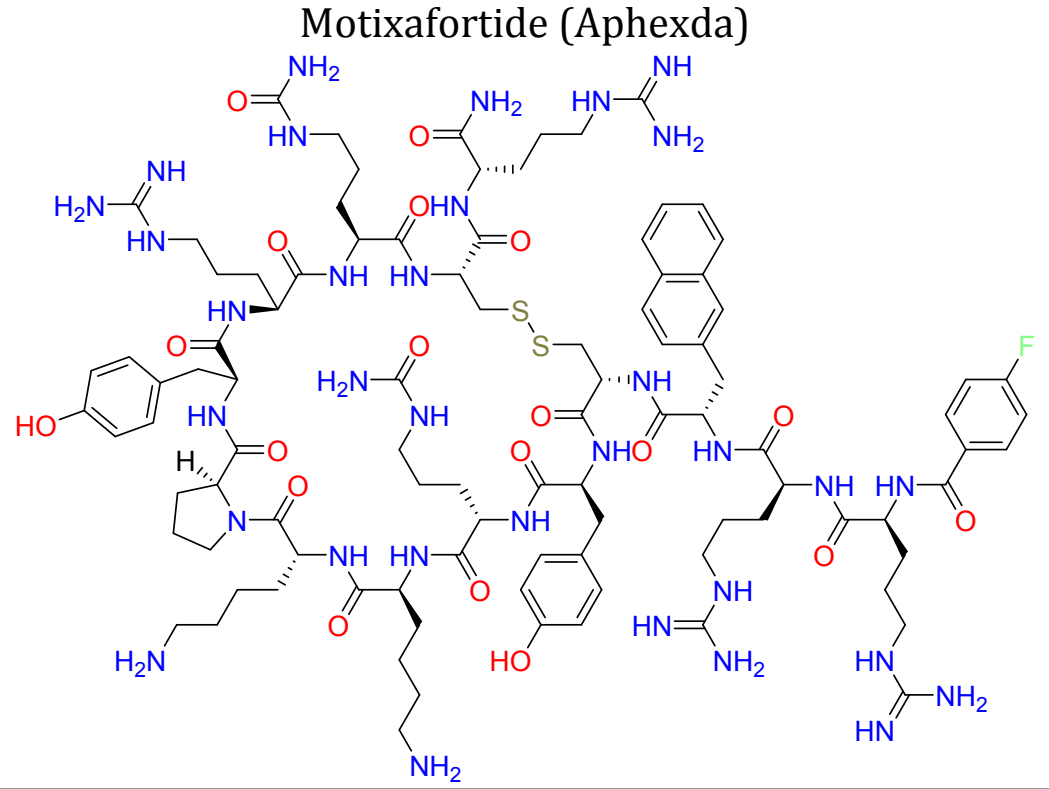	664334-36-5	52	43	53	-6.742	2159.53	560.61	152	942.64	51.03	NR
Zilucoplan (Zilbrysq) 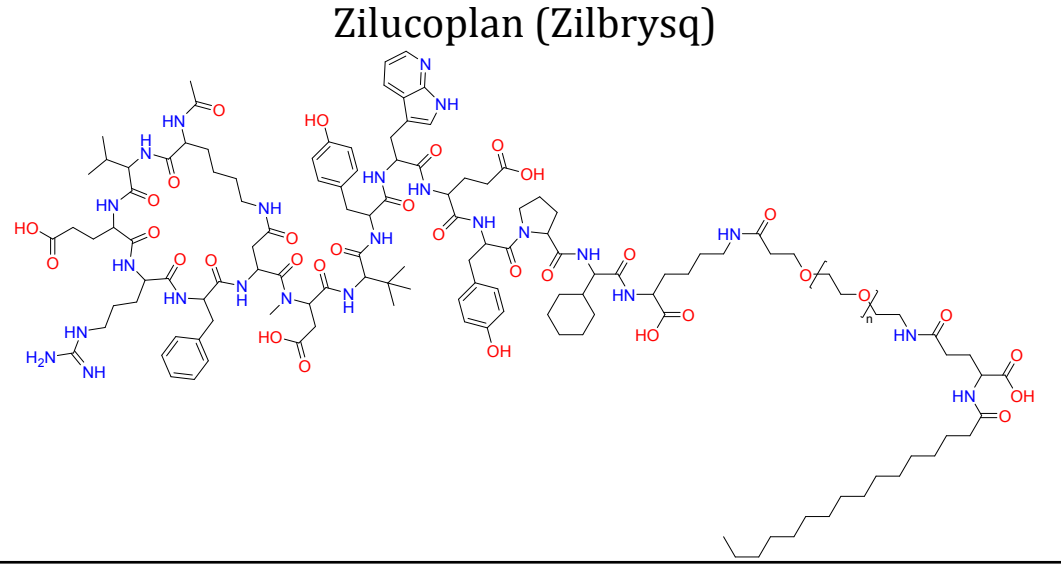	1841136-73-9	NR	NR	NR	NR	NR	NR	NR	NR	100	NR
Voclosporin (Lupkynis)	515814-01-4	23	5	16	1.85	1214.62	332.68	86	278.8	100	13.315±0.70

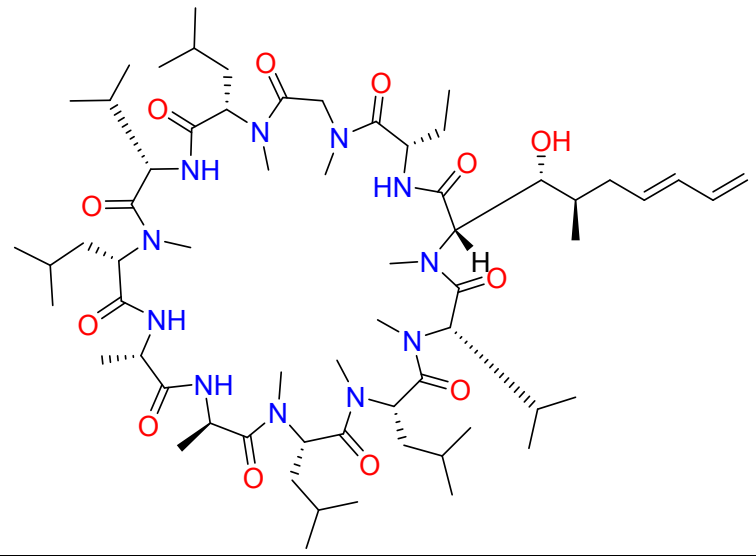										
Setmelanotide (Imcivree) 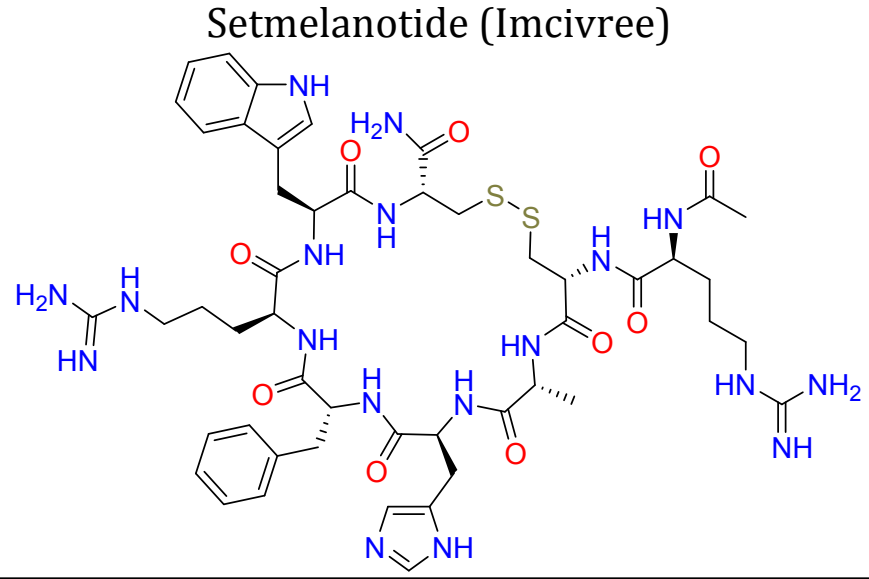	920014-72-8	27	20	20	-3.175	1117.31	290.34	78	497.76	23.15	12.818±0.70
Bremelanotide (Vyleesi) 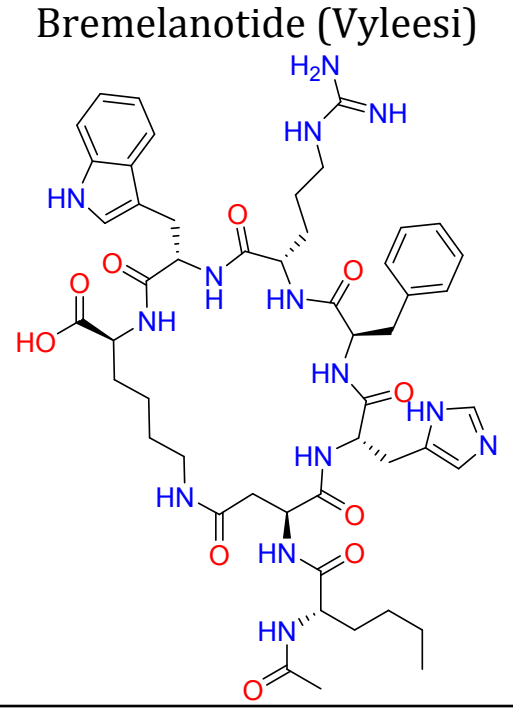	189691-06-3	24	15	18	-1.14	1025.16	268.58	74	376.47	53.24	3.371±0.70
177Lu-DOTATATE (Lutathera) 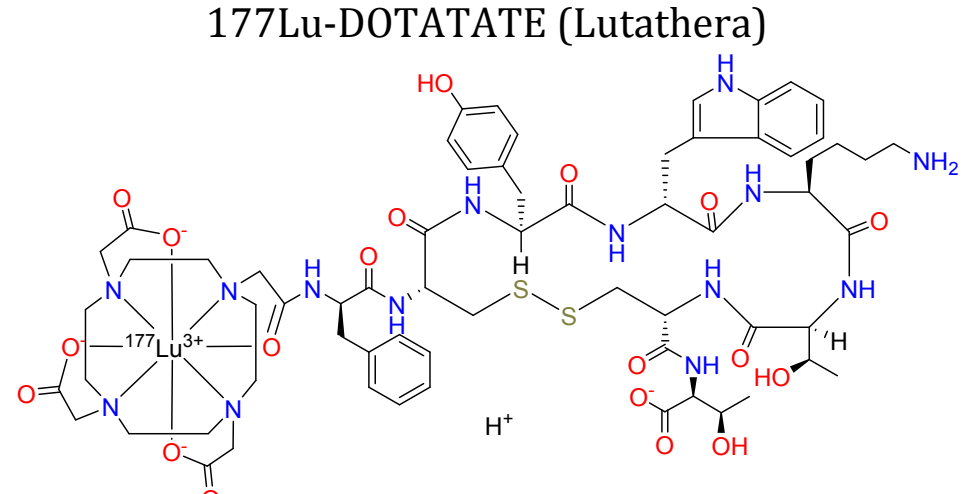	437608-50-9	NR	NR	NR	NR	1609.55	343.19	101	NR	NR	NR
Plitidepsin (Aplidin)	137219-37-5	22	4	15	1.0003	1110.34	288.59	79	284.74	99.94	11.278±0.70

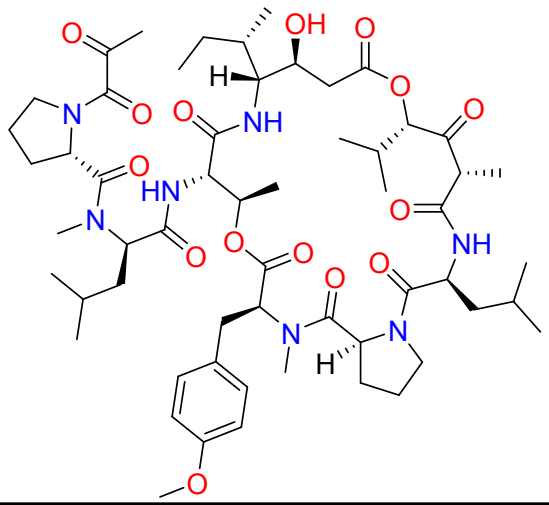											
Plecanatide (Trulance) 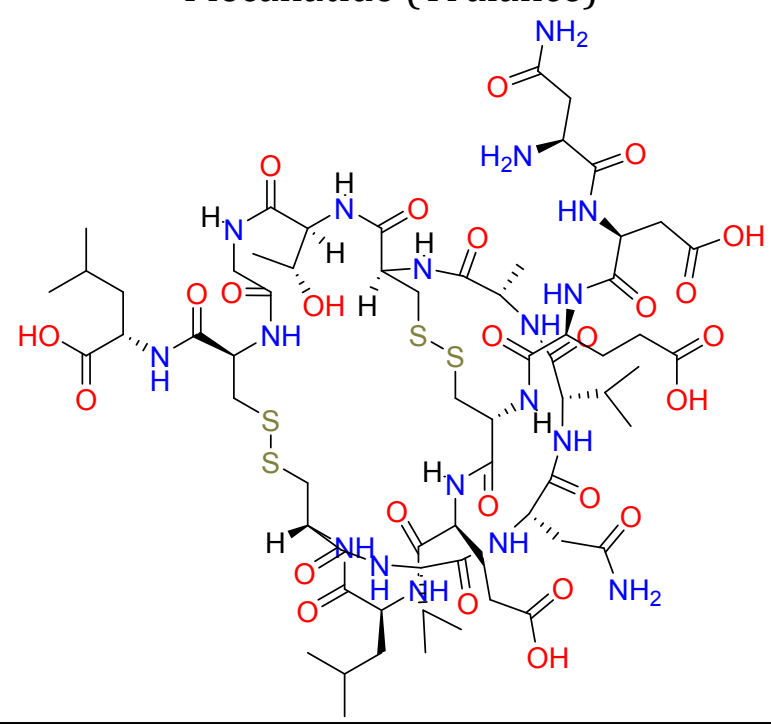	467426-54-6	44	26	28	-5.693	1681.89	398.46	113	819.33	0	3.606±0.21
Voxilaprevir (Vosevi) 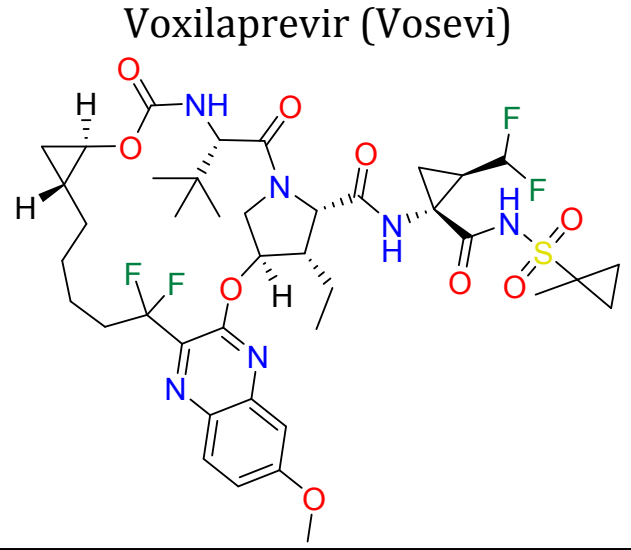	1535212-07-7	15	3	9	4.171	868.94	202.66	60	203.6	95.48	5.485±0.40
Grazoprevir (Zepatier) 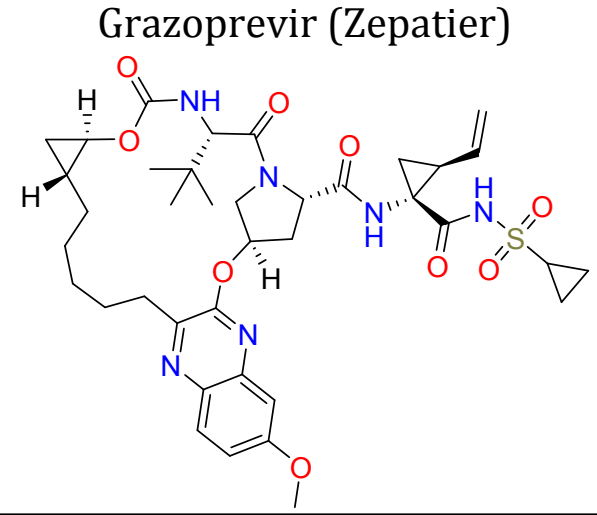	1350514-68-9	15	3	8	3.935	766.91	193.37	54	203.6	73.86	5.511±0.40
Oritavancin (Orbactiv)	171099-57-3	36	22	19	2.651	1793.1	440.43	125	560.98	100	2.929±0.70

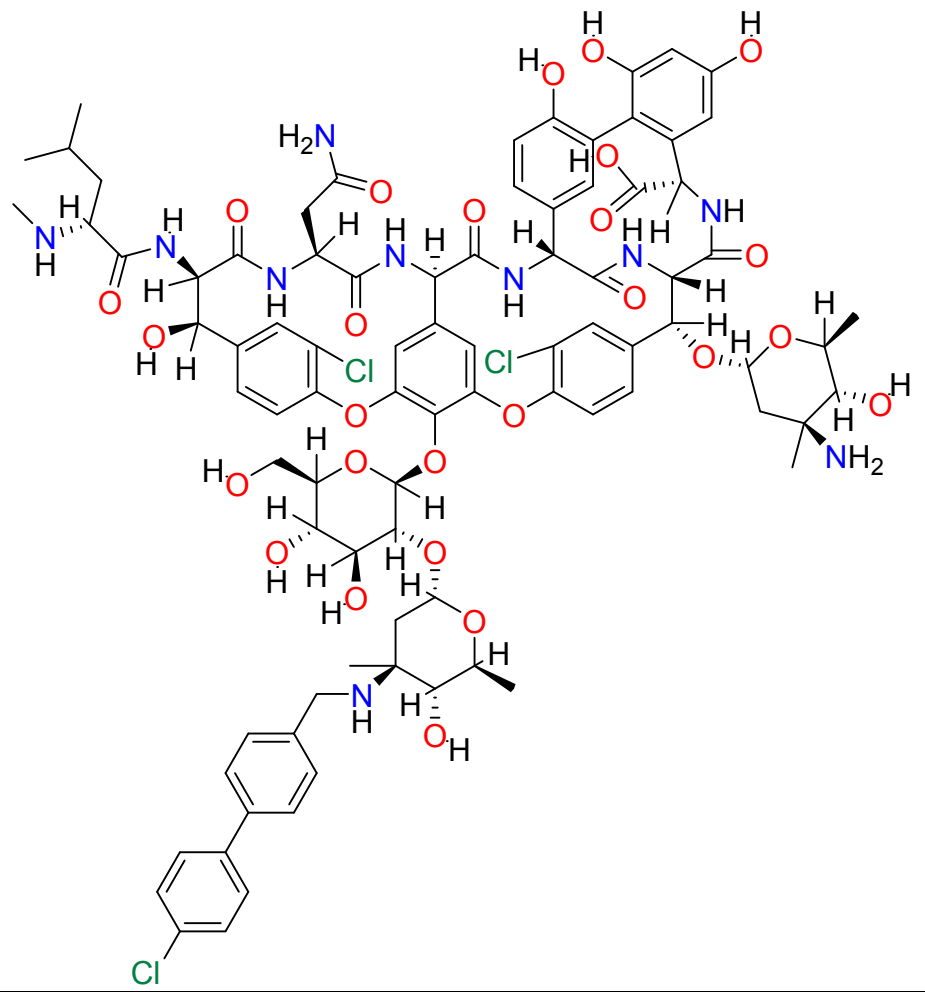											
Terlipressin (Glypressin)<br>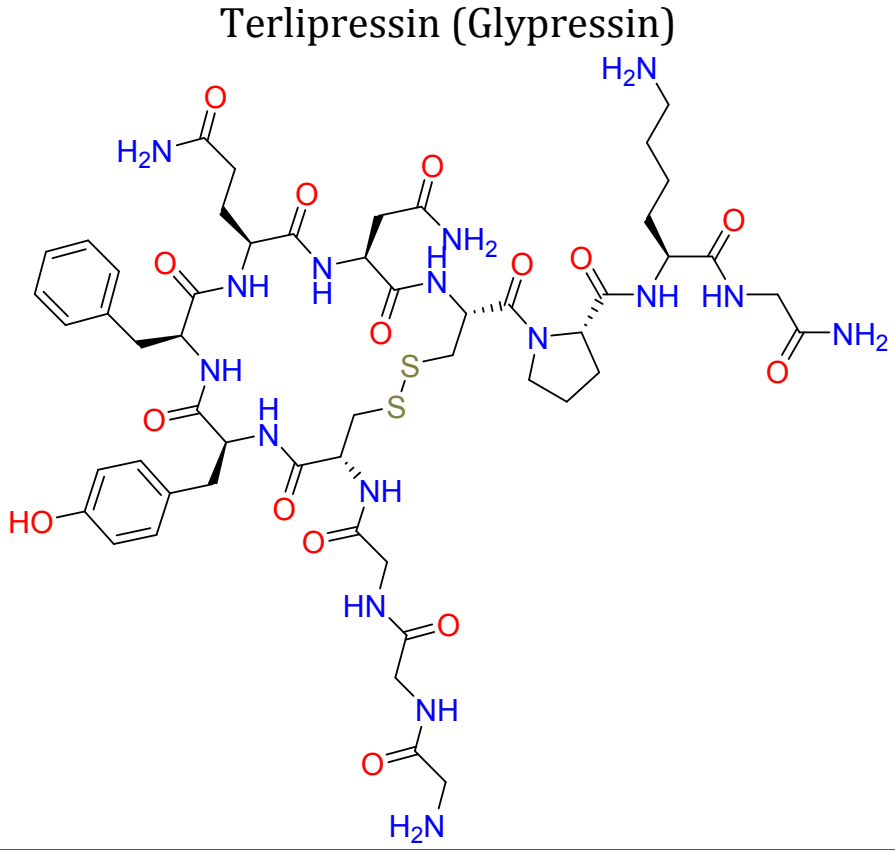	14636-12-5	31	21	25	-7.085	1227.38	305.69	85	563.45	0	9.901±0.15
Linaclotide (Linzess)	851199-59-2	36	21	13	-6.127	1526.75	367.4	101	725.71	0	3.053±0.10

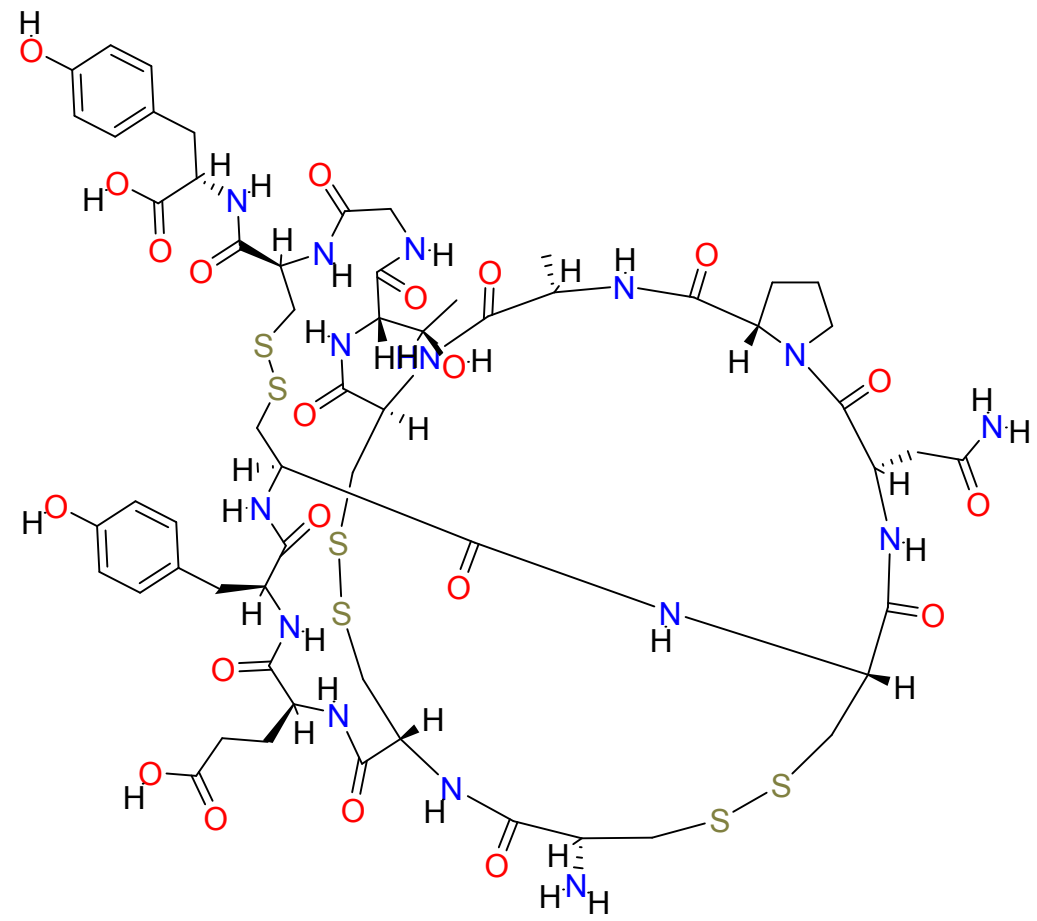											
Pasireotide (Signifor) 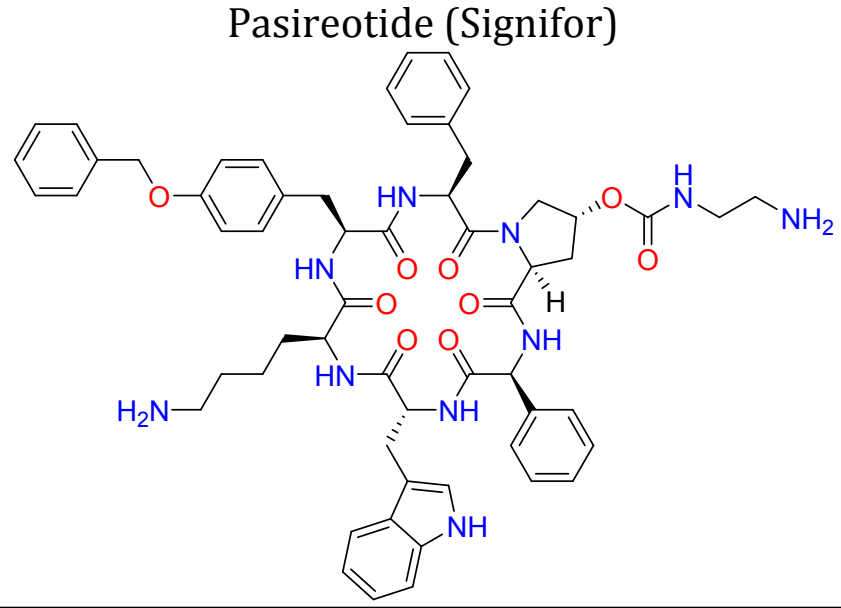	396091-73-9	19	11	18	2.712	1047.21	287.04	77	281.2	87.61	11.858± 0.46

References:

(1) *RDKit: Open-Source Cheminformatics Software*. https://www.rdkit.org/ (accessed 2025 30th December).
(2) *ACD/Labs*. https://www.acdlabs.com/ (accessed 2026 April).
